# Femtojoule optical Kerr switching with milliwatt-peak-power in silicon-organic hybrid nanocavity

**DOI:** 10.1038/s41467-026-73285-9

**Published:** 2026-05-15

**Authors:** Yizheng Chen, Xiaoyan Gao, Gaoneng Dong, Wentao Gu, Jianhua Ning, Wentao Ye, Yilun Wang, Wenchan Dong, Lei Lei, Jing Xu, Xinliang Zhang

**Affiliations:** 1 Wuhan National Laboratory for Optoelectronics & School of Optical and Electronic Information, Huazhong University of Science and Technology, Wuhan, China; 2 State Key Laboratory of Radio Frequency Heterogeneous Integration (Shenzhen University), Shenzhen, China; 3 Hubei Optical Fundamental Research Center, Wuhan, China; 4 Optics Valley Laboratory, Wuhan, China

**Keywords:** Integrated optics, Polymers, Photonic crystals, Nonlinear optics

## Abstract

All-optical Kerr switches enable ultrafast switching speeds, essential for next-generation communication and computing. However, their practical deployment is hindered by the intrinsically weak optical nonlinearity, which necessitates high switching energies. Although ultrashort pulses with Watt-level peak powers and extremely low duty cycles can alleviate this requirement, they are incompatible with real-world systems that demand high-duty-cycle data. In this work, we overcome this long-standing challenge by experimentally demonstrating an optical Kerr switch compatible with mainstream communication signals, while maintaining femtojoule-level switching energy. The breakthrough is achieved through a combination of exceptionally tight optical field confinement and a high-nonlinearity polymer material in a silicon-organic hybrid slot nanobeam cavity. This design reduces the required peak power to milliwatt-level, approximately two orders of magnitude lower than previous Kerr switches, enabling error-free switching of a 33%-duty-cycle 40-Gbit/s signal with 7.5 mW peak power and 63 fJ/bit switching energy. These achievements pave the way for chip-scale all-optical switches for ultrafast photonic signal processing.

## Abstract (web summary)

This study demonstrates a breakthrough in all-optical Kerr switches by a silicon-organic hybrid slot nanobeam cavity. It achieves 63 fJ/bit switching energy with 7.5 mW peak power, enabling error-free switching of high-duty-cycle signals.

## Introduction

All-optical switches—a fundamental photonic building block for light-controlled operations—are essential to on-chip ultrafast optical interconnection networks^1–3^, all-optical information processing^4–6^, and photonic neural networks^7–10^. Their transformative potential lies in the ability to circumvent the bandwidth limitations inherent in conventional optoelectronic systems. A wide range of high-speed all-optical switches have been demonstrated, harnessing diverse fast optical nonlinearities, such as χ^(2)^ and χ^(3)^ nonlinearities^5,6,11–21^, saturable absorption^22–25^, and inter-sub-band transition^26,27^. These devices can operate with femtojoule-level switching energy and picosecond-scale or shorter switching windows, demonstrating significant potential for energy-efficient photonic systems and terabit-class signal processing. However, the majority of demonstrated devices rely on excitation by ultrashort pulses with Watt-level peak powers and extremely low duty cycles (defined as the ratio of the pulse duration to the repetition period). This reliance arises not only from the intrinsically weak nonlinearities of many integrated platforms, but also from the broader challenge of simultaneously enhancing light-matter interaction and maintaining low optical loss—even in materials that offer relatively strong nonlinearities^4,11,22,28^. While such low duty cycles help maintain low average power—thereby minimizing thermal effects and preventing device damage at elevated average power levels—they impose substantial limitations for practical implementations. In particular, communication and computing systems typically require high-duty-cycle operation with GBaud-rate signals and milliwatt-level peak powers^29^, making current device architectures unsuitable for many real-world applications. Moreover, superior switching performances that could achieve error-free performance are essential in real systems, which require both high switching contrast and small signal distortion—parameters that are often traded off against switching energy. Therefore, the development of high-performance all-optical switches that simultaneously achieve ultra-low switching energy and ultra-low peak power holds the key to realizing on-chip ultra-efficient photonic processing systems, yet remains an unresolved challenge.

Among various ultrafast switching mechanisms, the optical Kerr effect that originates from χ^(3)^ nonlinearities, inherently satisfies the stringent requirements of switching performance thanks to its intrinsic ultrafast response reaching the femtosecond regime^30–33^. To enhance weak χ^(3)^ optical nonlinearity, a wide array of integrated nonlinear photonic platforms has been extensively explored in recent years, including silicon^34–36^, silicon nitride^37–39^, lithium niobate^40,41^, aluminum gallium arsenide^42^, and silicon carbide^43^. However, the material selection for Kerr-based switching is fundamentally governed by a balance between nonlinear strength and nonlinear absorption^44^. This trade-off originates from the intrinsic correlation between the nonlinear refractive index and the linear refractive index, as described by empirical scaling relations following Miller’s rule. As the refractive index increases, the nonlinear refractive index is typically enhanced, but this trend is also accompanied by a reduced bandgap energy, leading to strong two-photon absorption (TPA) and subsequent free-carrier absorption (FCA) at telecommunication wavelengths in high refractive index materials such as silicon^45–47^. To overcome these intrinsic limitations, the recently proposed silicon-organic hybrid (SOH) integration scheme has emerged as a promising alternative, attracting considerable attention^48,49^. This comes from the fact that organic nonlinear materials combine a relatively low refractive index with an exceptionally high nonlinear refractive index, thereby deviating significantly from the Miller scaling. By integrating such materials into silicon slot waveguides, SOH designs enable ultra-tight transverse optical confinement with the TPA- and FCA-free ultra-high Kerr nonlinearity of the organic materials^33,50,51^, by making use of the high refractive index of silicon while avoiding the nonlinear absorption of silicon. An additional advantage of the silicon platform is its native compatibility with complementary metal oxide semiconductor (CMOS) fabrication processes^52,53^ (see extended discussions in Supplementary Information S1). These salient features make SOH nanostructures ideal candidates for high-speed all-optical signal processing applications. Nevertheless, all-optical switches based on SOH designs reported to date have yet to meet the stringent requirements for simultaneously achieving both ultra-low switching energy and ultra-low peak power, largely because further enhancement of the optical nonlinearity remains challenging within conventional slot-based structures^17,33^.

**Fig. 1:**
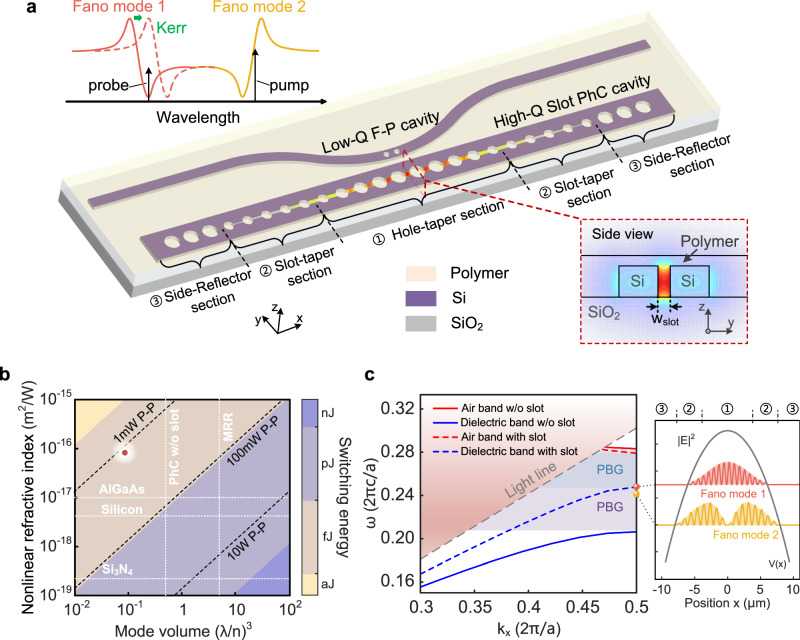
Schematic and simulation of a polymer-coated slot nanocavity for all-optical control. **a** Schematic of an SOH Kerr all-optical switch based on a PhC slot nanobeam cavity. The nonlinear organic polymer material used is 2-methoxy-5-(2-ethylhexyloxy)−1,4-phenylenevinylene (MEH-PPV). The cavity consists of three main sections: the hole-taper section, the slot-taper section, and the side-reflector section. The inset illustrates the operating principle of two-mode all-optical switching through cross-phase modulation induced by the Kerr effect. The red dashed box highlights the calculated electric field distributions ($\left|E\right|$) at the central cross-section of the slot PhC nanobeam cavity. **b** Calculated switching energy for various mode volume $V$ and nonlinear refractive index ${n}_{2}$, assuming the loaded *Q*-factor (${Q}_{{{\mathrm{loaded}}}}$) is sufficient to support a 40 GBaud switching speed. The black dashed line represents the peak power corresponding to the switching energy, while the white dotted lines indicate different materials (horizontal) and structures (vertical). The red dot marks the operating region of the proposed device. **c** Band diagrams of the proposed PhC structures with (dashed curves) and without (solid curves) a slot, corresponding to the cavity center and the side-reflector section, respectively. PBG photonic bandgap. Right: the calculated effective parabolic potential for photons (gray solid lines) and field amplitude distributions of Fano mode 1 and mode 2 in the slot PhC nanobeam cavity (red lines and yellow lines, respectively).

In this work, we propose and experimentally demonstrate a femtojoule-level optical Kerr switch operating at milliwatt peak power based on a SOH nanocavity. The nanocavity is designed as a photonic crystal (PhC) slot nanobeam cavity (Fig. 1a). The combination of the slot structure and the nanobeam cavity results in a sharply compressed mode volume ($V$) in both the longitudinal and transverse directions, significantly lowering switching energy as the intracavity field intensity ${\left|E\right|}^{2}$ scales with $1/V$. By further incorporating the ultra-high nonlinear polymer, our proposed device stands out in terms of switching energy (Fig. 1b), owing to an exceptionally large ${n}_{2}/V$ value (marked by the red dot) compared to existing methods, where ${n}_{2}$ is the nonlinear refractive index and ${n}_{2}/V$ is known to be essential for achieving low switching energy and peak power (see Supplementary Information S2 for details). Although a trade-off between speed and switching energy typically exists in cavity-related structures, high-speed operation at 40 GBaud with 33% duty cycle is realized by carefully designing the nanocavity with moderate-loaded quality (*Q*) factor while maintaining a high intrinsic *Q*-factor. Error-free performance with a switching contrast of 8.3 dB is demonstrated at a peak power as low as 7.5 mW, which is two orders of magnitude lower compared to previously reported Kerr switches. The achieved switching energy of only 63 fJ/bit further highlights the exceptional efficiency of on-chip operation. We envision that the proposed high-speed, energy-efficient SOH Kerr all-optical switch holds strong promise for practical application scenarios, such as data center interconnects, high-speed optical communications, and optical computing.

## Results

### Principle and design

As shown in Fig. 1a, the proposed Kerr switch combines an SOH slot structure with a PhC nanobeam cavity design. The PhC nanobeam cavity is renowned for its ultra-high *Q*-factor and extremely small mode volume $V$ comparable to ${(\lambda /n)}^{3}$ ($\lambda$ is the wavelength of light, $n$ is the refractive index), making it highly favorable for enhancing optical nonlinearity to enable efficient all-optical switching^54–56^. The electric field distribution at the central cross-section of the device (yz-plane, slot width ${w}_{{{{\rm{slot}}}}}=80\,{{{\rm{nm}}}}$) is shown in the lower-right inset of Fig. 1a, where the electromagnetic boundary conditions lead to a strong field discontinuity, resulting in a sharply compressed mode area within the slot^57^. Full-wave simulation (FDTD) further reveals ultra-tight optical confinement with a mode volume as small as ${0.09(\lambda /n)}^{3}$ (Supplementary Information S5), which is 4.5 times smaller compared to that of the nanobeam cavity without a slot. The nonlinear organic polymer used in this work exhibits a nonlinear refractive index as high as ${n}_{2}=(8.5\pm 0.4)\times {10}^{-17}\,{{{{\rm{m}}}}}^{2}/{{{\rm{W}}}}$ at 1550 nm with negligible nonlinear absorption^50^, which is about 20 times higher than that of silicon^47^. From a wave-guiding perspective, the nonlinear coefficient of the PhC slot waveguide is estimated to be 2952 m^−1^ W^−1^, which is approximately 31 times higher than that of the strip silicon waveguide, thanks to a higher ${n}_{2}$ and smaller effective mode area (see Supplementary Information S6 for details). Moreover, a bus waveguide with two air holes is positioned adjacent to the nanobeam cavity in order to enable optical coupling and regulate the loaded *Q*-factor of the cavity, as explained later. The two air holes in the bus waveguide are added to produce asymmetric Fano line shapes, a well-established technique to enhance the switching contrast and reduce switching energy by forming a low-Q Fabry–Pérot (F–P) cavity that interferes with the high-Q nanobeam cavity^58–60^.

The operating principle of the all-optical Kerr switch is illustrated in the upper-left inset of Fig. 1a, which relies on the nonlinear interaction between two Fano-resonant modes formed in the nanocavity (red and yellow). For switching, a weak probe light and a strong pump light are simultaneously injected into the bus waveguide. The pump light at Fano mode 2 generates a significant phase-shift in the nanocavity by changing the refractive index of the polymer through the Kerr effect, red-shifting the resonance of Fano mode 1, known as cross-phase modulation (XPM)^61^. As a result, the transmission of the probe light can be switched from minimum to maximum in the presence of the pump light. Meanwhile, a sufficient spatial overlap between the two Fano modes ensures efficient XPM experienced by the probe light. Compared to single-resonance operation, where the pump and probe light share the same resonance mode^1,14–17^, the two-mode configuration enables effective separation in frequency, thereby allowing the full utilization of resonance bandwidth and suppression of nonlinear cross-talk.

Next, we elaborate on the key elements in the design of the slot nanobeam cavity. Theoretical analysis shows that achieving a high intrinsic *Q*-factor (${Q}_{i}$) is crucial for simultaneously enhancing the extinction ratio (${ER}$) of the transmission spectrum and the field enhancement factor (${FE}$)—two parameters that fundamentally determine the switching contrast and nonlinear efficiency in all-optical switching (see Supplementary Information S2 for details). Consequently, an optimized PhC slot nanobeam cavity is required to support high-${Q}_{i}$ resonances while achieving deep-subwavelength mode confinement within the nonlinear polymer via the slot structure. To this end, the nanocavity is designed to have three different sections, i.e., hole-taper section, slot-taper section, and side-reflector section, where the side-reflector sections have no slot penetration. As shown in Fig. 1c, the transverse-electric (TE) band diagrams of the designed PhC with and without slot—corresponding to the cavity center and the side-reflector sections, respectively—indicate that the introduction of the slot reduces the photonic bandgap (PBG) and weakens optical confinement. Therefore, the slot-free side-reflector sections are employed to create strong reflection and sustain a high ${Q}_{i}$. However, the abrupt spatial variations in the mode field envelope between slot and non-slot regions introduce mode mismatch, fundamentally constraining the ${Q}_{i}$. To address this limitation, hole-taper and slot-taper regions are incorporated to independently introduce gradual variations in hole radius and slot width, respectively, enabling smooth mode transitions between regions. This design effectively shapes the mode field envelope into a near-Gaussian profile, thereby reducing transmission loss^62,63^ (see Supplementary Information S5 for detailed design parameters). Full-wave FDTD simulations show that the designed Fano mode 1 exhibits a ${Q}_{i}$ of $6.6\times {10}^{5}$ and a mode volume of $0.074\,{{{{\rm{\mu }}}}{{{\rm{m}}}}}^{3}\approx 0.09{(\lambda /n)}^{3}$, while Fano mode 2 achieves a ${Q}_{i}$ of $2.1\times {10}^{5}$ and a mode volume of $0.194\,{{{{\rm{\mu }}}}{{{\rm{m}}}}}^{3}\approx 0.23{(\lambda /n)}^{3}$. The corresponding field amplitude distributions of the central cross-section along the x-direction of the slot PhC nanobeam cavity are presented in the right panel of Fig. 1c (More details are given in Supplementary Information S5). Based on their spatial distributions, the electric field overlap integral (${C}_{{{\mathrm{Overlap}}}}$) is calculated to be 0.69.

Finally, the coupling of the bus waveguide to the nanobeam cavity is considered since it is crucial for determining the operation speed of the switch. To align with the typical Baud rate of modern optical communication systems, we target a switching speed of 40 GBaud. This operational requirement necessitates a cavity optical bandwidth of at least 40 GHz, corresponding to a loaded *Q*-factor (${Q}_{l}$) of less than approximately $4.8\times {10}^{3}$. Achieving this specification requires careful selection of the external coupling *Q*-factor (${Q}_{e}$), as it determines the total loaded *Q*-factor through the relation $1/{Q}_{l}=1/{Q}_{e}+1/{Q}_{i}$. Since field enhancement scales with $Q/V$, a higher ${Q}_{l}/V$ ratio is desirable to obtain a lower switching energy. Given that $V$ has already been as small as possible through cavity design, further improvement in energy efficiency necessitates precise tuning of ${Q}_{e}$ to strike a balance between speed and energy consumption. Theoretical modeling reveals that the coupling efficiency is primarily influenced by the bus waveguide width and its separation from the cavity. Additionally, the size and spacing of the two air holes in the low-Q F–P cavity play a key role in shaping the Fano resonance while exerting a slight effect on the coupling efficiency (see Supplementary Information S5 for more design details). Through systematic parameter optimization, the designed ${Q}_{l}$ for the Fano mode 1 and mode 2 are $5.3\times {10}^{3}$ and $7.0\times {10}^{3}$, respectively, slightly higher than the ideal requirements to compensate for the fabrication-induced Q degradation.

### Experiment

**Fig. 2:**
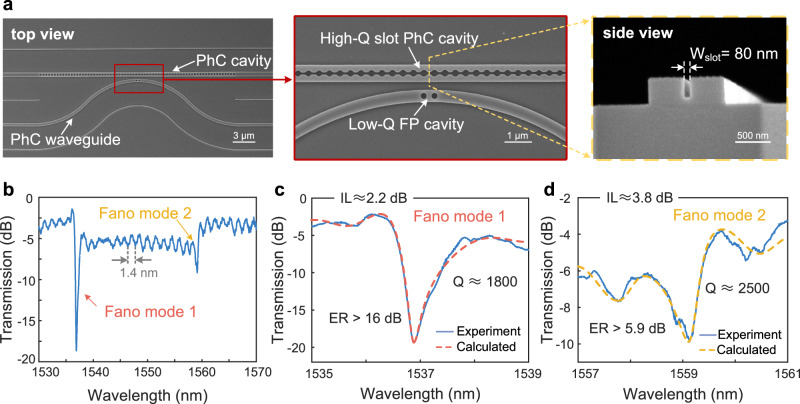
SEM images and transmission spectra of the fabricated samples. **a** SEM top view of the PhC cavity and the PhC waveguide (left). The SEM image in the center is a magnified view of the solid red box in the left image. The right image shows the cross-sectional SEM view of the central slot before spin-coating the polymer, taken at the dashed yellow line in the center image. Scale bar: 3 μm (left), 1 μm (center), 500 nm (right). **b** Experimental measured transmission spectrum, exhibiting two distinct Fano resonances. The small periodic ripples with a characteristic period of 1.4 nm in the spectrum is attributed to F–P oscillations induced by reflections between the air holes of the bus waveguide and the input/output facets. **c** Experimental (blue solid line) and calculated (red dashed line) transmission spectrum of Fano mode 1. The calculated loaded *Q*-factor, extinction ratio (${ER}$), and insertion loss (${IL}$) for Fano mode 1 are 1.8 × 10^3^, 16 dB, and 2.2 dB, respectively. **d** Experimental (blue solid line) and calculated (yellow dashed line) transmission spectrum of Fano mode 2. The calculated loaded *Q*-factor, ${ER}$, and ${IL}$ for Fano mode 2 are 2.5 × 10^3^, 5.9 dB, and 3.8 dB, respectively. All insertion loss values refer to on-chip loss, excluding grating-coupler losses.

The optical Kerr switches are fabricated through the in-house process platform (Supplementary Information S7) to experimentally demonstrate the proposed scheme. Figure 2a presents the top-view and cross-sectional scanning electron microscopy (SEM) images of the fabricated device, which is patterned via electron beam lithography (EBL) followed by etching with a magnetic neutral loop discharge (NLD) plasma etcher. The narrowest slot width is only 40 nm, exhibiting excellent sidewall verticality (see Supplementary Information S7 for details), with a final slot depth achieved through controlled over-etching. This compensation strategy mitigates the aspect-ratio-dependent etching effect^64^, where the etching rate within high-aspect-ratio slots (${h}_{{{{\rm{sl}}}}{{{\rm{ot}}}}}/{w}_{{{{\rm{sl}}}}{{{\rm{ot}}}}}\approx 5.5$) is reduced by $30\%$ compared to open regions. Subsequently, the dissolved MEH-PPV is spin-coated onto the device, forming a polymer layer of approximately 300 nm thick. The experimentally measured transmission spectrum of the device is shown in Fig. 2b, revealing two distinct Fano resonances at 1536.89 nm (Fano mode 1) and 1559.13 nm (Fano mode 2) within the C-band (1530–1565 nm), both exhibiting characteristic sharp asymmetric Fano line shapes. The opposite resonance features of these two Fano modes arise from their distinct spatial mode symmetries, with Fano mode 1 and Fano mode 2 corresponding to even and odd symmetry distributions, respectively^65^. Figure 2c, d present magnified views of the two resonances, where the extinction ratios, defined as the transmission contrast between the peak and dip, are measured to be 16 dB and 5.9 dB for Fano mode 1 and mode 2, respectively. The corresponding insertion losses at the resonance peaks are 2.2 dB and 3.8 dB. Based on the transmission spectrum and theoretical model fitting (Supplementary Information S2), the ${Q}_{i}$ of Fano mode 1 and mode 2 are calculated as $1.3\times {10}^{4}$ and $4.5\times {10}^{3}$, respectively. The ${Q}_{l}$ are $1800$ and $2500$, corresponding to optical bandwidths of approximately 108 GHz and 77 GHz, respectively, which are sufficient for 40 GBaud operation. The degradation of the measured *Q*-factors compared to the simulated values is attributed to polymer coating non-uniformity, as well as fabrication-induced sidewall roughness. Nevertheless, the achieved *Q*-factors are sufficient to support low-energy switching operation (possible optimization processes are discussed in Discussion). The optical bandwidth is calculated via $\Delta v=c/({\lambda }_{{{{\rm{res}}}}}\bullet {Q}_{l})$, where $c$ is the speed of light in vacuum. These bandwidths correspond to photon lifetimes (${\tau }_{{ph}}$) of 1.5 ps and 2.1 ps, determined by ${\tau }_{{ph}}=1/2\pi \Delta v$, which define the temporal response of the cavity. Since the response of Kerr nonlinearity of MEH-PPV is on the orders of femtoseconds^66^, the switching time of the device is ultimately limited by the photon lifetime of the Fano resonances rather than the material response, thereby enabling few-picosecond operation in this design.

**Fig. 3:**
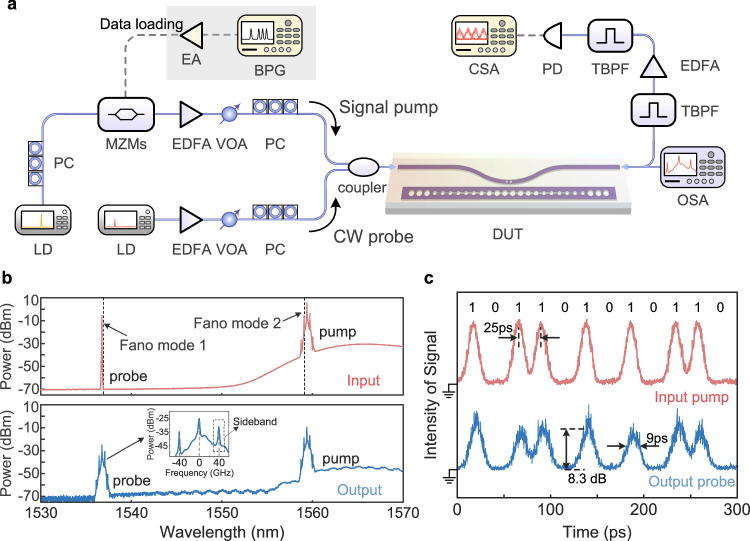
Demonstration of two-mode all-optical Kerr switching. **a** Experimental setup for the all-optical switching measurement. LD laser device, PC polarization controller, MZMs Mach–Zehnder modulators, BPG bit pattern generator, EA electrical amplifier, EDFA erbium-doped fiber amplifier, VOA variable optical attenuator, DUT device under test, TBPF tunable bandpass filter, PD photodiode, CSA communication signal analyzer, OSA optical spectrum analyzer. An EDFA is used to amplify the output probe signal^69^. **b** Input spectrum (red) and the output spectrum after the device (blue), measured with a resolution bandwidth of 0.02 nm. The black dashed lines in the upper panel indicate the resonant wavelengths of Fano mode 1 and Fano mode 2, respectively. The inset in the bottom panel shows the modulated probe light with distinct modulation sidebands. The input pump energy is 63 fJ/bit, corresponding to a peak power of 7.5 mW. **c** Input pump pulse train (red) and output probe pulse train (blue), both with a pulse width of 9 ps and a period of 25 ps (40 GBaud signal rate). The output probe pulse is correctly switched in response to the pump pulse, exhibiting a switching contrast of 8.3 dB.

To further investigate the dynamic characteristics of the device, we experimentally evaluate its switching performance with a 40 GBaud optical signal (results at other rates are provided in Supplementary Information S10 for details). The experimental setup is shown in Fig. 3a. A 2^7^-1 pseudorandom binary sequence (PRBS) electrical signal at 40 GBaud is generated by a bit pattern generator (BPG), and used to drive Mach–Zehnder modulators (MZMs), producing a 40 Gbit/s return-to-zero on-off keying (RZ-OOK) optical signal with a duty cycle of 33%. The modulated signal serves as the pump light, while a separate continuous-wave (CW) laser provides the probe light. The probe power is approximately -5 dBm, which is much lower than the pump power, ensuring that the nonlinear Kerr phase shift is predominantly induced by the pump. In the experiment, both pump and probe light are simultaneously coupled into the nanocavity, where the Kerr effect in the organic polymer modulates the transmission of the probe light. The power of the pump light is controlled using an erbium-doped fiber amplifier (EDFA) and a variable optical attenuator (VOA). At the output, a tunable bandpass filter (TBPF) is employed to extract the probe light, effectively suppressing crosstalk from the pump light thanks to the large resonance spacing of 22 nm between Fano mode 1 and Fano mode 2. Finally, the time domain characteristics and eye diagram of the transmitted signal are analyzed using a communication signal analyzer (CSA).

Figure 3b presents the input (upper panel) and output (bottom panel) spectrum measured by an optical spectrum analyzer (OSA), corresponding to an on-chip input peak power of 7.5 mW for the pump light. With an estimated switching window from the duty cycle, as well as the signal rate, i.e., $33\%/\left(40\,{{{\rm{Gb}}}}/{{{\rm{s}}}}\right)\approx 8.3\,{{{\rm{ps}}}}$, the corresponding switching energy (defined as the energy of each “1” bit of pump light on-chip) is 63 fJ/bit, calculated as $7.5\,{{{\rm{mW}}}}\times 8.3\,{{{\rm{ps}}}}\approx 63\,{{{\rm{fJ}}}}/{{{\rm{bit}}}}$. The black dashed lines indicate the resonance wavelengths of Fano mode 1 and Fano mode 2, respectively. The wavelengths of the pump and probe lights are blue-shifted by 0.15 nm relative to the initial operating wavelength to compensate for the thermo-optic effect of the polymer (see Supplementary Information S8 for details). The output spectrum shows that the modulation sidebands of the signal light have been perfectly copied onto the probe light, with the first-order sideband separated from the main peak by 40 GHz, as shown in the inset. The temporal responses of the probe light measured by the CSA are shown in Fig. 3c. The red and blue solid traces represent the original 40 GBaud pump signal and the corresponding modulated probe output, respectively. Clear data transfer from pump to probe is observed via XPM induced by “1” bits, with each bit exhibiting a switching window of approximately 9 ps. The observed small inter-crosstalk interference and pattern effect are primarily due to the virtually instantaneous Kerr nonlinear response and the carefully optimized optical bandwidth characteristics.

**Fig. 4:**
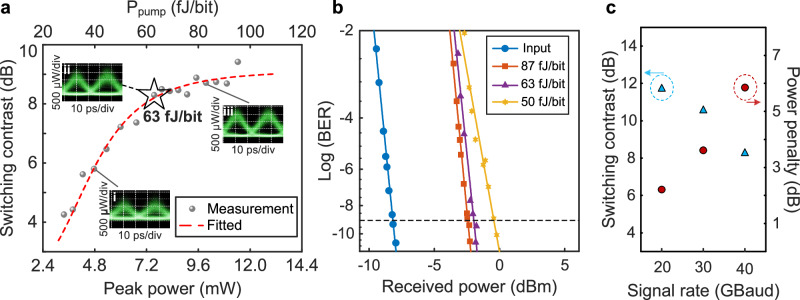
Femtojoule 40 GBaud all-optical switching with error-free performance. **a** Switching contrast as a function of switching energy, with eye diagrams at 40 fJ/bit (i), 63 fJ/bit (ii), and 87 fJ/bit (iii). The red dashed line represents a fitted trend of the experimental results (gray dots). **b** BER curves of original pump light and the modulated probe light at switching energies of 50 fJ/bit, 63 fJ/bit, and 87 fJ/bit. **c** Switching contrast (blue triangles) and power penalty (red dots) at different signal rates.

Figure 4a shows the evolution of the switching contrast as the peak power of the pump light increases. It can be seen that the switching contrast initially rises with increasing pump power and eventually saturates. The saturation threshold is defined as the minimum pump power required for the switching contrast to reach 90% of its saturation value, which represents the optimal operating condition of the switch. The switching contrast achieves 5.8 dB at 40 fJ/bit and a peak power of 4.8 mW, with the eye diagram exhibiting a thick upper eyelid (inset i), which corresponds to a *Q* value of 2.3 and a signal-to-noise ratio (SNR) of 7.2 dB. Here, the *Q* value of the eye diagram is defined as the ratio of the signal amplitude to the standard deviation of the noise, while the SNR is defined as the ratio of the signal power to the noise power^67^. As the switching energy increases, the switching contrast continues to improve, reaching 8.3 dB at 63 fJ/bit and a peak power of 7.5 mW. The eye diagram at this point (inset ii) demonstrates high signal quality, with a *Q* value of 3.4 and an SNR of 10.5 dB, reflecting a significant improvement in both signal clarity and noise margin. At this stage, the system reaches the saturation threshold, where further increases in energy no longer result in substantial improvements. As shown in inset iii, both the switching contrast and the eye diagram quality become saturated, where the *Q* value is approximately 3.6, and the SNR is 11.1 dB. This is because once the pump energy is strong enough to shift the probe light completely out of the resonance window, further increase in pump energy has little impact on the switching contrast. The overall trend of the experimental results aligns well with the theoretical predictions based on nonlinear time-domain coupled-mode equations (see Supplementary Information S4 for details).

To further demonstrate the system performance of the device in high-speed communication, the bit error rate (BER) curves of both the original pump light and the modulated probe light at different switching energies are measured, as shown in Fig. 4b. Error-free performances are realized (${{{\rm{BER}}}} < {10}^{-9}$) when the switching energy exceeds 50 fJ/bit. The BER curves exhibit similar slopes and are nearly parallel to that of the original signal, indicating that the switched signals maintain a comparable noise distribution. Furthermore, the received power required to achieve ${{{\rm{BER}}}}={10}^{-9}$ decreases significantly with increasing switching energy, following a trend similar to the switching contrast and eye diagram evolution shown in Fig. 4a. Figure 4c further characterizes the switching contrast and power penalty at the saturation threshold under varying signal rates. As the signal rate decreases from 40 GBaud to 20 GBaud, the switching contrast monotonically improves by 3.5 dB (8.3 dB to 11.8 dB), while the power penalty is concomitantly reduced by 3.6 dB. This improvement arises from the relaxation of constraints imposed by the finite optical bandwidth of the nanocavity, consistent with the fundamental trade-off between bandwidth and operation speed. Notably, the evaluation of switching speed based on the photon lifetime provides a much more relaxed limit compared to the actual achievable switching speed in practical systems, where the final validation of the switching operation must meet the stringent BER requirements. Whether this performance is acceptable for higher data rates depends on the specific requirements of the application, such as systems with forward-error coding (FEC) techniques. As already demonstrated, increasing the data rate results in a progressive degradation of BER performance, underscoring the importance of balancing speed and error tolerance. Nevertheless, error-free operation has been successfully demonstrated at 40 GBaud based on our design, emphasizing the viability of the proposed design for high-speed signal processing.

## Discussion

**Fig. 5:**
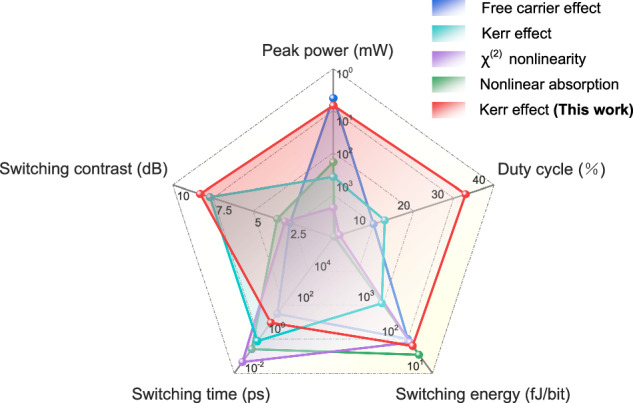
Performance comparison of on-chip all-optical switches based on various nonlinear effects. On-chip all-optical switches based on the free carrier effect^19^, Kerr effect^50^, χ^(2)^ nonlinearity^11^, and nonlinear absorption^20^ are comprehensively compared in terms of peak power, duty cycle, switching energy per bit, switching time, and switching contrast.

In summary, we have demonstrated an on-chip femtojoule optical Kerr switch with milliwatt peak power enabled by an SOH nanocavity. The integration of the PhC nanobeam cavity with the SOH slot waveguide structure containing highly nonlinear polymers significantly enhances optical nonlinearity beyond conventional material platforms while simultaneously achieving a substantial reduction in mode volume. Figure 5 provides a comparative analysis of state-of-the-art integrated all-optical switches based on various nonlinear mechanisms^11,19,22,33^, evaluating five key performance metrics: peak power, duty cycle, switching energy, switching time, and switching contrast. As discussed earlier, an increase in repetition frequency or duty cycle leads to higher average power within a fixed period, which in turn introduces thermal effects or other impairments that can impact device performance in practical applications. It can be seen that compared to existing all-optical switches, our device demonstrates a clear advantage in signal duty cycle and switching contrast, while also exhibiting strong competitiveness in peak power and switching energy. Note that our device also exhibits a compact footprint of only $27\times 1.3\,{{{{\rm{\mu }}}}{{{\rm{m}}}}}^{2}$. These features are primarily attributed to the high Kerr nonlinearity of the polymer and the ultra-small mode volume of the PhC slot nanobeam cavity. Further improvements in device performance can be achieved by minimizing scattering losses at the slot sidewalls through optimized fabrication techniques, such as annealing and thermal oxidation. Additionally, reducing the slot width can enhance the local field confinement and further shrink the mode volume (see Supplementary Information S6 for details). By designing a series of cascaded nanocavities with distinct resonances, optical signal switching can be simultaneously achieved across multiple channels via wavelength-division multiplexing (WDM) techniques, rendering potential for Terabits/s (Tbps) information processing^68^. For such an integrated design, while maintaining the high *Q*-factors and strong cavity-waveguide coupling required for efficient single-cavity switching, the cavity length should be minimized to increase the spectral spacing between resonances, thereby enabling a larger number of parallel channels without spectral overlap. Beyond optical communications, all-optical switching functionality is also highly researched in emerging photonic neural networks, e.g., as an ultra-low-energy all-optical Rectified Linear Unit (ReLU), a key nonlinear activation function frequently employed in deep learning^8^. Our work showcases the strong prospect of an SOH platform for on-chip ultrafast photonic switching technologies, opening new opportunities for applications ranging from communications, computing, to quantum signal processing.

## Methods

### Chip fabrication

The chip is fabricated using an in-house process platform on a 220 nm standard silicon-on-insulator (SOI) wafer. The organic material MEH-PPV, from Sigma Aldrich with an average molecular weight of 70,000–100,000, is a reddish-brown powder. A precise 15 mg of the MEH-PPV powder is weighed using a high-precision electronic balance and transferred into a small vial. Inside an inert atmosphere glove box, 1.6 mL of toluene is added to the vial to dissolve the powder. The resulting solution is then magnetically stirred at 65 °C for 12 h. Finally, the solution is spin-coated onto a SOI wafer to form the cladding layer.

### Spectra characterizations

Spectral characteristics of the fabricated samples are characterized using a broadband light source with an output average power of 7 dBm. The fiber-to-chip coupling loss is estimated to be 6 dB per facet. The SEM images of the grating and corresponding test results are provided in the Supplementary Information S7.

### Experimental setup

High-speed data generation and bit-error rate (BER) measurements are performed using an integrated SHF communication test platform, which includes a synthesized clock generator (SHF 78210 D), a bit-pattern generator (SHF 12104A), and an error analyzer (SHF 11104A). Signal loading is achieved using an RF power amplifier (OA4SMM4) and a Mach–Zehnder modulator (FTM7937EZ). On the receiver side, a photodetector (XPDV2120R) is used. More details are provided in the Supplementary Information S9.

## Supplementary information

Supplementary Information

Transparent Peer Review file

## Data Availability

All the data used for plots generated in this study have been deposited in the database under accession code 10.6084/m9.figshare.31281628.
