# Supplementary material for: Femtojoule optical Kerr switching with milliwatt-peak-power in silicon-organic hybrid nanocavity
Source: Nat Commun. 2026 May 15;17:6483. doi: 10.1038/s41467-026-73285-9 (PMC13376617; doi:10.1038/s41467-026-73285-9)
Supplement: Supplementary file 1 — Supplementary Information [file 41467_2026_73285_MOESM1_ESM.pdf]

Supplementary Information for

Femtojoule Optical Kerr Switching with Milliwatt-Peak-Power in Silicon-Organic Hybrid Nanocavity

Yizheng Chen[1, #], Xiaoyan Gao[1, #], Gaoneng Dong[1], Wentao Gu[1], Jianhua Ning[2], Wentao Ye[2], Yilun Wang[1], Wenchan Dong[1, 3], Lei Lei[2, *], Jing Xu[1, 4, *], Xinliang Zhang[1, 4, *]

[1] Wuhan National Laboratory for Optoelectronics & School of Optical and Electronic Information, Huazhong University of Science and Technology, Wuhan 430074, China

[2] State Key Laboratory of Radio Frequency Heterogeneous Integration (Shenzhen University), Shenzhen, 518060, China

[3] Hubei Optical Fundamental Research Center, Wuhan, China

[4] Optics Valley Laboratory, Hubei 430074, China

[#] These authors contributed equally: Yizheng Chen, Xiaoyan Gao

[*] Corresponding author: leilei@szu.edu.cn; jing_xu@hust.edu.cn; xlzhang@mail.hust.edu.cn;

Contents

S1. Parameter Comparison of Material Platforms 2

S2. Theoretical Models 4

S3. The Impact of Silicon-Related Nonlinear Absorption in Nanocavity 9

S4. Dynamic Switching Characteristics 12

S5. Design Guideline 15

S6. Effective Nonlinear Coefficient 18

S7. Fabrication and Devices Details 20

S8. Thermal Effects of Polymers 24

S9. Experimental Setup for Optical Measurement 29

S10. Femtojoule 30 GBaud Switching Results 30

S11. Long-term Stability Measurements 32

S12. Performance Comparison of On-chip All-optical Switches 33

Supplementary References 34

S1. Parameter Comparison of Material Platforms

Figure **S1** summarizes key material parameters of representative integrated photonic platforms, including the linear refractive index, nonlinear refractive index (n_2), and bandgap energy. In general, the relationship between the linear and the nonlinear refractive index approximately follows Miller's rule for most materials, i.e., the nonlinear refractive index increasing exponentially with the linear refractive index. However, this enhancement of Kerr nonlinearity is intrinsically accompanied by a reduction in bandgap energy, leading to pronounced two-photon absorption (TPA) and subsequent free-carrier absorption (FCA) at telecommunication wavelengths. As a result, material selection for Kerr-based switching is fundamentally governed by a balance between nonlinear strength and nonlinear absorption.

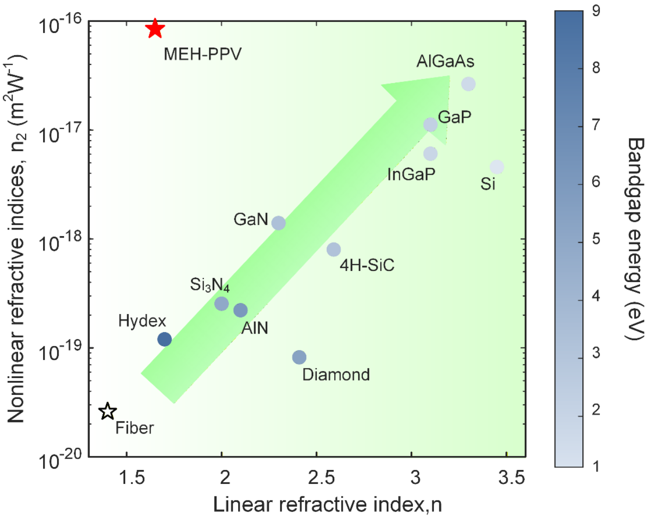

Fig. S1. Nonlinear refractive indices (n_2), bandgap energy, and linear refractive indices (n) of different material platforms.

Silicon and silicon nitride represent two typical examples of this trade-off. Silicon exhibits a relatively large nonlinear refractive index ($n_2 = 4.5 \times 10^{-18}$ m^2/W) and strong optical confinement owing to its high refractive index, enabling compact devices with enhanced Kerr interaction. Nevertheless, its practical performance is severely constrained by TPA and FCA under high-intensity operation. In contrast, silicon nitride features a wide bandgap and negligible TPA at 1550 nm, but its significantly smaller nonlinear refractive index ($n_2 = 2.5 \times 10^{-19}$ m^2/W, approximately 18 times

lower than silicon) necessitates centimetre- to meter-scale waveguides or ultra-high-Q resonators to accumulate sufficient nonlinear phase shift. Such approaches, however, are generally incompatible with low-power and high-speed (>10–40 GBaud) all-optical signal processing due to footprint, bandwidth, and energy-efficiency limitations. Notably, organic nonlinear materials (MEH-PPV) deviate from the conventional Miller scaling. Despite their relatively low linear refractive indices, they can exhibit exceptionally large Kerr nonlinearities ($n_2 = 8.5 \times 10^{-17}$ m^2/W, approximately 20 times that of silicon), while maintaining negligible nonlinear absorption in the telecommunication band. This unique combination enables a fundamentally different balance between nonlinearity and loss compared to inorganic materials.

By integrating high-nonlinear organic materials with silicon in a silicon-organic hybrid (SOH) configuration, the resulting platform simultaneously benefits from the strong optical confinement enabled by the high refractive index of silicon and the large, low-loss Kerr response of the organic polymer. It is important to note that the refractive index of slot structure play a critical role in the mode confinement of the switches. Figure **S2**(a) and (b) compares the mode confinement between two slot structures made of silicon and silicon nitride, respectively. It is clear that compared to silicon nitride, silicon slot geometry enables stronger concentrations of the optical field within the polymer region, substantially allowing more pronounced Kerr nonlinearity. This structure also elegantly relieves the impact of TPA and FCA in silicon since the mode intensity in the silicon region is small. Therefore, the SOH platforms effectively balance nonlinearity, absorption, and optical confinement—an intrinsic trade-off that constrains Kerr-effect-based switching in conventional material systems.

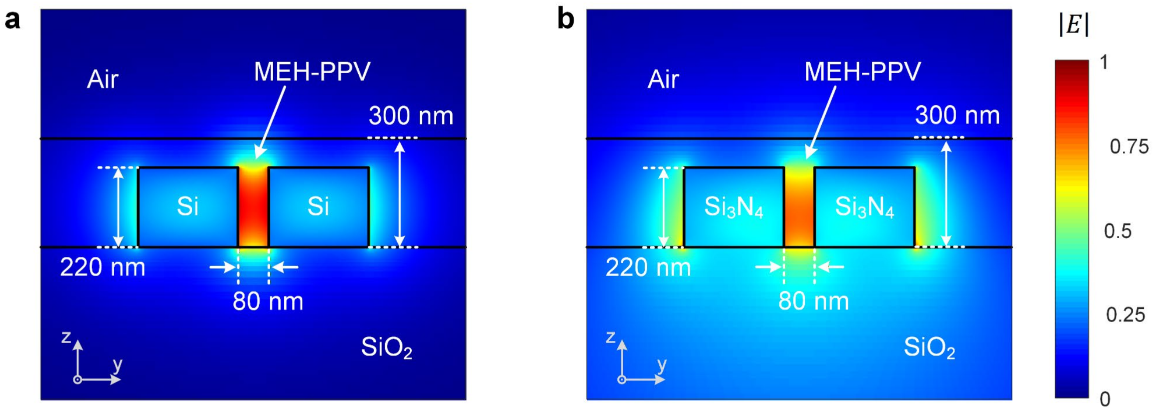

Fig. S2. Calculated electric field distributions ($|E|$) of silicon-organic hybrid integration (a) and silicon nitride-organic hybrid integration (b).

S2. Theoretical Models

We present the theoretical framework for modeling the coupled structure of the high-Q photonic crystal (PhC) slot nanobeam cavity and the low-Q F-P cavity shown in Fig. **1a**, utilizing a combination of temporal coupled mode theory (TCMT) [1] and the transfer matrix method (TMM) [2]. In the equivalent simplified model shown in Fig. **S3**, the two air holes in the bus waveguide are treated as two independent reflective regions, with r and t representing the reflection and transmission coefficients, respectively, subject to the condition $r^2 + t^2 = 1$. We define the mode field inside the resonator as a, corresponding to the resonance frequency ω_0. The coupling coefficient between the resonator and the waveguide is denoted as κ, which satisfies the relationship $\kappa = \sqrt{\gamma_c/2}$, where γ_c is the coupling rate. The intrinsic decay rate of the resonator is defined as γ_0. Furthermore, the intrinsic decay rate γ_0 and coupling rate γ_c are related to the cavity intrinsic and external coupling Q-factors (Q_i and Q_e), by the equations $\gamma_0 = \omega_0/Q_i$ and $\gamma_c = \omega_0/Q_e$. The input field is represented by S_{1+}, the output field by S_{2-}, and the input field at the other end is set to $S_{2+} = 0$.

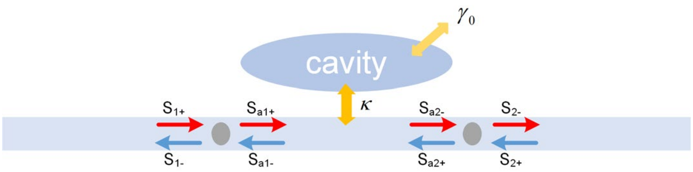

Fig. S3. Schematic diagram of equivalent and simplified equipment model.

The entire process can be initially described using the widely adopted TMM:

$$\begin{bmatrix} S_{2-} \\ S_{2+} \end{bmatrix} = T_1 T_2 T_3 T_2 T_1 \begin{bmatrix} S_{1+} \\ S_{1-} \end{bmatrix} \tag{S1}$$

The sub-transmission matrices, T_1, T_2, and T_3, represent the transmission at three distinct locations: T_1 describes the transmission through a single air hole, T_2 corresponds to the transmission along the waveguide from one of the air holes to the center, and T_3 accounts for the coupling between the waveguide and the photonic crystal (PhC) slot nanobeam cavity, derived from the steady-state solution of the TCMT. The detailed expressions for these matrices are provided below:

$$T_1 = \frac{1}{it} \begin{bmatrix} -(r^2 + t^2) & -r \\ r & 1 \end{bmatrix} \tag{S2}$$

$$T_2 = \begin{bmatrix} e^{i\delta} & 0 \\ 0 & e^{-i\delta} \end{bmatrix} \tag{S3}$$

$$T_3 = \begin{bmatrix} 1 - \dfrac{i\gamma_c/2}{\Delta\omega + i\gamma_0/2} & \dfrac{-i\gamma_c/2}{\Delta\omega + i\gamma_0/2} \\ \dfrac{i\gamma_c/2}{\Delta\omega + i\gamma_0/2} & 1 + \dfrac{i\gamma_c/2}{\Delta\omega + i\gamma_0/2} \end{bmatrix} \tag{S4}$$

where $\delta = \omega n_{\text{eff}} L_{\text{FP}}/2c$ is the phase shift of light in the F-P cavity from one of the air holes to the center, L_{FP} is the length of the F-P cavity, n_{eff} is the effective refractive index in the waveguide, and c is the speed of light in vacuum. $\Delta\omega = \omega - \omega_0$ denotes the frequency detuning, where ω is the frequency of the input light and ω_0 the resonance frequency. By substituting the Eq. (S2) to (S4) into Eq. (S1), the transmission spectrum of the structure can be expressed as follows:

$$T = \left|\frac{S_{2-}}{S_{1+}}\right|^2 = \left|\frac{(\Delta\omega + i\gamma_0/2)t^2 e^{2i\delta}}{(\Delta\omega + i\gamma_0/2 - i\gamma_c/2)r^2 e^{4i\delta} + i\gamma_c r e^{2i\delta} - \Delta\omega - i\gamma_0/2 - i\gamma_c/2}\right|^2 \tag{S5}$$

According to the previous study [3], adjusting the cavity length (L_{FP}) of the F-P resonator allows for effective tuning of the Fano line shape. In addition, the experimentally measured transmission spectrum exhibits small periodic ripples with a characteristic period of approximately 1.4 nm originating from residual F-P interference due to weak reflections at the input/output facets and air-hole interfaces. This effect can be incorporated as a slowly varying multiplicative modulation term in the overall transmission function, $T_{exp} = T \times [1 + \alpha_{\text{FP}} T_{\text{FP}}]$ (where T refers to Eq. (S5), α_{FP} denotes the small modulation depth and T_{FP} represents a normalized periodic interference function). The extinction ratio (ER) is defined as the transmission contrast between the peak and the dip in the spectrum. To simplify the analysis, two approximations are introduced. First, the internal loss can be considered negligible compared to the coupling loss, as high-Q PhC cavity typically exhibits intrinsic Q-factor far exceeding the external coupling Q-factor ($Q_i \gg Q_e$), corresponding to a negligible internal decay rate ($\gamma_0 \ll \gamma_c$). Second, the minimum transmission is approximated to occur at the resonance frequency, such that $T_{min} \approx T(\omega_0)$, which serves as a valid simplification for analyzing the transmission spectrum. Consequently, the expression for the ER is given as follows:

$$ER = 10\log\left(\frac{T_{\max}}{T_{\min}}\right) \approx 10\log\left(\left|\frac{re^{2i\delta} - 1}{2(re^{2i\delta} + 1) - i(re^{2i\delta} - 1)^2}\right|^2 \cdot (4{\gamma_c}^2/{\gamma_0}^2 + 1)\right) \tag{S6}$$

To further simplify the expressions, $\beta = \left|\frac{re^{2i\delta}-1}{2(re^{2i\delta}+1)-i(re^{2i\delta}-1)^2}\right|^2$ is introduced as a substitution:

$$ER = 10\log\left(4\beta\left(\frac{Q_i}{Q_l}-1\right)^2+\beta\right) \quad \text{(S7)}$$

Moreover, as evident from Eq. (S7), for a fixed Q_l, increasing the intrinsic Q-factor (Q_i) of the nanobeam cavity significantly enhances the ER of the Fano resonance. This improvement is crucial for achieving high-contrast all-optical switching.

Next, to represent the nonlinear processes in the nanocavity, we employ the nonlinear TCMT to describe the dynamic behavior of the switches. The coupled mode equations for the pump and probe lights within the cavity are given by the following expression, where m represents either the pump ($m = p$) or probe light ($m = s$):

$$\frac{\mathrm{d}a^m}{\mathrm{d}t} = \left(i(\omega'_{m0}-\omega_0^m)-\frac{\gamma_0}{2}-\frac{\gamma_c}{2}\right)a^m + \kappa S_{a1+}^m e^{-i\delta} + \kappa S_{a2+}^m e^{-i\delta} \quad \text{(S8)}$$

ω'_{m0} denotes the resonant frequency of the nanocavity after the drift caused by the Kerr effect, which can be expressed as follows:

$$\omega'_{m0} = \frac{2\pi c}{\lambda_0^m + \Delta\lambda_{\mathrm{Kerr}}^m} \quad \text{(S9)}$$

where $\Delta\lambda_{\mathrm{Kerr}}^m$ represents the corresponding shift in the resonant wavelength,

$$\Delta\lambda_{\mathrm{Kerr}}^s = \frac{2n_2 c\lambda_0^s}{n_0^2 V^s} C_{Overlap}\left|a_p\right|^2 \quad \text{(S10)}$$

$$\Delta\lambda_{\mathrm{Kerr}}^p = \frac{n_2 c\lambda_0^p}{n_0^2 V^p}\left|a_p\right|^2 \quad \text{(S11)}$$

n_2 denotes the nonlinear refractive index of MEH-PPV, c is the speed of light in vacuum, λ_0^m represents the initial resonant wavelength of the nanocavity, n_0 refers to the initial refractive index of MEH-PPV, V^mcorresponds to the mode volume of the nanocavity, and $C_{Overlap}$ is the electric field overlap integral of the two resonant modes. Since the pump light power is significantly higher than that of the probe light, the nonlinear effects induced by the probe light can be neglected. As a result, the factor of two difference between Eq. (S10) and Eq. (S11) arises from the distinction between the cross-phase modulation (XPM) and self-phase modulation (SPM) processes. Moreover, because the pump and probe lights correspond to different resonant modes, their interaction strength is

proportional to the electric field overlap integral ($C_{Overlap}$) of the two modes, which can be expressed as follows:

$$C_{Overlap} = \frac{|\int E_1^*(r) \cdot E_2(r) \cdot \varepsilon(r) dr|^2}{(\int |E_1(r)|^2 \cdot \varepsilon(r) dr)(\int |E_2(r)|^2 \cdot \varepsilon(r) dr)} \tag{S12}$$

where E_1 and E_2 are the spatial electric field distributions of the two modes respectively, and ε is the dielectric constant of the material [4]. Additionally, since the optical field must propagate through the F-P cavity within the bus waveguide, the scattering matrix for the air holes at both ends of the cavity and the transmission equations between them are given as follows:

$$S_{a1-}^m = S_{a2+}^m e^{-2i\delta} - \kappa a^m e^{-i\delta} \tag{S13}$$

$$S_{a2-}^m = S_{a1+}^m e^{-2i\delta} - \kappa a^m e^{-i\delta} \tag{S14}$$

$$\begin{bmatrix} S_{a1+}^m \\ S_{a1-}^m \end{bmatrix} = \frac{1}{it} \begin{bmatrix} -(r^2 + t^2) & -r \\ r & 1 \end{bmatrix} \begin{bmatrix} S_{1+}^m \\ S_{1-}^m \end{bmatrix} \tag{S15}$$

$$\begin{bmatrix} S_{a2+}^m \\ S_{a2-}^m \end{bmatrix} = \frac{1}{it} \begin{bmatrix} -(r^2 + t^2) & -r \\ r & 1 \end{bmatrix} \begin{bmatrix} S_{2+}^m \\ S_{2-}^m \end{bmatrix} \tag{S16}$$

To better analyze the switching operation, the dynamic characteristics of the intracavity pump and probe light fields are derived from Eq. (S8) to (S16), and the resulting output light fields S_{2-}^m, are expressed as follows:

$$S_{a1+}^m e^{-i\delta} = \frac{ite^{-i\delta}}{1 - r^2 e^{-4i\delta}} S_{1+}^m + \frac{\kappa\left(re^{-2i\delta} - r^2 e^{-4i\delta}\right)}{1 - r^2 e^{-4i\delta}} a^m = \sigma_1 S_{1+}^m + \kappa\sigma_2 a^m \tag{S17}$$

$$S_{a2+}^m e^{-i\delta} = \frac{-irte^{-3i\delta}}{1 - r^2 e^{-4i\delta}} S_{1+}^m + \frac{\kappa\left(re^{-2i\delta} - r^2 e^{-4i\delta}\right)}{1 - r^2 e^{-4i\delta}} a^m = \sigma_3 S_{1+}^m + \kappa\sigma_2 a^m \tag{S18}$$

$$S_{2-}^m = -\frac{it}{r} S_{a2+}^m \tag{S19}$$

where σ_1, σ_2 and σ_3 are coefficient related to the reflection coefficient and cavity length of the low-Q F-P cavity. To solve the above nonlinear temporal coupled mode equations accurately, a differential iterative algorithm is necessary. To preliminarily assess the factors influencing the switching energy, an estimation is performed using the steady-state solution. Assuming the pump light is tuned to the initial resonant frequency of the cavity (ω_0^p) with a small detuning, i.e., $\omega = \omega_0^p$, the stored energy in the cavity, $|a^p|^2$, is given by:

$$|a^p|^2 = \left| \frac{\sqrt{\frac{\gamma_c}{2}}(\sigma_1 + \sigma_3)}{\frac{\gamma_0}{2} - \frac{\gamma_c}{2}(2\sigma_2 - 1)} \right|^2 |S_{1+}^p|^2 = \left| \frac{(\sigma_1 + \sigma_3)\sqrt{\frac{2}{\omega_0}}}{\frac{\sqrt{Q_e}}{Q_l} - 2\sigma_2\sqrt{\frac{1}{Q_e}}} \right|^2 |S_{1+}^p|^2 \tag{S20}$$

The field enhancement factor FE inside the nanocavity is given by $FE \propto \left|a^p/S_{1+}^p\right|^2$ [5]. A larger FE indicates stronger optical field localization and a higher energy density within the cavity. In nonlinear processes, a high FE can significantly lower the input power threshold required to induce nonlinear effects. As evident from Eq. (S20), for a fixed Q_l, a larger Q_i leads to a smaller Q_e due to the relation $1/Q_l = 1/Q_i + 1/Q_e$, resulting in a higher enhancement factor (FE). Since $Q_i \gg Q_e \approx Q_l$, Eq. (S20) can be further simplified as:

$$|a^p|^2 \approx \frac{2Q_l^p}{\omega_0^p}\left|\frac{\sigma_1 + \sigma_3}{1 - 2\sigma_2}\right|^2 \left|S_{1+}^p\right|^2 \tag{S21}$$

Next, assuming that the switching of the probe light transmission from minimum to maximum constitutes a complete switching process (i.e., $\Delta\lambda_{\mathrm{Kerr}}^s \approx \lambda_0^s/Q_l^s$), the required peak power, $E_{peak\_power} = \left|S_{1+}^p\right|^2$, can be expressed as follows:

$$E_{peak\_power} = \left|S_{1+}^p\right|^2 = \frac{\sigma V_{\mathrm{Kerr}}^s}{n_2 \cdot Q_l^s \cdot Q_l^p \cdot C_{Overlap}} \tag{S22}$$

where Q_l^s and Q_l^p represent the loaded Q-factors of the Fano mode 1 and Fano mode 2, respectively. The coefficient σ is related to the reflection coefficient and cavity length of the low-Q F-P cavity and is given by:

$$\sigma = \frac{\pi n_0^2}{2\lambda_0^p}\left|\frac{1 - 2\sigma_2}{\sigma_1 + \sigma_3}\right|^2 \tag{S23}$$

Furthermore, the required switching energy is given by $U_{in} = E_{peak\_power}\delta/R_b$, where R_b denotes the signal bit rate, and δ represents the duty cycle. According to the Eq. (S22), the switching energy of a Kerr all-optical switch can be effectively reduced by using nonlinear material with higher n_2 and cavity with smaller mode volume. Increasing Q-factor of the cavity and improving the mode overlap also play important roles.

S3. The Impact of Silicon-Related Nonlinear Absorption in Nanocavity

Although the nonlinear response of the present device is dominated by the polymer-filled slot, the optical mode inevitably exhibits a finite overlap with the surrounding silicon rails of the PhC slot nanobeam cavity. As a result, silicon-related nonlinear processes, including two-photon absorption (TPA), free-carrier absorption (FCA), and free-carrier dispersion (FCD), may in principle contribute to the overall switching dynamics and therefore warrant careful evaluation. To examine the contribution of silicon-related nonlinearities, we analyze the modal energy distribution of both Fano resonances (as shown in the inset of Fig. **1a** in the main text) by integrating the simulated electric-field energy density over the polymer-filled slot and the silicon regions, respectively. This analysis shows that approximately 70% of the optical energy is confined within the polymer-filled slot, while the remaining ~30% resides in the silicon rails. Despite this non-negligible fraction of modal energy in silicon, the effective mode area associated with the silicon rails is substantially larger than that of the narrow slot. As a result, the optical intensity in the polymer-filled slot, evaluated at the central cross-section of the nanocavity, is approximately eight times higher than that in the surrounding silicon rails. Since TPA, FCA, and FCD scale with optical intensity rather than stored energy alone, the reduced intensity strongly suppresses silicon-related nonlinear effects under the operating conditions considered here.

To rigorously account for these residual effects, we extend the TCMT framework introduced in Section **S2** to explicitly include contributions from TPA, FCA, and FCD. First, we establish the relationship between the input pump power and the intracavity field, from which the corresponding nonlinear absorption and carrier-induced effects are derived. Specifically, the governing equations presented below are derived by extending Eq. (S8) to include additional contributions from TPA-, FCA-, and FCD-related processes. Because the pump power is substantially higher than that of the probe, only the pump-induced carrier generation and associated nonlinear effects are considered in this analysis, while probe-induced carrier effects are neglected:

$$\frac{\mathrm{d}a^p}{\mathrm{d}t} = \left(i\Delta\omega - \frac{\gamma_0}{2} - \frac{\gamma_c}{2} - i\delta_{FC} - \frac{\gamma_{TPA}}{2} - \frac{\gamma_{FCA}}{2}\right)a^p + \kappa S_{a1+}^{p} e^{-i\delta} + \kappa S_{a2+}^{p} e^{-i\delta} \quad \text{(S24)}$$

Here, δ_{FC} denotes the frequency shift induced by FCD, while γ_{TPA} and γ_{FCA} represent the

additional cavity decay rates arising from TPA and FCA, respectively. These decay rates are related to the corresponding optical absorption coefficients α_{TPA} and α_{FCA} through $\gamma_{\text{TPA,FCA}} = (c/n_{\text{eff}}^{\text{cav.}}) \cdot \alpha_{\text{TPA,FCA}}$ (c is the speed of light in vacuum and $n_{\text{eff}}^{cav.}$ is the effective refractive index in the nanocavity). In the nanocavity, the TPA-induced absorption depends on the intracavity pump intensity. Accordingly, the TPA-related absorption coefficient can be written as:

$$\alpha_{\text{TPA}} = \frac{\beta_{\text{TPA}} c p_{\text{Si}}}{n_{\text{Si}} V_{\text{TPA}}} |a^p|^2 \quad (S25)$$

Where β_{TPA} is the two-photon absorption coefficient of silicon ($\beta_{\text{TPA}} \approx 8 \times 10^{-12}\ \text{m} \cdot \text{W}^{-1}$), n_{Si} is the refractive index of silicon, V_{TPA} is the effective mode volume associated with the TPA process, and p_{Si} denotes the fraction of the total intracavity optical energy residing in the silicon rails of the nanocavity ($p_{\text{Si}} \approx 0.3$).

The FCA-induced absorption depends on the carrier density inside the cavity. The free-carrier density $N(t)$ generated by TPA satisfies the carrier rate equation:

$$\frac{\text{d}N(t)}{\text{d}t} = \frac{\beta_{\text{TPA}} c^2 {p_{\text{Si}}}^2}{2\hbar\omega_0^p {n_{\text{Si}}}^2 {V_{\text{FCA}}}^2} |a^p|^4 - \frac{N(t)}{\tau_{recom}} \quad (S26)$$

where $\hbar$ is the reduced Planck constant ($\hbar = h/2\pi$), V_{FCA} is the effective mode volume associated with FCA, and τ_{recom} is the effective free-carrier lifetime in silicon. For a material containing free carriers, the optical response can be described by the Drude model. The complex permittivity ε' of silicon in the presence of free carriers is given by:

$$\varepsilon' = \varepsilon_1 + j\varepsilon_2 = \varepsilon_0 - \frac{\omega_{plasma}^2}{\left(\omega_0^p\right)^2} + j\frac{\omega_{plasma}^2}{\left(\omega_0^p\right)^3 \tau_{relax}} \quad (S27)$$

where ε_0 is the intrinsic permittivity of silicon, τ_{relax} is the carrier relaxation time (approximately 0.17 ps for electrons and 0.1 ps for holes), and ω_{plasma} is the plasma frequency. The plasma frequency is related to the free-carrier density $N(t)$ by:

$$\omega_{plasma}^2 = \frac{e^2 N(t)}{\varepsilon_0 m^*} \quad (S28)$$

where e is the elementary charge, and m^* is the effective carrier mass. As a result, the FCA-induced absorption coefficient can be expressed as:

$$\alpha_{\text{FCA}} = \frac{\omega_0^p \varepsilon_2}{c n_{Si}} \quad (S29)$$

where ε_2 denotes the imaginary part of the complex permittivity ε'.

By solving Eq. (S24) to (S29) under resonant conditions, we obtain the relationship between the

intracavity power and the input pump power, explicitly accounting for nonlinear absorption effects, as shown in Fig. **S4**. The red dashed line represents the intracavity power calculated without nonlinear absorption, whereas the blue solid curve includes the contributions from carrier-related losses. The yellow curve indicates the corresponding nonlinear loss. As the intracavity power increases, nonlinear losses rise progressively, leading to a reduction in the cavity power enhancement factor and ultimately slowing the growth rate of intracavity power. However, within the on-chip pump power range employed in the experiments—corresponding to an average on-chip power below 3 dBm, as indicated by the gray dashed line in Fig. **S4**—the free-carrier-induced loss and phase shift in silicon remain negligibly small and do not produce any measurable impact on the switching dynamics. Consequently, carrier-related effects in silicon can be safely neglected under the operating conditions of this work.

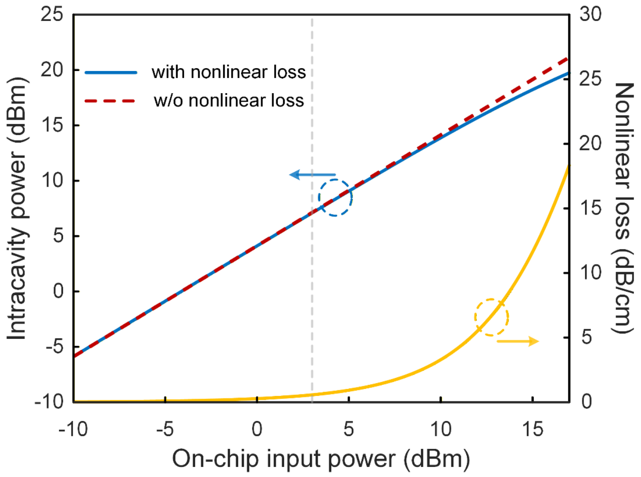

Fig. S4. The impact of silicon-related nonlinear absorption in nanocavity.

S4. Dynamic Switching Characteristics

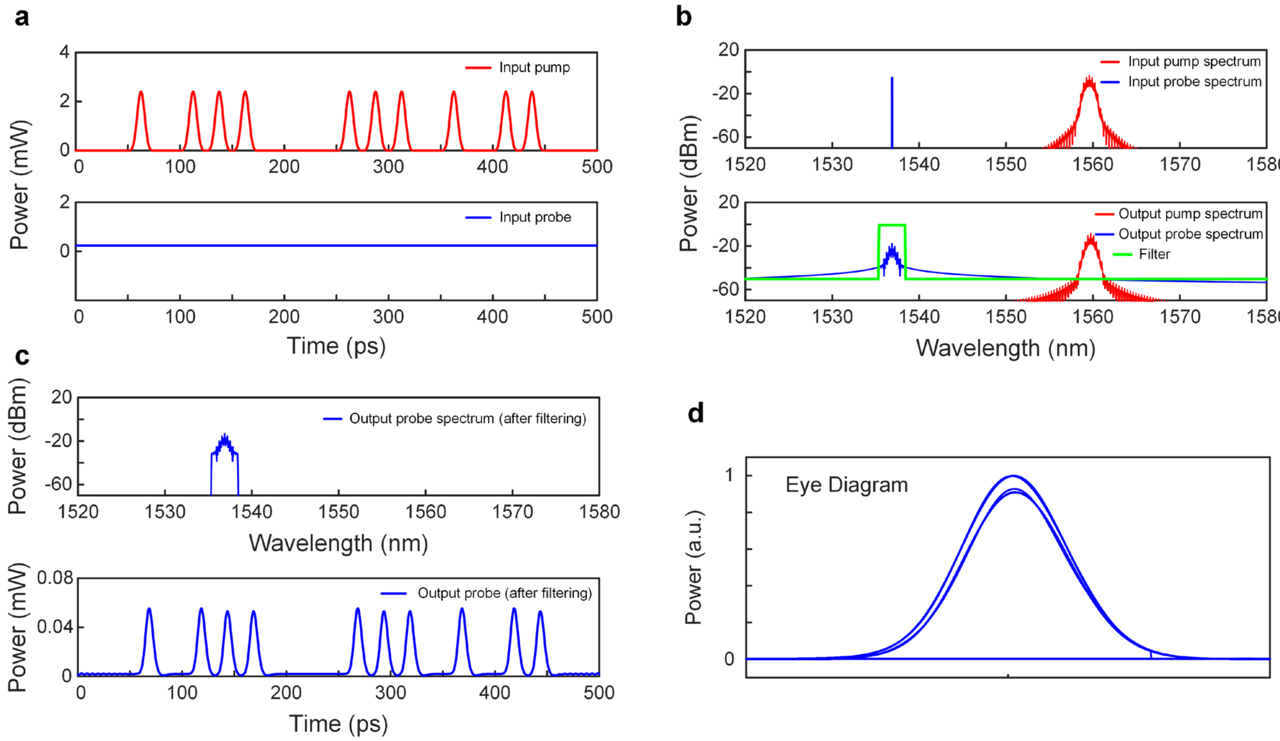

Fig. S5. Dynamic characteristics of the 40 Gbit/s switching. a. Time-domain waveforms of the input pump light and probe light. **b.** Input and output light spectrum. **c.** Filtered output spectrum and corresponding time-domain waveform of the probe light. **d.** Eye diagram of the output signal.

Based on the nonlinear time-domain coupled-mode theoretical model presented in Section **S2**, we simulate the dynamic switching characteristics of the Kerr all-optical switch using a differential iterative algorithm. The simulation adopts the same resonance conditions as in Fig. **2(c)** and **(d)**. The Q_l, Q_i, and resonant wavelengths for Fano mode 1 are 1.8×10^3, 1.3×10^4, and 1536.89 nm, respectively, while those for Fano mode 2 are 2.5×10^3, 4.5×10^3, and 1559.13 nm, respectively. At the input side (Fig. **S5a**), the pump light consists of 40 Gbit/s RZ-OOK signals with a 33% duty cycle and a peak power of 3.5 mW, corresponding to a switching energy of 29 fJ/bit, while the probe light is continuous. Figure **S5b** shows the input and output spectrum, where an optical bandpass filter is applied to isolate the probe light. The filtered output spectrum and the corresponding time-domain waveform of the recovered probe light are presented in Fig. **S5c**, exhibiting a switching contrast of 11.9 dB. During the

"1" bit of the pump light, the probe light switches on in response. As illustrated in the eye diagram of Fig. **S5d**, the degradation of the output probe signal, caused by optical bandwidth limitations, results in a thickening of the upper eye opening.

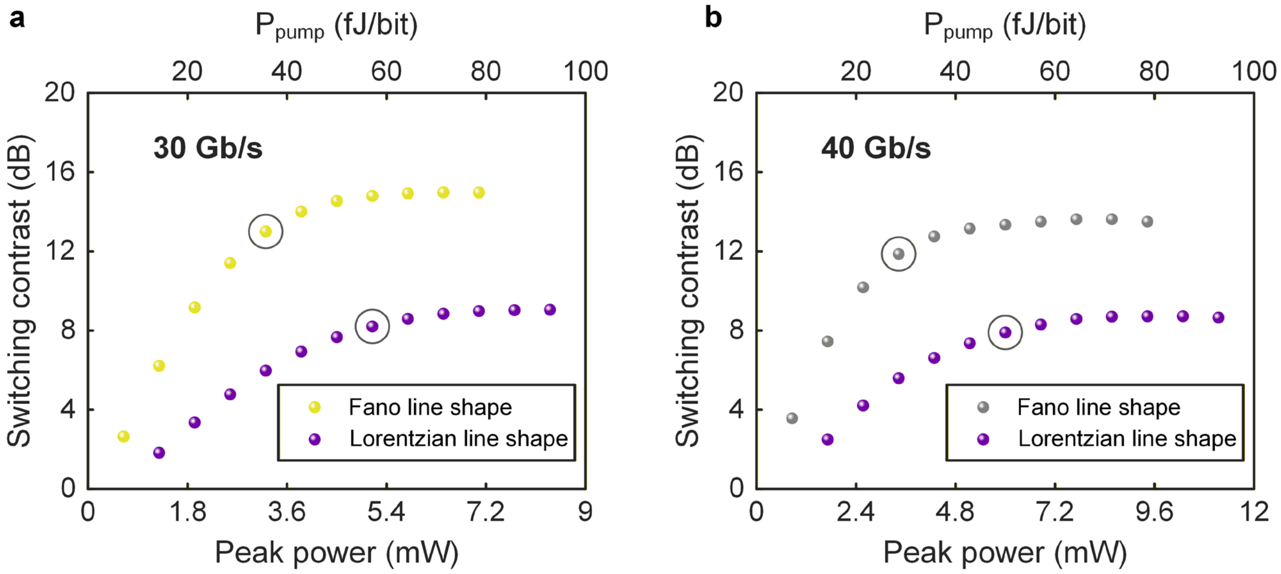

Fig. S6. Switching performance at signal rates of 30 Gbit/s (a) and 40 Gbit/s (b). a. Switching performance based on Fano (yellow dots) and Lorentzian (purple dots) line shapes at a signal rate of 30 Gbit/s. **b.** Switching performance based on Fano (grey dots) and Lorentzian (purple dots) line shapes at a signal rate of 40 Gbit/s.

Fano resonances arise from the interference between a discrete state and a continuum of states. In systems where two resonant cavities with different Q-factors are coupled, the interference leads to sharp asymmetric features in the transmission spectrum, known as Fano line shapes [6]. Here, we quantitatively assess the dynamic switching characteristics of a Fano-resonant structure and compare its performance with that of a Lorentzian-resonant structure, both having identical Q_i and Q_e, as shown in Fig. **S6**. The results show that the switching contrast at both 30 Gbit/s (Fig. **S6a**) and 40 Gbit/s (Fig. **S6b**) signal rates increases with rising switching energy and eventually saturates, consistent with experimental trends. At 30 Gbit/s, the switching energy for the Fano line shape is approximately 36 fJ/bit, yielding a switching contrast of 13 dB, whereas the Lorentzian line shape requires about 57 fJ/bit with a switching contrast of 8.2 dB. When the signal rate increases to 40 Gbit/s, the switching energy for the Fano line shape is approximately 29 fJ/bit, achieving a switching contrast of 11.9 dB, while the Lorentzian line shape exhibits a switching energy of roughly 50 fJ/bit with a

switching contrast of 7.9 dB. It is evident that the all-optical switch based on the Fano line shape demonstrates lower switching energy and higher switching contrast, which can be primarily attributed to the steep edges and high extinction ratio of the Fano transmission spectrum.

S5. Design Guideline

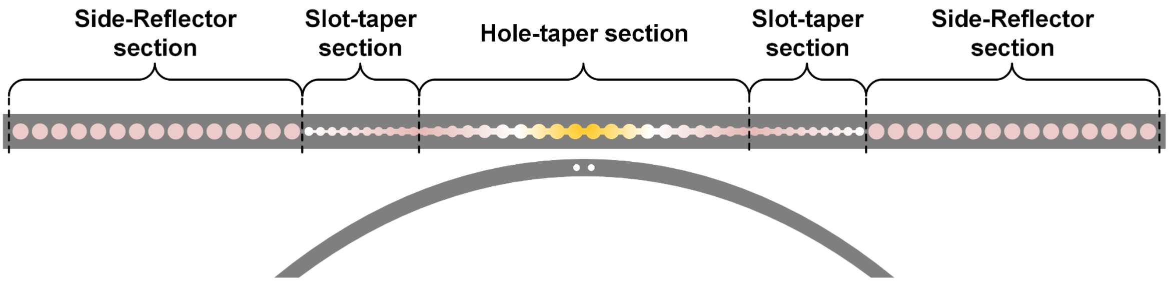

Fig. S7. Schematic structure of the all-optical switch based on the PhC slot nanobeam cavity.

Equations (S7) and (S20) provide estimations of the extinction ratio (ER) and the field enhancement factor (FE) inside the nanocavity, respectively. These relationships indicate that increasing the Q_i of the nanobeam cavity significantly enhances the contrast of the all-optical switch while reducing the required switching energy. However, selecting an appropriate Q_e is equally critical for achieving high switching speeds. While a higher Q_l may restrict the switching speed, a lower Q_l can significantly increase the switching energy. Therefore, a trade-off between switching energy and speed must be carefully considered to optimize the Q-factor for the device.

Figure **S7** illustrates the design of the all-optical switch based on the PhC slot nanobeam cavity. The structure consists of three distinct sections: hole-taper section, slot-taper section, and side-reflector section. In the hole-taper section, the slot width is maintained at 80 nm, while the air hole radius gradually decreases from 130 nm at the center to 120 nm at the edges following a parabolic gradient. In the slot-taper section, the air hole radius remains constant at 116 nm, whereas the slot width symmetrically reduces from 80 nm to 40 nm towards both ends. Two tapered sections are employed to ensure a smooth transition of the light field and form a Gaussian mirror to optimize the light confinement [7]. In the outermost side-reflector section, the air holes have a radius of 156 nm without the slot in order to enhance the confinement of the optical field, ensuring high intrinsic Q-factor of the nanocavity. The lattice constant in all sections is maintained at 386 nm. This integrated design, combining tapered transitions and optimized hole geometries, effectively suppresses mode mismatch losses and significantly enhances the performance of the all-optical switch.

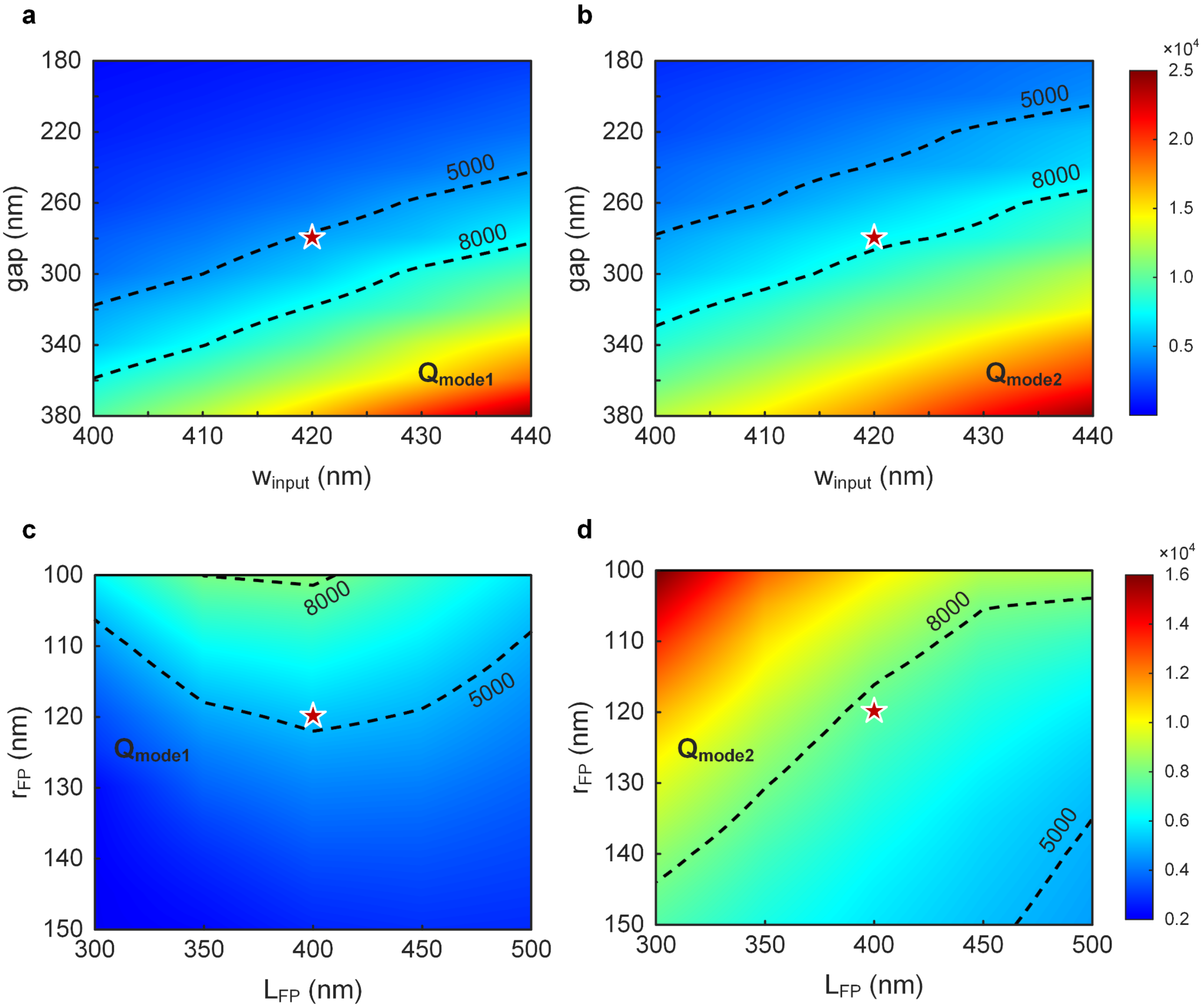

Fig. S8. Design Guidelines for Coupling Rate between High-Q Slot PhC Cavity and Low-Q F-P Cavity. a. b. The colormap shows the loaded Q-factors of the Fano mode 1 (**a**) and Fano mode 2 (**b**) as functions of the bus waveguide width (w_{input}) and the distance to the PhC slot nanobeam cavity (gap). **c. d.** The colormap shows the loaded Q-factors of the Fano mode 1 (**c**) and Fano mode 2 (**d**) as functions of the radius (r_{FP}) and distance (L_{FP}) of the two air holes in the bus waveguide. The black dashed box indicates the design Q-factor range, while the red pentagram marks the final selected parameters: $w_{input} = 420\ nm,\ gap = 280\ nm,\ r_{\mathrm{FP}} = 120\ nm,\ L_{\mathrm{FP}} = 400\ nm.$

As mentioned in the main text, achieving a switching speed of 40 GBaud necessitates optimizing the coupling rate between the high-Q slot PhC cavity and the low-Q F-P cavity. Figure **S8** illustrates the dependence of the Q_l on key structural parameters, including the bus waveguide width (w_{input}), the coupling distance to the cavity (gap), and the air hole size (r_{FP}) and spacing (L_{FP}) in the low-Q F-

P cavity. To ensure manufacturability, the Q_l is constrained within the range of 5000 and 8000, as indicated by the black dashed line in Fig. **S8**. Since both Fano mode 1 and Fano mode 2 participate in the switching process, it is crucial to select parameters that simultaneously satisfy the Q-factor requirements for both modes. After systematically balancing coupling efficiency and fabrication tolerances, the optimized design parameters are as follows: a bus waveguide width of 420 nm, a coupling distance of 280 nm to the PhC slot nanobeam cavity, and air hole radius and spacing of 120 nm and 400 nm, respectively. The final Q_{loaded} for Fano mode 1 and Fano mode 2 are 5.3×10^3 and 7.0×10^3, respectively. These choices ensure an optimal coupling rate, enhancing switching efficiency and supporting the 40 GBaud switching speed target. The corresponding electric field distributions ($|E|$) are shown in Fig. **S9**.

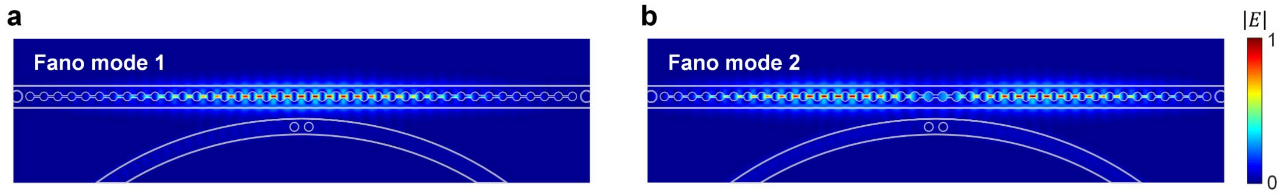

Fig. S9. Calculated electric field distributions ($|E|$) of Fano mode 1 (a) and Fano mode 2 (b).

S6. Effective Nonlinear Coefficient

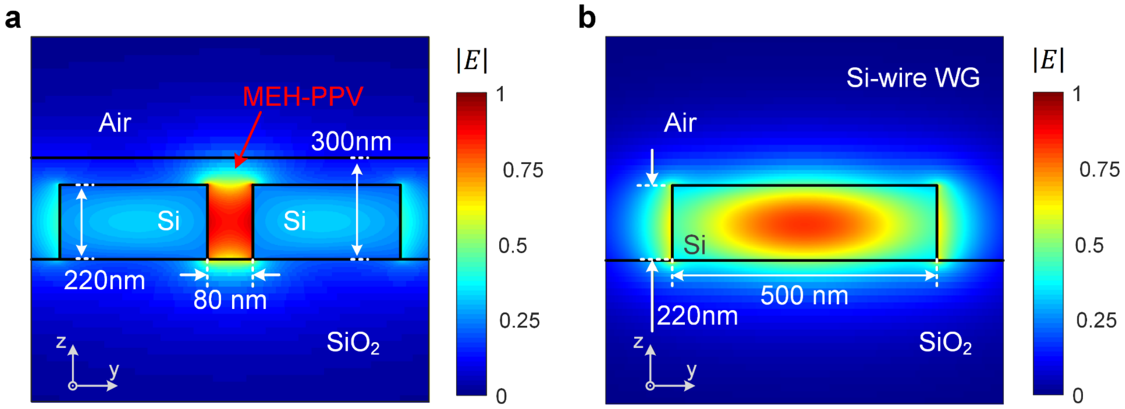

Fig. S10. Calculated electric field distributions (|E|) of the central cross-section of the PhC slot nanobeam cavity (a) and the standard Si-wire WG (b). a. The slot width at the center of the PhC slot nanobeam cavity is 80 nm, the waveguide width is 600 nm, and the MEH-PPV thickness is 300 nm. **b.** The width of the standard Si-wire WG is 500 nm.

The effective nonlinear coefficient (γ_{eff}), which quantifies the strength of nonlinear optical interactions from a wave-guiding perspective, is widely adopted in nonlinear photonic analysis [8]. To evaluate the enhancement provided by the slot PhC nanocavity, we estimate its effective nonlinear coefficient and compare it with that of a standard silicon wire waveguide (Si-wire WG). Figure **S10a** presents the electric field distribution in the central cross-section (yz-plane) of the PhC slot nanobeam cavity, where the optical field is tightly confined within the narrow slot. In contrast, as shown in Fig. **S10b**, the electric field in the Si-wire WG is primarily concentrated within the silicon core. Due to the inhomogeneous slot width in the slot nanocavity, unlike in the uniform Si-wire WG, the effective nonlinear coefficient is defined as an average value over a fixed-length cavity. Furthermore, given the distinct nonlinear refractive index of MEH-PPV ($n_2 = 8.5 \times 10^{-17}\ \text{m}^2/\text{W}$) and Si ($n_2 = 4.5 \times 10^{-18}\ \text{m}^2/\text{W}$), we estimate the nonlinear contribution of each material separately. The total effective nonlinear coefficient (γ_{eff}) is obtained by weighting the individual nonlinear coefficients according to the fractional optical energy, $E_{a.u.}$, confined within each material [9]. The resulting expression for γ_{eff} is given by:

$$\gamma_{\text{eff}} = \sum_L \frac{2\pi n_2}{\lambda A_{\text{eff}}} \cdot E_{a.u.}/L \qquad (S30)$$

where λ is the wavelength of the incident light, n_2 is the nonlinear refractive index of the material, L is the device length, and A_{eff} represents the effective mode area, which is defined as [10]:

$$A_{\mathrm{eff}} = \frac{\left(\iint_{-\infty}^{\infty} |E(x,y)|^2 \mathrm{d}x\mathrm{d}y\right)^2}{\iint_{-\infty}^{\infty} |E(x,y)|^4 \mathrm{d}x\mathrm{d}y} \quad (S31)$$

where $|E(x,y)|^2$ represents the modal intensity distribution of the electric field. Based on the field distribution shown in Fig. **S10**, the effective mode area A_{eff} at the center of the 80 nm-wide slot nanocavity is estimated to be approximately $0.125\ \mu\mathrm{m}^2$. For comparison, the A_{eff} of a standard Si-wire waveguide is approximately $0.189\ \mu\mathrm{m}^2$. Figure **S11** illustrates the calculated effective nonlinear coefficients (γ_{eff}) of the PhC slot nanobeam cavities as a function of the central slot width. The results reveal an exponential increase in γ_{eff} with decreasing slot width, attributed to the increasingly tight optical field confinement within the narrow slot. Furthermore, the slot nanocavity exhibits a significantly higher effective nonlinear coefficient compared to the standard Si-wire WG ($\gamma_{WG} \approx 96\ \mathrm{m}^{-1}\mathrm{W}^{-1}$), primarily due to the strong field confinement of the slot and the high Kerr nonlinearity of the polymer MEH-PPV.

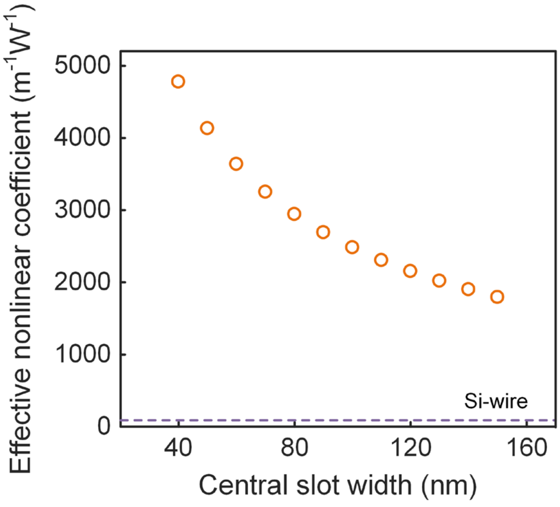

Fig. S11. Effective nonlinear coefficients of the PhC slot nanobeam cavity and the standard Si-wire WG. The orange dots represent the calculated γ_{eff} for various slot widths in the PhC slot nanobeam cavity. The purple dashed line indicates the γ_{eff} value for the standard Si-wire WG.

S7. Fabrication and Devices Details

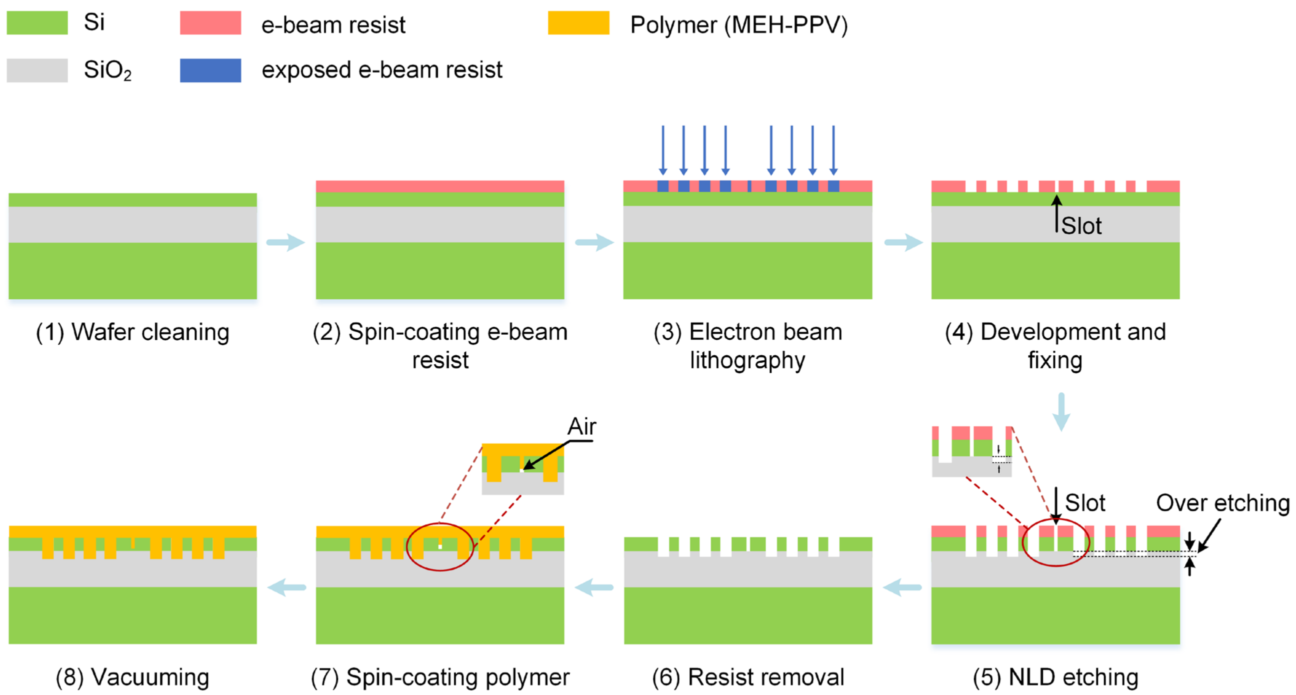

Fig. S12. Fabrication process of optical Kerr switching devices.

Figure **S12** illustrates the fabrication process of the optical Kerr switching devices. The devices are fabricated on a standard silicon-on-insulator (SOI) wafer comprising a $220\ \mathrm{nm}$ top silicon layer and a $2\ \mu\mathrm{m}$ buried oxide layer. Electron-beam lithography (EBL) is employed to define the device patterns on the SOI wafer using a high-resolution e-beam resist. The developed patterns are subsequently transferred into the silicon layer through magnetic neutral loop discharge (NLD) plasma etcher, in which plasma is generated along magnetic cusps by an externally applied radio-frequency (RF) electric field [11]. The NLD etching technique offers distinct advantages, including high ionization efficiency for etching high-aspect-ratio structures and excellent plasma uniformity across large wafer areas, ensuring etch consistency and process reliability. Due to the extreme aspect ratio of the ultra-narrow slot, the etch depth inside the slot is typically shallower than that of the surrounding regions. To counteract this effect, an over-etching step is applied to ensure the final slot depth reaches the design value, as shown in the inset of Fig. **S12(5)**. Following etching, the dissolved MEH-PPV is spin coated onto the chip. To ensure complete infiltration of the polymer into the ultra-narrow slots, residual air is removed under vacuum, as shown in the inset of Fig. **S12(7)**. Figure **S13** presents the

scanning electron microscope (SEM) images of the fabricated slots and air holes with varying dimensions. The results demonstrate that even the narrowest slots down to 40 nm in width and air holes with a radius of 120 nm exhibit excellent sidewall verticality and structural uniformity. This high-fidelity pattern transfer confirms the reliability of the fabrication process and ensures precise preservation of critical structural parameters, which is essential for achieving the designed optical performance of the all-optical switching devices.

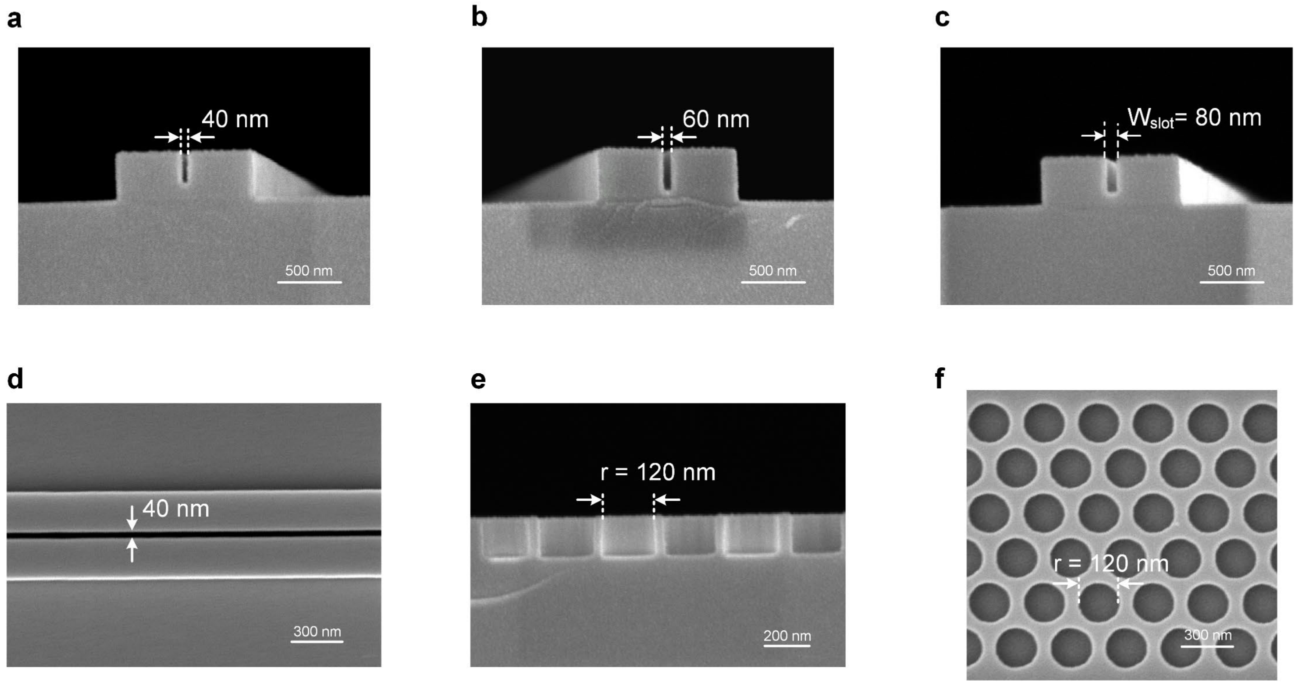

Fig. S13. Scanning electron microscopy images of the fabricated slots and air holes of different sizes. a. b. c. SEM image of the central slot cross-section before spin-coating the polymer, with slot widths of 40 nm (**a**), 60 nm (**b**) and 80 nm (**c**). Scale bar, 500 nm. **d.** SEM top view of the 40 nm wide slot waveguide. Scale bar, 300 nm. **e.** SEM image of the air holes cross-section with a radius of 120 nm. Scale bar, 200 nm. **f.** SEM top view of the air holes with a radius of 120 nm. Scale bar, 300 nm.

To evaluate device-to-device variability arising from fabrication tolerances, we fabricate twelve nominally identical SOH nanocavity devices on the same chip using the same design parameters as those reported in the main text. Figure **S14** summarizes the measured resonance wavelength and loaded Q-factor of Fano mode 1 extracted from the transmission spectra of these devices. The resonance wavelength exhibits a spread from approximately 1530 nm to 1540 nm, while the corresponding loaded Q-factor varies between about 1500 and 2500. Such variations are primarily attributed to inevitable fabrication-induced nonuniformities, including fluctuations in polymer spin-coating

thickness, etching-induced deviations in slot width and air-hole dimensions, as well as local thickness variations of the SOI top silicon layer (210-230 nm). Despite these variations, all measured devices consistently exhibit the characteristic Fano line shape and well-defined cavity resonances, indicating that the formation of the Fano response is tolerant to realistic fabrication variations. These statistics confirm that the reported device performance is representative rather than anecdotal, and that realistic process variations can be readily accommodated through parameter scanning.

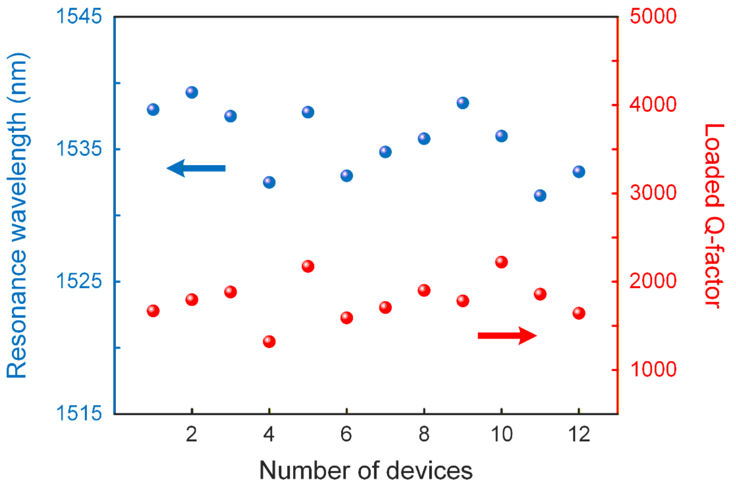

Fig. S14. Statistical distribution of the resonance wavelength (left axis) and loaded Q-factor (right axis) for identical devices fabricated on the same chip.

In the experiment, light is coupled into and out of the chip using photonic crystal (PhC) grating couplers, with a measured coupling loss of approximately -6 dB/facet, as shown in Fig. **S15**. The intrinsic on-chip insertion loss (*IL*) of the device, excluding grating-coupler losses, is approximately -2 dB to -4 dB (as shown in Fig. **2**), which mainly originates from mode mismatch between the bus waveguide and the slot PhC nanobeam cavity. In future implementations, the overall optical throughput could be improved by optimizing the coupling efficiency and reducing the intrinsic insertion loss, for example through improved mode matching or by adopting edge-coupling schemes instead of vertical grating couplers.

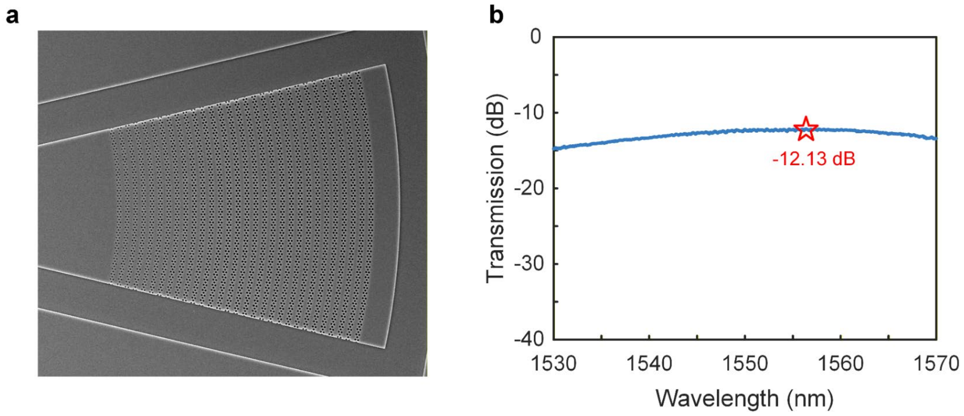

Fig. S15. SEM images and transmission spectrum of the grating coupler.

S8. Thermal Effects of Polymers

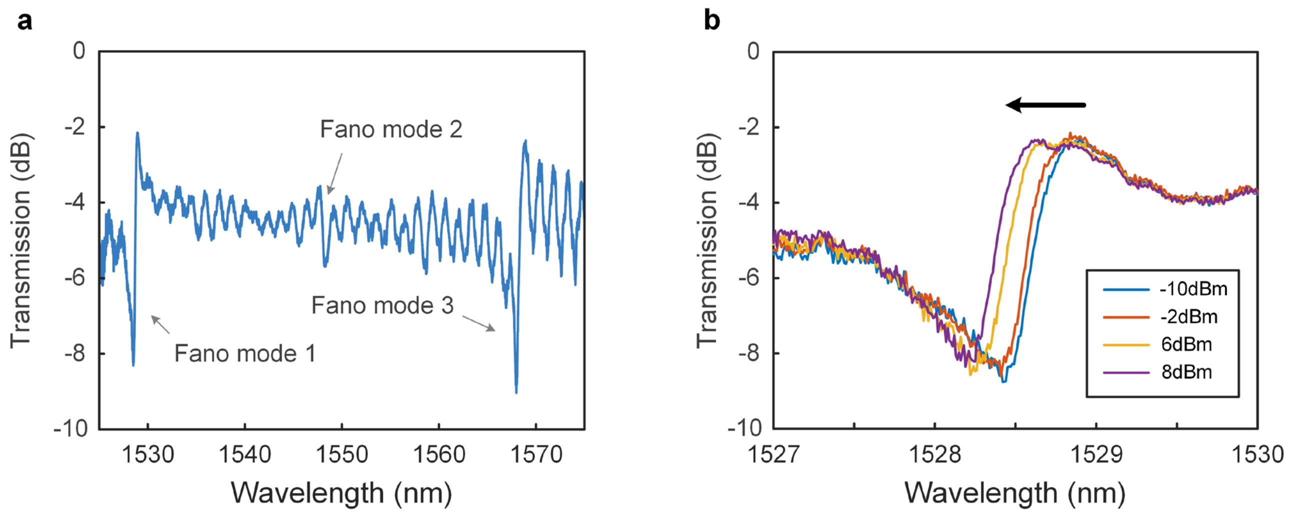

Fig. S16. Transmission spectrum of the fabricated samples with and without pumping. a. Experimental transmission spectrum of the Fano mode 1, Fano mode 2 and the Fano mode 3 without pumping. **b.** Transmission spectrum of Fano mode 1 at different input pump energies.

Due to the high Q-factor and ultra-small mode volume of the slot nanocavity, the optical field density within the cavity is exceptionally high, resulting in significant heat generation in the polymer material. Owing to the relatively slow thermal diffusion rate, typically on the order of nanoseconds to microseconds as explained later, the accumulated heat gradually reaches a steady-state thermal equilibrium. The resulting temperature rise leads to a variation in the local refractive index within the nanocavity, which can be described by the following expression:

$$\Delta n_{thermal} = \frac{\mathrm{d}n}{\mathrm{d}T} \cdot \Delta T \qquad (S32)$$

where $\frac{\mathrm{d}n}{\mathrm{d}T}$ is the thermo-optic coefficient of the material, and ΔT denotes the temperature variation within the nanocavity, depending on factors such as the intracavity optical power, the mode volume of the nanocavity, and the thermal conductivity of the surrounding materials.

To avoid potential damage to the primary device during high-temperature thermal effect measurements, a separate but structurally identical device is fabricated specifically for this characterization. Figure **S16a** shows the transmission spectrum of the new samples without pumping, featuring three distinct resonances corresponding to Fano mode 1, 2 and 3. Under continuous-wave

excitation, the resonance wavelength exhibits a clear power-dependent shift arising from the combined action of the Kerr nonlinearity and thermally induced refractive index changes. As shown in Fig. **S16b**, when the pump wavelength is tuned to Fano mode 3, a clear blueshift of mode 1 is observed with increasing input pump power. To quantitatively separate the Kerr and thermal contributions, we first calculate the Kerr-induced refractive index change using the temporal coupled-mode theory (TCMT) model described in Section **S2**, together with the independently calibrated nonlinear coefficient of the polymer. Subsequently, the remaining contribution, obtained by subtracting the Kerr-induced shift from the experimentally measured total shift, can therefore be attributed to thermal effects, as summarized by the red scatter points in Fig. **S17**. From this analysis, we extract an effective thermal tuning efficiency of approximately $\mathrm{d}\lambda/\mathrm{d}P \approx -0.434\ \mathrm{nm/mW}$. Using the experimentally extracted thermal tuning efficiency, we estimate the steady-state temperature rise at the operating point used in the main switching experiments. At the saturation threshold reported in the main text (switching energy of 63 fJ/bit), the corresponding average on-chip pump power is approximately 1.26 mW. This yields an estimated steady-state temperature increase of approximately $\Delta T \approx 5.9\ ℃$, using the reported thermo-optic coefficient of MEH-PPV ($\mathrm{d}n/\mathrm{d}T \approx -1 \times 10^{-4}\ \mathrm{K}^{-1}$) [12,13].

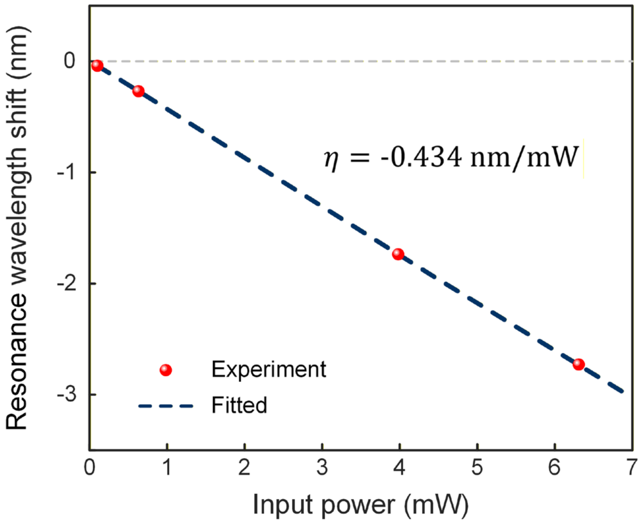

Fig. S17. Resonance wavelength shifts caused by the thermal effect at different input powers.

Additionally, to evaluate the thermal stability of the polymer material (MEH-PPV), we perform complementary thermal analysis using thermogravimetric analysis (TGA) and differential scanning calorimetry (DSC). TGA is conducted in a nitrogen atmosphere to measure the thermal decomposition point of MEH-PPV. As shown in Fig. **S18**, we test different samples: samples 1-3 are MEH-PPV films of varying weight, prepared by dissolving MEH-PPV powder in toluene, depositing it onto substrates,

and drying them to obtain films of approximately 26 μm thickness; Sample 4 is the original solid MEH-PPV powder. The TGA results indicate that the material begins to decompose around 350 °C, which is significantly higher than the operating temperatures used in our experiments, suggesting that thermal degradation under typical experimental conditions is unlikely to occur.

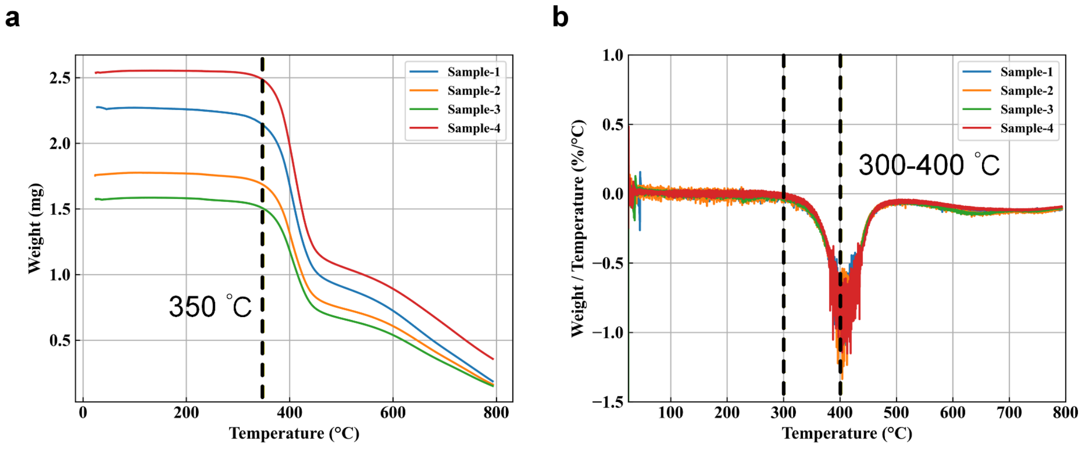

Fig. S18. Thermogravimetric analysis (TGA) of MEH-PPV samples in a nitrogen atmosphere.

DSC measurements are carried out under nitrogen atmosphere to evaluate the melting point and thermal response of the material. As shown in Fig. **S19**(a) and (b), no prominent peaks associated with melting are observed during both the first heating and second heating, indicating that the melting point of MEH-PPV is above 320 °C (positive heat flow corresponds to exothermic (heat-releasing) processes, while negative heat flow corresponds to endothermic (heat-absorbing) processes). Additionally, based on previous TGA data, it is likely that the polymer begins to decompose before it melts. The first peak observed in the DSC measurement corresponds to the thermal balance of the system and is independent of the material itself. These results further confirm the thermal stability of MEH-PPV, ensuring that thermal effects under operational conditions do not significantly contribute to performance degradation. For future scaling toward higher power handling or broader operating scenarios, the thermal robustness of the nonlinear polymer can be further enhanced through materials and process engineering. Building on recent advances in thermally stable and crosslinked organic polymer systems [14], the nonlinear polymer may be spin-coated in combination with an imprint resist or cross-linkable host matrix, followed by ultraviolet curing. Such approaches have been shown to increase the glass transition temperature and suppress thermally induced polymer reflow, thereby providing a viable pathway for improving the thermal tolerance of hybrid silicon-organic nanophotonic devices.

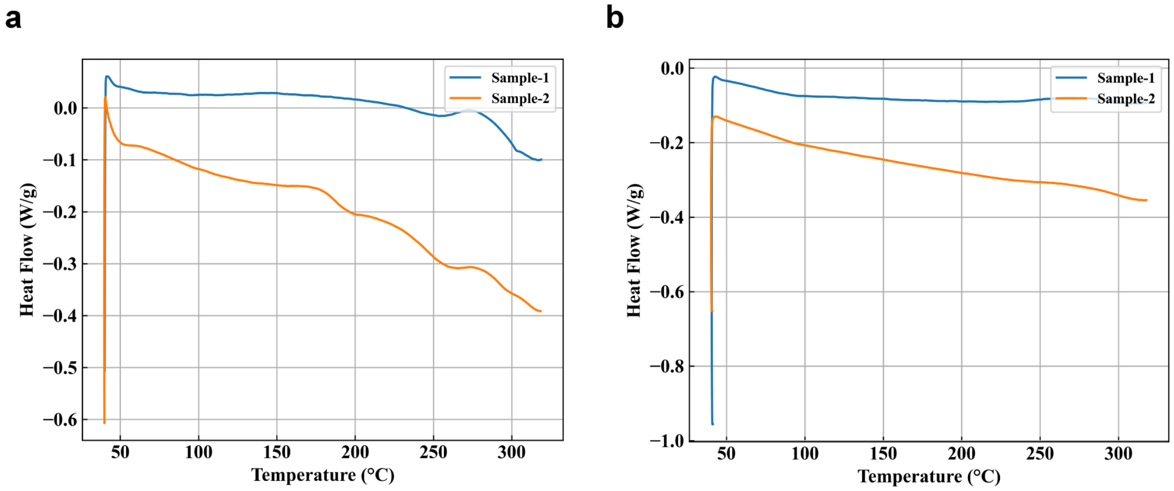

Fig. S19. Differential scanning calorimetry (DSC) of MEH-PPV samples under nitrogen atmosphere during the first heating (a) and second heating (b).

Notably, the extracted thermal tuning efficiency exhibits a negative sign, which is consistent with the reported negative thermo-optic coefficient of the polymer MEH-PPV ($\mathrm{d}n/\mathrm{d}T < 0$), and opposite to the positive thermo-optic coefficient of silicon ($\mathrm{d}n/\mathrm{d}T \approx 1.85 \times 10^{-4}\ \mathrm{K}^{-1}$). This observation indicates that the thermally induced resonance shift is dominated by the polymer-filled slot region, rather than by the surrounding silicon rails. Therefore, this thermally induced refractive index reduction partially offsets the Kerr-induced redshift, thereby degrading the overall signal quality. Transient thermal simulations performed using COMSOL Multiphysics reveal that the thermal response time of the nanocavity is approximately 7.6 μs (see Fig. **S20**). Under high-speed PRBS excitation, where the bit period (25 ps at 40 GBaud) is several orders of magnitude shorter than the thermal response time of the nanocavity, this temperature rise is governed solely by the average optical power of the pump ($\Delta n_{thermal} \propto P_{AVG}$) and remains quasi-static during bit-level modulation. To mitigate this effect, a pre-shift of the input wavelength can be applied, in which the input pump wavelength is slowly scanned from the red-detuned side toward the blue side until the cavity reaches a thermally stabilized operating point [15]. Once thermal equilibrium is established at the target average power, the high-speed modulation is applied, effectively compensating the negative thermo-optic drift and stabilizing the cavity response during ultrafast signal modulation.

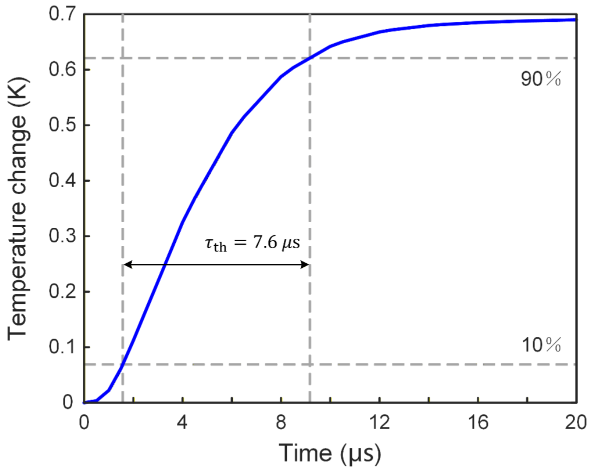

Fig. S20. Temperature transient response at the device center.

S9. Experimental Setup for Optical Measurement

The high-speed data generation and BER measurements are carried out using an integrated SHF communication test platform, consisting of a synthesized clock generator (SHF 78210 D), a bit-pattern generator (SHF 12104 A), and an error analyzer (SHF 11104 A). The synthesized clock generator provides a low-jitter reference clock that synchronizes both the transmitter and receiver, ensuring deterministic timing and accurate bit-by-bit error analysis. The bit pattern generator generates a PRBS sequence of length $2^7 - 1$ at the target symbol rate, which is operated in a cyclic mode to provide a continuous data stream for driving the modulator. Meanwhile, the error analyzer performs real-time BER counting by synchronizing the detected electrical signal with the internally generated reference PRBS pattern. BER counting is initiated only after successful pattern acquisition and stable lock are achieved, ensuring reliable synchronization prior to real-time error counting.

On the receiver side, the switched probe signal is detected using a high-speed photodetector (XPDV2120R, 40-GHz electrical bandwidth), followed by broadband electrical amplification and direct connection to the SHF error analyzer, as shown in Fig. **S21**. By adjusting a variable optical attenuator (VOA) placed before the photodetector (PD), the BER is measured as a function of the received optical power at the PD, as shown in Fig. **4b**. The overall electrical bandwidth of the detection chain is sufficient to support the maximum tested symbol rate, ensuring that the measured BER performance is not limited by receiver bandwidth.

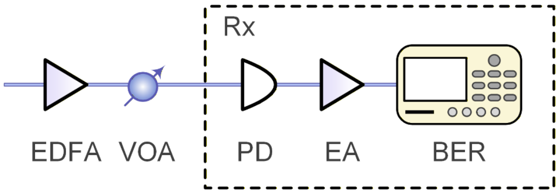

Fig. S21. Experimental setup for the BER measurements.

The PRBS sequence (length $2^7 - 1$) is generated in a continuous cyclic mode and measured over an extended duration. At a symbol rate of 40 GBaud and a measurement time of 2 s, a total of more than 8×10^{10} bits are analyzed. With zero errors observed, Poisson statistics yield an upper BER bound of 3.75×10^{-11} at a 95% confidence level, which is well below the commonly accepted error-free criterion of 10^{-9}, thereby substantiating the reported error-free operation.

S10. Femtojoule 30 GBaud Switching Results

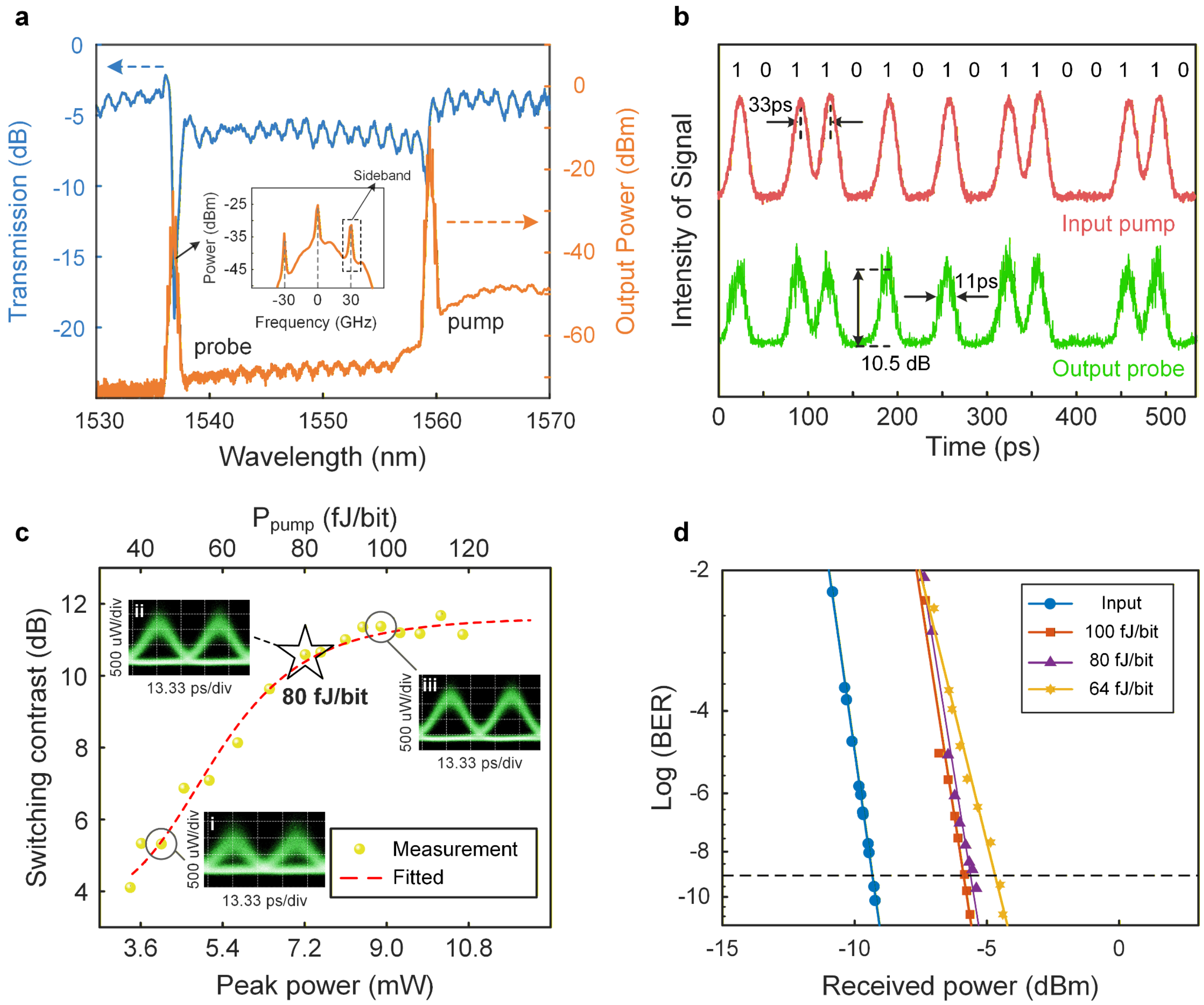

Fig. S22. Femtojoule 30 GBaud all-optical switching with error-free performance. a. Transmission spectrum (blue) and the output spectrum obtained after the device (orange), measured with a resolution bandwidth of 0.02 nm. The inset shows the modulated probe light with distinct modulation sidebands. The input pump light energy is 80 fJ/bit corresponding to a peak power of 7.2 mW. **b.** Input pump light (red) and output probe light pulse train (green). **c.** Switching contrast under different switching energies with eye diagrams at 45 fJ/bit (i), 80 fJ/bit (ii), and 100 fJ/bit (iii). The red dashed line fits the trend of the experimental results (yellow dots). **d.** BER curves of original pump light and the modulated probe light with switching energies of 64 fJ/bit, 80 fJ/bit and 100 fJ/bit, respectively.

We experimentally evaluate the switching performance of the device at a high signal rate of 30 GBaud, with the experimental setup shown in Fig. **3a**. Figure **S22a** illustrates the output spectrum measured by an optical spectrum analyzer (OSA), corresponding to an on-chip input peak power of 7.2 mW for the pump light. The estimated switching window is approximately 11 ps, calculated from the signal rate and duty cycle (i.e., $33\%/(30\ \mathrm{Gb/s}) \approx 11\ \mathrm{ps}$), corresponding to a switching energy of 80 fJ/bit (i.e., $7.2\ \mathrm{mW} \times 11\ \mathrm{ps} \approx 80\ \mathrm{fJ/bit}$). To mitigate polymer-related thermal effects, both the probe and pump wavelengths are pre-shifted by 0.13 nm and set to 1536.76 nm and 1559.42 nm, respectively. As shown in the inset, distinct modulation sidebands appear in the output spectrum of the probe light, with the first sideband located 30 GHz from the main peak, confirming effective modulation. Figure **S22b** displays the time-domain temporal switching response of the probe light, measured using a communication signal analyzer (CSA). The red solid line represents the original 30 GBaud signal waveform, while the green solid line shows the time-domain waveform of the modulated probe light at the output. Both exhibit a pulse width of 11 ps and a signal train period of 33 ps, consistent with the expected bit period. The second probe pulse temporally aligns with the pump pulse and achieves a switching contrast of 10.5 dB. Figure **S22c** shows the evolution of the switching contrast as a function of the input pump peak power. The switching contrast increases with the pump power and eventually reaches saturation, exhibiting a trend consistent with the observed improvement in eye diagram quality. This behavior aligns well with both the 40 GBaud measurements and the results obtained from prior simulations. Bit error rate (BER) curves for both the original pump light and the modulated probe light at varying input energies are measured, as shown in Fig. **S22d**. Error-free performance is realized ($\mathrm{BER} < 10^{-9}$) when the switching energy exceeds 64 fJ/bit. In summary, the 30 GBaud all-optical switching demonstrates high modulation fidelity and error-free performance, underscoring its potential for energy-efficient, high-speed optical communication systems.

S11. Long-term Stability Measurements

To further evaluate the operational stability under sustained high-speed switching, a long-term stability measurement is performed under fixed operating conditions corresponding to the saturation threshold reported in the main text, without employing any feedback control or external temperature stabilization. Specifically, the device is continuously operated at a switching energy of 63 fJ/bit, a signal rate of 40 GBaud, and a duty cycle of 33% for a duration of 30 minutes. During the measurement, the received optical power at the photodetector (PD) is fixed at -1.8 dBm, and the bit-error rate (BER) is continuously monitored using the experimental setup described in Fig. **3a**. The measured BER remains below 10^{-9} throughout the entire 30-minute interval, confirming sustained error-free operation, as shown in Fig. **S23**. During the first ~20 minutes, the BER is essentially constant, indicating stable switching behavior under steady-state thermal conditions. In the subsequent ~10 minutes, a slight increase in the measured BER is observed, which is attributed primarily to slow fluctuations in the grating-coupler coupling efficiency and minor cavity detuning. Importantly, the BER remains well below the error-free threshold, and no abrupt degradation or instability is observed. These results demonstrate that, under the reported operating conditions, the modest and quasi-static temperature rise does not compromise the long-term stability of the all-optical switching performance. For future implementations targeting extended deployment times or more demanding environments, additional stabilization strategies—such as active feedback control of the pump wavelength or optical power, chip-level temperature control, and optimized device packaging—can be readily incorporated to further enhance system robustness.

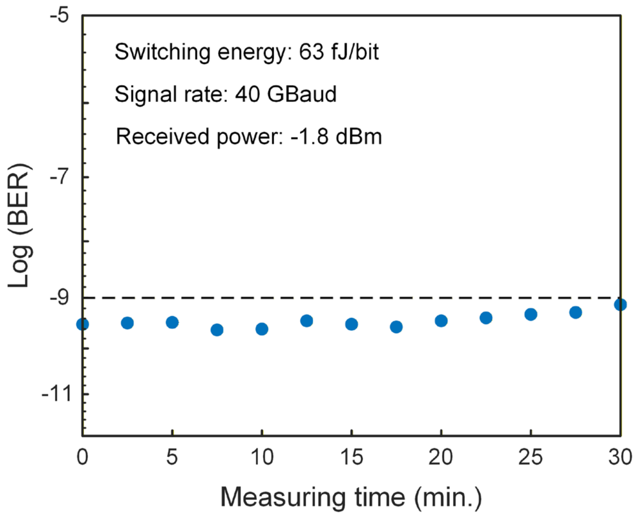

Fig. S23. BER stability measurement over 30 minutes under continuous 40-GBaud switching operation.

S12. Performance Comparison of On-chip All-optical Switches

A variety of chip-scale all-optical switches has been demonstrated across different material platforms and nonlinear mechanisms. Here, we provide a detailed comparison of four key performance metrics: peak power, switching energy, switching time, and switching contrast, as well as criterions that evaluates how closely the experimental results align with all-optical signal processing, i.e., duty cycle and repetition rate, as shown in Table **S1**. The criterions roughly reflect the practical application potential of the devices. The basic demos involve measuring the dynamic characteristics of the switching device using pump-probe techniques (p-p), where the input signal has a very low repetition rate. As the repetition rate increases, the average dissipated power also increases, potentially introducing thermal effects that can impact device performance. Additionally, the incomplete recovery of the signal waveform could affect subsequent bit streams, a phenomenon known as patterning effects. The second level involves using fixed-pattern data streams with high duty cycles and high repetition rate, such as predefined 4-bit repeating data sequences, which are more relevant for practical applications. The demos mostly close to practical applications build upon the second by applying PRBS data streams for bit-error rate measurements, ensuring the performance of device in practical, real-world scenarios.

Table S1 Comparison of the performance of various on-chip all-optical switches.

Principle	Peak Power	Switching Time	Switching Energy	Switching Contrast	Duty Cycle (%)	Repetition Rate
BFD+TPA/LA [16]	47 µW *	35 ps	0.66 fJ/bit	10 dB	1.4×10^{-2} *	10 MHz $^{p\text{-}p}$
	NA	NA	10.2 fJ/bit	NA	NA	40 Gbps 4-bit pulses
BFD+FCE [17]	10.33 mW *	NA	310 fJ/bit	3 dB	2.4×10^{-1} *	80 MHz $^{p\text{-}p}$
Saturable absorption [18]	167 mW *	260 fs	35 fJ/bit	3.5 dB	2.1×10^{-4} *	10 MHz $^{p\text{-}p}$
$\chi^{(2)}$ nonlinearity [19]	2 W *	46 fs	80 fJ/bit	3 dB	1×10^{-3} *	250 MHz $^{p\text{-}p}$
FCE [20]	5 mW *	30 ps	100 fJ/bit	3 dB	NA	5 Gbps 4-bit pulses
FCE [21]	60 mW *	70 ps	460 fJ/bit	NA	7.7×10^{-3} *	10 MHz $^{p\text{-}p}$
Kerr effect [22]	370 mW *	NA	1124 fJ/bit *	7.7 dB	12.81 *	42.7 Gbps 2^{31}-1 PRBS
Kerr effect [23]	264 mW *	18 ps	720 fJ/bit	NA	2.7×10^{-2} *	100 MHz $^{p\text{-}p}$
ISBT [24]	4.3 W *	1.2 ps	520 fJ/bit	7 dB	9.12×10^{-4} *	76 MHz $^{p\text{-}p}$
**This work**	**7.5 mW**	**9 ps**	**63 fJ/bit**	**8.3 dB**	**33**	**40 Gbps 2^7-1 PRBS (Error-free)**

Notes: NA—not available, BFD—band-filling dispersion, TPA—two-photon absorption, LA—linear absorption, FCE—free-carrier effect, ISBT—inter-sub-band transition, p-p—pump-probe measurement, PRBS—Pseudo-Random Binary Sequence, *—calculated based on available data: The switching energy is defined as the product of the on-chip peak pump power and the effective temporal width of the input pump "1"-bit, while the duty cycle is given by the product of this pulse width and the pulse repetition rate.

Supplementary References

1. Peng, B. et al. Parity–time-symmetric whispering-gallery microcavities. *Nat. Phys.* **10**, 394–398 (2014).

2. Van, V. *Optical Microring Resonators: Theory, Techniques, and Applications*. (CRC Press, 2016).

3. Yu, P. et al. Fano resonances in ultracompact waveguide Fabry-Perot resonator side-coupled lossy nanobeam cavities. *Appl. Phys. Lett.* **103**, 091104 (2013).

4. Volksen, W. et al. Low Dielectric Constant Materials. *Chem. Rev*. **110** (1), 56-110 (2010).

5. Kim, C. et al. Parity-time symmetry enabled ultra-efficient nonlinear optical signal processing. *eLight.* **4**, 6 (2024).

6. Peng, B. et al. What is and what is not electromagnetically induced transparency in whispering-gallery microcavities. *Nat Commun.* **5**, 5082 (2014).

7. Quan, Q. et al. Deterministic design of wavelength scale, ultra-high Q photonic crystal nanobeam cavities. *Opt. Express* **19**, 18529-18542 (2011).

8. Govind P. Agrawal. *Nonlinear Fiber Optics*. (Academic Pr, 1989).

9. Wang Y. et al. Enhanced optical nonlinearity in a silicon-organic hybrid slot waveguide for all-optical signal processing. *Photon. Res.* 10, 50–58 (2022).

10. Oulton, R.F. et al. Confinement and propagation characteristics of subwavelength plasmonic modes. *New J. Phys.* **10**, 105018 (2008).

11. Uchida, T. et al. Magnetic neutral loop discharge (NLD) plasmas for surface processing. *J. Phys. D: Appl. Phys.* **41**, 083001 (2008).

12. Rostra, G.J. et al. Thermo-optic response of MEH-PPV films incorporated to monolithic Fabry-Perot microresonators. *Dyes and Pigments*. **182**, 108625 (2020).

13. Zhang, Z. et al. Thermo-optic coefficients of polymers for optical waveguide applications. *Polymer (Guildf).* **47**, 4893–6 (2006).

14. Xu, H. et al. Ultrahigh Performance Cross-Linkable Organic Electro-Optic Material for Hybrid Modulators. *Chemistry of Materials* **37**, 12 (2025).

15. Zhang, H. et al. Soliton Microcombs Multiplexing Using Intracavity-Stimulated Brillouin Lasers. *Phys. Rev. Lett*. **130**, 153802 (2023).

16. Nozaki, K. et al. Sub-femtojoule all-optical switching using a photonic-crystal nanocavity. Nat. Photon. 4, 477–483 (2010).

17. Takiguchi, M. et al. All-Optical InAsP/InP Nanowire Switches Integrated in a Si Photonic Crystal. ACS Photon. 7, 1016-1021 (2020).

18. Ono, M. et al. Ultrafast and energy-efficient all-optical switching with graphene-loaded deep-subwavelength plasmonic waveguides. Nat. Photon. 14, 37–43 (2020).

19. Guo, Q. et al. Femtojoule femtosecond all-optical switching in lithium niobate nanophotonics. Nat. Photon. 16, 625–631 (2022).

20. Moille, G. et al. Integrated all-optical switch with 10 ps time resolution enabled by ALD. Laser Photonics Rev. 10, 409 (2016).

21. Tanabe,T. et al. Fast all-optical switching using ion-implanted silicon photonic crystal nanocavities. Appl. Phys. Lett. 90, 031115 (2007).

22. Koos, C. et al. All-optical high-speed signal processing with silicon-organic hybrid slot waveguides. Nat. Photon. 3, 216–219 (2009).

23. Jason S. et al. Picosecond all-optical switching in hydrogenated amorphous silicon microring resonators. Opt. Express 22, 3797-3810 (2014).

24. Hu X. et al. Picosecond and low-power all-optical switching based on an organic photonic-bandgap microcavity. Nat. Photon. 2, 185–189 (2008).
