# Supplementary material for: Femtojoule optical Kerr switching with milliwatt-peak-power in silicon-organic hybrid nanocavity
Source: Nat Commun. 2026 May 15;17:6483. doi: 10.1038/s41467-026-73285-9 (PMC13376617; doi:10.1038/s41467-026-73285-9)
Supplement: Supplementary file 2 — Transparent Peer Review file [file 41467_2026_73285_MOESM2_ESM.pdf]

nature portfolio

Peer Review File

Femtojoule optical Kerr switching with milliwatt-peak-power in silicon-organic hybrid nanocavity

Corresponding Author: Professor Xinliang Zhang

This file contains all reviewer reports in order by version, followed by all author rebuttals in order by version.

Version 0:

Reviewer comments:

Reviewer #1

(Remarks to the Author)
This manuscript submitted by Chen, et al. presents a demonstration of a femtojoule-level all-optical Kerr switch operating at milliwatt peak power, based on a silicon-organic hybrid (SOH) photonic crystal slot nanobeam cavity. By combining small-mode-confinement, high Q resonances and MEH-PPV, the reported device achieves error-free 40 Gbit/s switching with 63 fJ/bit energy consumption, 7.5 mW peak power, and a contrast ratio of 8.3 dB. And, this scheme avoids the use of ultrashort, low-duty-cycle pulses. Overall, I think this manuscript is well organized, solid, and presents interesting technical merits. I expect that the authors can address the following points before my recommendation for publication in Nature Communications.

1. In introduction, the authors mentioned that to enhance the 3-rd nonlinearity for boosting Kerr effect, versatile photonic platforms have been investigated, that's true. Then the authors stated that silicon photonics is preferrable, due to its COMS compatibility. This flow is a bit strange. Since 3-rd nonlinearity and high Kerr effect is critical, silicon nitride cavities with pure Kerr oscillations should be a better choice. After all, as the authors wrote, TPA is a significant consideration in silicon, that is why the authors used SOH. In [eLight eLight 4, 19 (2024)], comprehensive comparison of Kerr-effect in types of optical platforms have been provided. Referring the related reviews, I suggest the authors can find more fundamental motivations beyond the TPA and FCA. In addition, I suggest the authors can provide a table to discuss the performances and practicalities about the Kerr-switching on typical platforms.

2. In introduction, the authors claimed that "the majority of demonstrated devices rely on excitation by ultrashort pulses with Watt-level peak powers and extremely low duty cycles (defined as the ratio of the pulse duration to the repetition period, due to the intrinsically weak optical nonlinearity of available materials". I wonder that the intrinsically weak optical nonlinearity may be not the main limitation. For example, referring the literature [Nature Communications 13, 3138 (2022)], integrated 2D materials can offer high nonlinearity based-on chi(2) for achieving high-efficiency optical switching. I suggest the authors can broaden their discussions.

3. While the manuscript mentions compensating for thermo-optic effects via blue-shifting (Page 8) and highlights the high duty cycle (33%) as a key advantage, it lacks quantitative characterization of the temperature rise under sustained operation or long-term stability data. Given the high average power density inherent in cavity-enhanced structures and the known thermal sensitivity of polymers, this is a critical practical concern. I suggest the authors can add measurements or simulations estimating the temperature rise within the polymer-filled slot under continuous-wave (CW) pumping at the operational peak/average power levels. Or, the authors may consider to add data demonstrating stability over extended periods (e.g., minutes to hours) under the reported switching conditions (63 fJ/bit, 40 GB, 33% duty cycle). This could include monitoring BER, switching contrast, or resonance wavelength drift over time. Moreover, discussion on potential thermal management strategies if significant heating is observed. Related to other materials, e.g. silica microcavities [Phys. Rev. Letters 130(15), 153802 (2023)], I suppose the thermal effect may induces higher impact on Kerr-nonlinearity in the SOH structure.

4. The manuscript briefly mentions the potential for multi-channel operation via WDM in the Discussion (Page 10, Lines 276-278) as a route to Tbps processing. However, no experimental evidence or quantitative analysis supporting this claim is provided. To substantiate this claim and strengthen the impact, the authors may experimentally demonstrate simultaneous switching of at least two independent probe channels (different wavelengths) using a single pump channel on the same

device structure, and/or discuss the practical limitations on channel count and spacing imposed by the cavity design (Q-factor, resonance spacing, mode overlap) and nonlinear material bandwidth.

5. The authors utilized MEH-PPV, a conjugated polymer known to be susceptible to photodegradation (photo-oxidation) under illumination, especially at telecom wavelengths involving higher photon energies than its bandgap. The high intracavity field intensity and sustained high-repetition-rate operation could accelerate this degradation, impacting device lifetime and reliability – a crucial factor for practical applications. I suggest the authors can showcase accelerated lifetime testing under operational optical intensities (peak and average power) relevant to their switching conditions. And, they can include a statement on the current observed operational stability within the timeframe of their reported experiments. For instance, in previous studies, such as [Nature 558, 410 (2018)], the existence of intracavity polymer may decelerate the photon-electron interactions especially when changing the status of Kerr oscillation.

6. In Fig. 3a, the output probe power appears to be relatively low. Are optical amplifiers used before detection? Given common practice in similar experiments, the authors likely employed a filter–amplify–filter scheme prior to detection. However, the figure omits any depiction of amplification components. The absence of this information may lead to confusion for readers attempting to interpret the results or replicate the setup. I suggest adding a brief explanatory note in the figure caption, possibly with relevant references to similar implementations. In addition, one of the merits of using XPM for switching, the output power is relatively higher since it employs interference effect, as comparing to FWM effect that generates new frequency component. I suggest the authors to clarify the reason for low output power of their results and discuss possible ways to further improve the results.

7. While the eye diagrams in Fig. 4a demonstrate reasonably clear openings—with some thickening of the upper eyelid but otherwise good eye clarity and amplitude—it is regrettable that the manuscript provides only a subjective statement: “the eye diagram displays high signal quality.” I suggest that the authors offer a more quantitative and rigorous description of the eye diagram quality, potentially by including standard metrics such as eye height, eye width or Q-factor to support the visual assessment.

8. A variety of chip-scale all-optical switches has been demonstrated across different material platforms and nonlinear mechanisms. In Fig. 5, the authors provide a comprehensive and intuitive comparison between their device and several representative approaches, including those based on free-carrier effects, Kerr nonlinearity, chi(2) effects, and saturable absorption. While this type of comparison is valuable and broadly informative, it inevitably omits certain high-quality works that do not fall neatly into the selected categories. To complement this overview, I recommend the authors consider adding a supplementary table that systematically summarizes the performance metrics (e.g., switching energy, speed, contrast ratio) across a wider range of reported devices. This would help readers more thoroughly assess the relative advantages of the proposed device. For reference, the authors may consider the comparison approach adopted in [Laser Photonics Rev., 10, 409 (2016)], which not only evaluates device-level metrics but also introduces an additional criterion related to how closely the experimental results reflect all-optical signal processing capabilities.

9. In addition, in Fig. 5, the axes (Peak Power vs. Switching Energy) make direct comparison challenging regarding the key claimed advantage of low peak power at high duty cycle. Duty cycle is plotted separately. Furthermore, the trade-off between speed (switching time) and energy is inherent but not explicitly visualized in a way that highlights their achievement of speed with low energy/peak power. The authors can consider adding contour lines of constant average power (Peak Power * Duty Cycle) to Figure 5.

In sum, in my view, this paper offers innovative results and would be interesting for readers in optical time-frequency manipulation, telecommunication, and signal processing. I can recommend its publication in nature communications once my concerns were well addressed.

Reviewer #2

(Remarks to the Author)
This manuscript reports a silicon–organic hybrid (SOH) photonic-crystal (PhC) slot nanobeam cavity implementing two-mode Kerr switching via cross-phase modulation between distinct Fano resonances. The authors claim error-free 40-Gbit/s RZ-OOK operation with 33% duty cycle at 7.5 mW peak power and 63 fJ/bit switching energy, with loaded Q factors ~1.8×10^3 and 2.5×10^3 (bandwidths 108 and 77 GHz), and an electric-field overlap integral of 0.69 between the two modes. They attribute the low energy to ultra-tight confinement (small mode volume) and a high-n2 polymer (MEH-PPV) with negligible nonlinear absorption, and they provide coupled-mode/transfer-matrix modeling and design/fabrication details in the Supplementary Information. Here, my major comments:
1. The reported switching energy (63 fJ/bit at 7.5 mW) is obtained assuming a 40-Gbit/s, 33% RZ duty cycle and an effective “1”-bit width of ~9 ps. Simple multiplication yields 67.5 fJ (7.5 mW × 9 ps), slightly above 63 fJ; at 33% of a 25-ps period the nominal bit-“on” time is 8.25 ps, which would give 61.9 fJ. Please reconcile the exact on-chip pulse width used in the energy calculation, specify whether the 7.5 mW is measured in-fiber, at the chip facet, or as intracavity peak, and propagate the uncertainty (coupling losses, calibration) into the stated 63 fJ/bit. The same issue appears at 30 Gbaud (7.2 mW, 11 ps → ~79 fJ/bit, reported as 80 fJ/bit).
2. The Authors state the switching time is “ultimately limited by the photon lifetime” of the Fano resonances (1.5 ps and 2.1 ps). Yet the demonstrated switching window is ~9 ps at 40 Gbaud, substantially longer than those lifetimes. Please clarify what sets the observed 9-ps window (modulator waveform shaping, cavity loading, filter response, or thermal detuning compensation) and quantify the contributions with measured instrument responses and deconvolution, not only TCMT fits.

3. The narrative emphasizes negligible TPA/FCA due to polymer nonlinearity, but a PhC slot on SOI will still localize a finite fraction of energy in silicon rails. Provide a quantitative breakdown of modal energy in polymer vs silicon at both resonances, estimate TPA-induced carrier densities at the stated peak powers, and bound FCA-related loss/phase shift during a bit slot. If reverse-biased junctions are unnecessary, justify with measurements (e.g., pump-probe recovery traces vs peak power showing no carrier tail).
4. The authors attribute the observed bistable transmission exclusively to the Kerr effect of the polymer, yet no quantitative evidence is provided that rules out thermal contributions. Given the relatively high average powers (milliwatt-level peak with 33% duty cycle) and the limited heat dissipation of the SOH nanobeam cavity, thermal refractive index changes could induce thermal bistability or hysteresis indistinguishable from Kerr bistability in static or slow scans. Please include time-resolved transmission traces at different modulation frequencies to demonstrate that the response follows the instantaneous pump envelope (picosecond-scale) rather than a microsecond-scale thermal process. Additionally, estimate the local temperature rise and compare it to the dn/dT of MEH-PPV and silicon to quantitatively exclude thermal effects. Without such evidence, it remains unclear whether the reported bistability is purely optical or partially thermally driven.
5. Operation requires pre-shifting the pump/probe wavelengths (e.g., 0.15 nm at 40 Gbaud; 0.13 nm at 30 Gbaud) to compensate the polymer's negative thermo-optic shift. Please provide quantitative thermal time constants, steady-state temperature rise vs average power, and eye/BER stability over ≥10–60 minutes at the saturation threshold. Add drift traces of resonance wavelength under PRBS drive, and demonstrate resilience to environmental fluctuations. The Supplement discloses cumulative thermal effects on microsecond scales; show that pattern-dependent baseline wandering is negligible at line-rate for realistic traffic statistics. Also, address polymer photostability and possible irreversible index changes over repeated high-duty-cycle stressing (lifetime test).
6. The overlap integral between the two Fano modes is cited as 0.69 from simulation; provide experimental corroboration (e.g., cross-phase modulation efficiency vs detuning for both mode pairs, or heterodyne measurements of XPM-induced resonance pulls). Include sensitivity of overlap and Q to fabrication tolerances (slot width, hole radius/spacing, bus gap) and report device-to-device statistics (N≥10), not just a single exemplar. The FP-induced Fano shape is central; demonstrate that the 1.4-nm ripple from facet/air-hole reflections does not degrade BER across wavelength drift.
7. Figure 5 compares peak power, duty cycle, energy/bit, contrast, and switching time across mechanisms. Ensure apples-to-apples normalization: for Kerr devices, quote on-chip energies with explicit coupling losses; for saturable-absorber/χ(2) devices, indicate whether femtosecond-pulse, ultra-low duty-cycle operation is being compared to your high-duty-cycle case. Provide a table listing each data point's definitions (duty cycle, PRBS length, pattern dependence criteria, BER floor) and references.
8. The Authors report error-free performance (BER ≤ 10^-9) beyond 50 fJ/bit with power penalties trending with contrast. Please provide full receiver configuration (detector type, bandwidth, reference clocking), number of bits tested to substantiate "error-free", confidence bounds, bathtub curves/jitter decomposition, and penalties at fixed BER (e.g., 10^-3 pre-FEC and 10^-9 post-FEC). Also clarify whether the probe channel is spectrally filtered tightly enough to suppress pump leakage for all thermal drifts.
9. The effective nonlinear coefficient is estimated at 2952 $m^{-1}W^{-1}$ (31× a strip Si waveguide) and n2 for MEH-PPV is stated as "about 20× silicon." Provide the exact n2 value used at 1550 nm, its dispersion, and experimental validation on your structures (z-scan on films, self-/cross-phase modulation on straight SOH waveguides). Without a direct measurement on the fabricated devices, it is difficult to disentangle n2, mode volume, and Q-loading contributions. Include error bars from uncertainties in field overlap and effective area.
10. The Supplement shows excellent SEMs down to 40-nm slots fabricated by NLD etching. Please include statistical distributions of as-fabricated slot width, sidewall roughness (Rq), and cavity resonance wavelengths across the die/wafer, and correlate these to the optical metrics (Q, ER, IL) and the achieved switching energy. Report yield of devices meeting the 40-Gbaud spec and how sensitive performance is to polymer thickness variation and infiltration completeness.
Here, my minor comments:
1. Define all symbols at first use and maintain consistent notation between main text and SI (e.g., Q_i/Q_e/Q_L, FE, ER, V_eff, γ, κ, δ, τ_ph).
2. Figures: in Fig. 2b, label the ripple period (1.4 nm) on the plot; in Fig. 3b inset, annotate the 40-GHz sidebands; ensure all axes have units and all spectra indicate resolution bandwidths. Eye diagrams in Fig. 4a would benefit from identical scaling for fair visual comparison.
3. Clarify whether insertion loss values are on-chip (excluding grating/edge coupling) or end-to-end. If on-chip, report coupling loss separately for completeness.

Reviewer #3

(Remarks to the Author)
In the presented paper, the authors experimentally demonstrated nonlinear optical switching with both low peak pump power of 7.5 mW and high duty cycle of 33% for 40 Gbps signals. The required peak power of 7.5 mW is two orders of magnitude lower than previously reported Kerr switches, showing the technological advancement of the proposed device. The manuscript is well constructed, and the results are clearly shown. I would like to recommend publication of the presented paper if the following comments are addressed.

1. Throughout the experiment section, the transmission quality of the probe light is measured. However, the motivation for measuring the transmission quality of the probe light is not shown clearly. This experiment might be connected to the optical wavelength conversion, but it should be clearly stated. Moreover, the required performance metrics (such as power penalty and so on) for this application would be required to be clearly shown together with the experimental results.

2. In the results of Fig. 4b, the signal received power reaches 0 dBm. As compared to the peak pump power, this signal power might be large enough to cause other nonlinear effects such as SPM. Could the authors comment on this? Also, it would be helpful for the readers if any nonlinear degradation of signals were observed during the experiments.

3. At line 216, the words “switching time” would be better written as “switching window” or “switching time width” because the switching time typically correspond to the rise/fall time.

4. In Fig. 5, the results from [1] were not shown, although [1] shows a very low switching energy (sub-fJ). Could the authors comment on this?

5. It would be helpful for readers if the authors could discuss the reason of selecting the duty cycle of 33%. Also, the discussion of future improvements (lowering the peak power, increasing the duty cycle and so on) would be helpful for the readers.

Version 1:

Reviewer comments:

Reviewer #1

(Remarks to the Author)
The authors well addressed my concerns. And I also read the response to other referees.
After revision, discussion of this manuscript has been well reinforced and depth of this paper has been enhanced for readers. More technical details are also better clarified. I recommend to publish this work in Nature Communications.

Reviewer #2

(Remarks to the Author)
The revised manuscript has been substantially improved in clarity, methodological rigor, and experimental validation; the authors have addressed the reviewers’ comments thoroughly and convincingly, and in its current form the paper merits publication.

Reviewer #3

(Remarks to the Author)
The authors have answered all questions from the reviewers and substantial changes have been made to the manuscript improving the quality of the paper. Background information and comparison with other systems have been added, which resulted in clearer statements of the significance of this work. I have no further comments or questions regarding the revised manuscript.

Open Access This Peer Review File is licensed under a Creative Commons Attribution 4.0 International License, which

permits use, sharing, adaptation, distribution and reproduction in any medium or format, as long as you give appropriate credit to the original author(s) and the source, provide a link to the Creative Commons license, and indicate if changes were made.
In cases where reviewers are anonymous, credit should be given to 'Anonymous Referee' and the source.
The images or other third party material in this Peer Review File are included in the article's Creative Commons license, unless indicated otherwise in a credit line to the material. If material is not included in the article's Creative Commons license and your intended use is not permitted by statutory regulation or exceeds the permitted use, you will need to obtain permission directly from the copyright holder.
To view a copy of this license, visit https://creativecommons.org/licenses/by/4.0/

Reviewer #1

Reviewer's comment:

This manuscript submitted by Chen, et al. presents a demonstration of a femtojoule-level all-optical Kerr switch operating at milliwatt peak power, based on a silicon-organic hybrid (SOH) photonic crystal slot nanobeam cavity. By combining small-mode-confinement, high Q resonances and MEH-PPV, the reported device achieves error-free 40 Gbit/s switching with 63 fJ/bit energy consumption, 7.5 mW peak power, and a contrast ratio of 8.3 dB. And, this scheme avoids the use of ultrashort, low-duty-cycle pulses. Overall, I think this manuscript is well organized, solid, and presents interesting technical merits. I expect that the authors can address the following points before my recommendation for publication in Nature Communications.

Author's reply:

We sincerely thank the reviewer for summarizing our work and giving positive feedback on the results. We are grateful that the reviewer finds our work to be well-organized, solid, and technically promising. We are committed to making the necessary revisions to address the reviewer's constructive suggestions and concerns in order to improve the manuscript further. Below, we provide detailed responses and modifications for each specific point raised.

Reviewer's Q1:

1. In introduction, the authors mentioned that to enhance the 3-rd nonlinearity for boosting Kerr effect, versatile photonic platforms have been investigated, that's true. Then the authors stated that silicon photonics is preferrable, due to its COMS compatibility. This flow is a bit strange. Since 3-rd nonlinearity and high Kerr effect is critical, silicon nitride cavities with pure Kerr oscillations should be a better choice. After all, as the authors wrote, TPA is a significant consideration in silicon, that is why the authors used SOH. In [eLight 4, 19 (2024)], comprehensive comparison of Kerr-effect in types of optical platforms have been provided. Referring the related reviews, I suggest the authors can find more fundamental motivations beyond the TPA and FCA. In addition, I suggest the authors can provide a table to discuss the performances and practicalities about the Kerr-switching on typical platforms.

Author's A1:

We sincerely thank the reviewer for this insightful comment and the constructive feedback. We understand the reviewer's concern regarding the flow of the introduction, specifically the discussion of third-order nonlinearity (Kerr effect) and the choice of silicon photonics as the preferred platform. In response, we would like to clarify the reasons why we emphasize silicon in the introduction, despite the advantages of silicon nitride and other platforms.

For most materials, the nonlinear refractive index approximately follows Miller's rule, increasing exponentially with the linear refractive index. However, this enhancement of Kerr nonlinearity is intrinsically accompanied by a reduction in bandgap energy, leading to pronounced two-photon absorption (TPA) and subsequent free-carrier absorption (FCA) at telecommunication wavelengths. As a result, material

selection for Kerr-based switching is fundamentally governed by a balance between nonlinear strength and nonlinear absorption.

Within this framework, silicon and silicon nitride represent two typical examples of this trade-off. Silicon offers a relatively large nonlinear refractive index ($n_2 = 4.5 \times 10^{-18}$ m^2/W) and strong optical confinement enabled by its high refractive index, allowing for compact device footprints and enhanced Kerr interaction. However, its performance is limited by TPA and FCA under high-intensity operation. In contrast, silicon nitride features a wide bandgap and negligible TPA at telecommunication wavelengths, but its significantly smaller nonlinear refractive index ($n_2 = 2.5 \times 10^{-19}$ m^2/W, approximately 18 times lower than silicon) necessitates either centimeter- to meter-scale waveguides or ultra-high-Q resonators to accumulate sufficient nonlinear phase shift. Such approaches, however, are generally incompatible with low-power and high-speed (>10–40 Gb/s) all-optical signal processing due to footprint, bandwidth, and energy-efficiency limitations.

To overcome these intrinsic limitations, we adopt a silicon-organic hybrid (SOH) platform. By integrating silicon with high-nonlinearity organic materials that deviate from conventional Miller scaling, the SOH configuration simultaneously leverages strong optical confinement from silicon and a large, low-loss Kerr response from the organic medium. This hybrid approach effectively balances nonlinearity, absorption, and optical confinement, thereby addressing the fundamental trade-offs that limit Kerr-effect-based switching in conventional material systems.

To better support this discussion, we have updated the platform discussion from a more fundamental material respect, with the Introduction part revised and a detailed material comparison added in the Supplementary Information, including a quantitative overview of refractive index, nonlinear refractive index, and bandgap energy across representative platforms.

(Line 60 in revised manuscript)

…To enhance weak $\chi^{(3)}$ optical nonlinearity, a wide array of integrated nonlinear photonic platforms has been extensively explored in recent years, including silicon [33–35], silicon nitride [36–38], lithium niobate [39,40], aluminium gallium arsenide [41], and silicon carbide [42]. However, the material selection for Kerr-based switching is fundamentally governed by a balance between nonlinear strength and nonlinear absorption [43]. This trade-off originates from the intrinsic correlation between the nonlinear refractive index and the linear refractive index, as described by empirical scaling relations following Miller's rule. As the refractive index increases, the nonlinear refractive index is typically enhanced, but this trend is also accompanied by a reduced bandgap energy, leading to strong two-photon absorption (TPA) and subsequent free-carrier absorption (FCA) at telecommunication wavelengths in high refractive index materials such as silicon [44–46]. To overcome these intrinsic limitations, the recently proposed silicon-organic hybrid (SOH) integration scheme has emerged as a promising alternative, attracting considerable attention [47,48]. This comes from the fact that organic nonlinear materials combine relatively low refractive index with exceptionally high nonlinear refractive index, thereby deviating significantly from the Miller scaling. By integrating such materials

into silicon slot waveguides, SOH designs enable ultra-tight transverse optical confinement within the TPA- and FCA-free ultra-high Kerr nonlinearity of the organic materials [49–51], by making use of the high refractive index of silicon while avoiding the nonlinear absorption of silicon. An additional advantage of the silicon platform is its native compatibility with complementary metal oxide semiconductor (CMOS) fabrication processes [52,53] (see extended discussions in Supplementary Information (SI) **S1**). …

…

[43] Yao, B. et al. Interdisciplinary advances in microcombs: bridging physics and information technology. *eLight* **4**, 19 (2024).

(Line 27 in revised Supplementary Information)

S1. Parameter Comparison of Material Platforms

Figure **S1** summarizes key material parameters of representative integrated photonic platforms, including the linear refractive index, nonlinear refractive index (n_2), and bandgap energy. In general, the relationship between the linear and the nonlinear refractive index approximately follows Miller's rule for most materials, i.e., the nonlinear refractive index increasing exponentially with the linear refractive index. However, this enhancement of Kerr nonlinearity is intrinsically accompanied by a reduction in bandgap energy, leading to pronounced two-photon absorption (TPA) and subsequent free-carrier absorption (FCA) at telecommunication wavelengths. As a result, material selection for Kerr-based switching is fundamentally governed by a balance between nonlinear strength and nonlinear absorption.

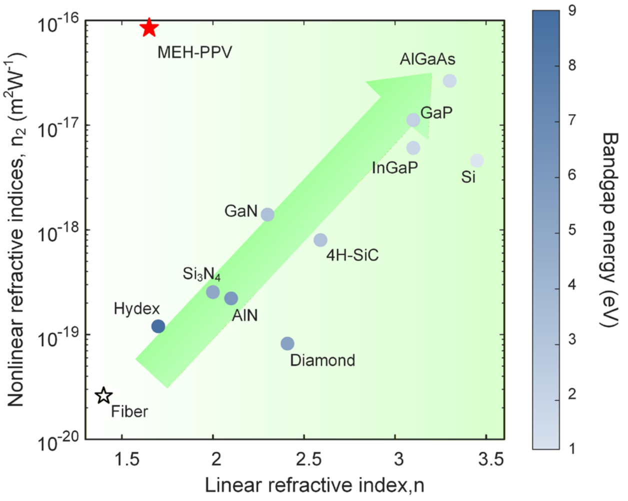

Fig. S1. Nonlinear refractive indices (n_2), bandgap energy, and linear refractive indices (n) of different material platforms.

Silicon and silicon nitride represent two typical examples of this trade-off. Silicon exhibits a relatively large nonlinear refractive index ($n_2 = 4.5 \times 10^{-18}$ m^2/W) and strong optical confinement owing to its high refractive index, enabling compact devices with enhanced Kerr interaction. Nevertheless, its

practical performance is severely constrained by TPA and FCA under high-intensity operation. In contrast, silicon nitride features a wide bandgap and negligible TPA at 1550 nm, but its significantly smaller nonlinear refractive index ($n_2 = 2.5 \times 10^{-19}$ m^2/W, approximately 18 times lower than silicon) necessitates centimetre- to meter-scale waveguides or ultra-high-Q resonators to accumulate sufficient nonlinear phase shift. Such approaches, however, are generally incompatible with low-power and high-speed (>10–40 GBaud) all-optical signal processing due to footprint, bandwidth, and energy-efficiency limitations. Notably, organic nonlinear materials (MEH-PPV) deviate from the conventional Miller scaling. Despite their relatively low linear refractive indices, they can exhibit exceptionally large Kerr nonlinearities ($n_2 = 8.5 \times 10^{-17}$ m^2/W, approximately 20 times that of silicon), while maintaining negligible nonlinear absorption in the telecommunication band. This unique combination enables a fundamentally different balance between nonlinearity and loss compared to inorganic materials.

By integrating high-nonlinear organic materials with silicon in a silicon-organic hybrid (SOH) configuration, the resulting platform simultaneously benefits from the strong optical confinement enabled by the high refractive index of silicon and the large, low-loss Kerr response of the organic polymer. It is important to note that the refractive index of slot structure play a critical role in the mode confinement of the switches. Figure **S2**(a) and (b) compares the mode confinement between two slot structures made of silicon and silicon nitride, respectively. It is clear that compared to silicon nitride, silicon slot geometry enables stronger concentrations of the optical field within the polymer region, substantially allowing more pronounced Kerr nonlinearity. This structure also elegantly relieves the impact of TPA and FCA in silicon since the mode intensity in the silicon region is small. Therefore, the SOH platforms effectively balance nonlinearity, absorption, and optical confinement—an intrinsic trade-off that constrains Kerr-effect-based switching in conventional material systems.

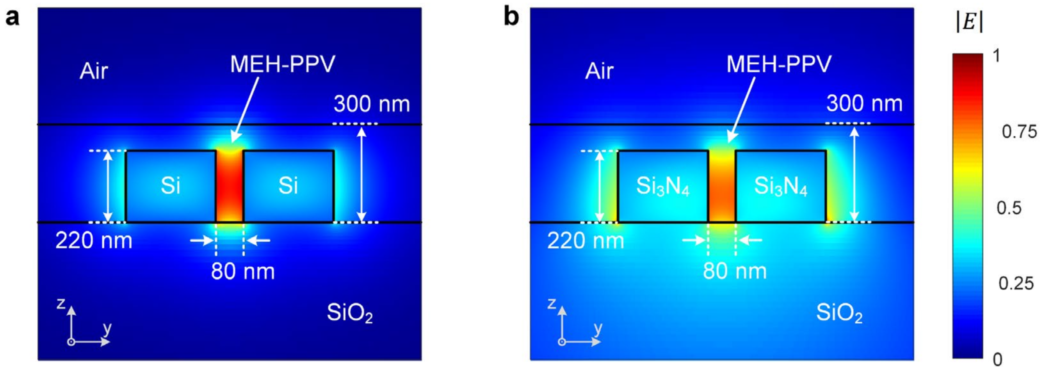

Fig. S2. Calculated electric field distributions ($|E|$) of silicon-organic hybrid integration (a) and silicon nitride-organic hybrid integration (b).

Reviewer's Q2:

2. In introduction, the authors claimed that "the majority of demonstrated devices rely on excitation by ultrashort pulses with Watt-level peak powers and extremely low duty cycles (defined as the ratio of the pulse duration to the repetition period, due to the intrinsically weak optical nonlinearity of available materials". I wonder that the intrinsically weak optical nonlinearity may be not the main limitation. For example, referring the literature [Nature Communications 13, 3138 (2022)], integrated 2D materials can offer high nonlinearity based-on chi(2) for achieving high-efficiency optical switching. I suggest the authors can broaden their discussions.

Author's A2:

We sincerely thank the reviewer for the insightful comment and agree that weak intrinsic nonlinearities represent only one aspect of the broader challenge. In response to the reviewer's suggestion, we have revised the introduction to broaden the discussion and clarify that this challenge is more fundamental and extends across material platforms. As now stated in the revised manuscript, even in systems that exhibit strong $\chi^{(2)}$ or $\chi^{(3)}$ nonlinearities (including emerging 2D-material-based platforms, lithium niobate, or topological insulators), it remains intrinsically difficult to simultaneously enhance the light-matter interaction while maintaining low optical loss. For instance, although 2D materials can provide exceptionally large nonlinear coefficients, efficient exploitation of their nonlinearity in integrated photonics typically requires extreme field confinement, often achieved via plasmonic or hybrid SPP structures, which inevitably introduce substantial insertion loss [*Nat. Photon.* **14**, 37–43 (2020)]. Lithium niobate offers strong $\chi^{(2)}$ nonlinearity; however its on-chip conversion efficiency is constrained by fabrication-induced scattering loss as well as stringent phase-matching requirements associated with periodic poling [*Nat. Photon.* **16**, 625–631 (2022)]. Similarly, while topological insulator-based platforms (e.g., Bi_2Se_3-on-Si) exhibit outstanding electrical and optical nonlinear properties, they also feature strong nonlinear absorption, which fundamentally limits their applicability for low-loss, high-speed all-optical switching [*Phys. Rev. Appl.* **14**, 044050 (2020)]. As a result, this trade-off—together with the limited nonlinear refractive index of many integrated materials—has forced most prior demonstrations to rely on ultrashort, high-peak-power excitation, which are far from ideal for practical all-optical signal processing.

(Line 34 in revised manuscript)

All-optical switches—a fundamental photonic building block for light-controlled operations—are essential to on-chip ultrafast optical interconnection networks [1–3], all-optical information processing [4–6], and photonic neural networks [7–10]. Their transformative potential lies in the ability to circumvent the bandwidth limitations inherent in conventional optoelectronic systems. A wide range of high-speed all-optical switches have been demonstrated, harnessing diverse fast optical nonlinearities, such as $\chi^{(2)}$ and $\chi^{(3)}$ nonlinearities [5,6,11–21], saturable absorption [22–25], and inter sub band transition [26,27]. …

(Line 42 in revised manuscript)

…However, the majority of demonstrated devices rely on excitation by ultrashort

pulses with Watt-level peak powers and extremely low duty cycles (defined as the ratio of the pulse duration to the repetition period). This reliance arises not only from the intrinsically weak nonlinearities of many integrated platforms, but also from the broader challenge of simultaneously enhancing light-matter interaction and maintaining low optical loss—even in materials that offer relatively strong nonlinearities [4,11,22,28]. While such low duty cycles help maintain low average power—thereby minimizing thermal effects and preventing device damage at elevated average power levels—they impose substantial limitations for practical implementations. …

…

[4] Wang. F. et al. All-optical mode-selective router based on broken anti-PT symmetry, *Phys. Rev. Appl*. **14**, 044050 (2020).

[11] Guo, Q. et al. Femtojoule femtosecond all-optical switching in lithium niobate nanophotonics. *Nat. Photon*. **16**, 625–631 (2022).

[20] Li, Y. et al. Nonlinear co-generation of graphene plasmons for optoelectronic logic operations. *Nat Commun.* **13**, 3138 (2022).

[21] Yao, B. et al. Gate-tunable frequency combs in graphene-nitride microresonators. *Nature* **558**, 410–414 (2018).

[22] Ono, M. et al. Ultrafast and energy-efficient all-optical switching with graphene-loaded deep-subwavelength plasmonic waveguides. *Nat. Photon*. **14**, 37–43 (2020).

Reviewer's Q3:

3. While the manuscript mentions compensating for thermo-optic effects via blue-shifting (Page 8) and highlights the high duty cycle (33%) as a key advantage, it lacks quantitative characterization of the temperature rise under sustained operation or long-term stability data. Given the high average power density inherent in cavity-enhanced structures and the known thermal sensitivity of polymers, this is a critical practical concern. I suggest the authors can add measurements or simulations estimating the temperature rise within the polymer-filled slot under continuous-wave (CW) pumping at the operational peak/average power levels. Or, the authors may consider to add data demonstrating stability over extended periods (e.g., minutes to hours) under the reported switching conditions (63 fJ/bit, 40 GB, 33% duty cycle). This could include monitoring BER, switching contrast, or resonance wavelength drift over time. Moreover, discussion on potential thermal management strategies if significant heating is observed. Related to other materials, e.g., silica microcavities [Phys. Rev. Letters 130(15), 153802 (2023)], I suppose the thermal effect may induces higher impact on Kerr-nonlinearity in the SOH structure.

Author's A3:

We sincerely thank the reviewer for raising this important and practical concern regarding the potential role of thermal effects and long-term stability in hybrid silicon-organic (SOH) nanocavity devices operating at high duty cycle.

1. **Quantitative estimation of thermal contribution**

In hybrid silicon-organic nanocavity devices, optical absorption can in principle give rise to local heating, which may induce refractive index changes through the thermo-optic effect. To quantitatively assess the possible thermal contribution, we estimate the local temperature rise induced by optical absorption under the experimental operating conditions. A detailed analysis is provided in Section **S8** of the revised Supplementary Information.

Under continuous-wave excitation, the resonance wavelength exhibits a power-dependent shift arising from the combined contributions of Kerr nonlinearity and thermo-optic effects. By quantitatively accounting for the Kerr-induced refractive index change using the independently calibrated nonlinear coefficient, we separate the residual thermal contribution and obtain an effective thermal tuning efficiency of approximately -0.434 nm/mW for the hybrid nanocavity. At the experimentally used operating point in the manuscript (average on-chip pump power 1.26 mW for 63 fJ/bit at 40 GBaud and 33% duty cycle), the corresponding steady-state temperature rise is estimated to be approximately 5.9 ℃, using reported thermo-optic coefficients of $dn/dT \approx -1 \times 10^{-4}\ \mathrm{K}^{-1}$ for MEH-PPV [*Polymer (Guildf)*. **47**, 4893–6 (2006)]. At the low-power operating conditions employed in this work, the estimated temperature rise is modest and will not induce material degradation or device instability (provided in the box below). Moreover, under high-speed PRBS excitation, where the bit period (25 ps at 40 GBaud) is several orders of magnitude shorter than the thermal response time of the nanocavity (microsecond scale), the temperature evolution is governed solely by the average absorbed power and remains quasi-static during bit-level modulation. Consequently, thermal effects can at most introduce a static resonance offset, which is compensated experimentally by wavelength pre-biasing, and cannot follow the instantaneous pump envelope.

(Line 449 in revised Supplementary Information)

…Figure **S16a** shows the transmission spectrum of the new samples without pumping, featuring three distinct resonances corresponding to Fano mode 1, 2 and 3. Under continuous-wave excitation, the resonance wavelength exhibits a clear power-dependent shift arising from the combined action of the Kerr nonlinearity and thermally induced refractive index changes. As shown in Fig. **S16b**, when the pump wavelength is tuned to Fano mode 3, a clear blueshift of mode 1 is observed with increasing input pump power. To quantitatively separate the Kerr and thermal contributions, we first calculate the Kerr-induced refractive index change using the temporal coupled-mode theory (TCMT) model described in Section **S2**, together with the independently calibrated nonlinear coefficient of the polymer. Subsequently, the remaining contribution, obtained by subtracting the Kerr-induced shift from the experimentally measured total shift, can therefore be attributed to thermal effects, as summarized by the red scatter points in Fig. **S17**. From this analysis, we extract an effective thermal tuning efficiency of approximately $d\lambda/dP \approx -0.434\ \mathrm{nm/mW}$. Using the experimentally extracted thermal tuning efficiency, we estimate the steady-state temperature rise at the operating point used in the main switching experiments. At the saturation threshold reported in the main text (switching energy of 63 fJ/bit), the corresponding average on-chip pump power is approximately 1.26 mW. This

yields an estimated steady-state temperature increase of approximately $\Delta T \approx 5.9$ °C, using the reported thermo-optic coefficient of MEH-PPV ($dn/dT \approx -1 \times 10^{-4}\ \mathrm{K}^{-1}$) [12,13].

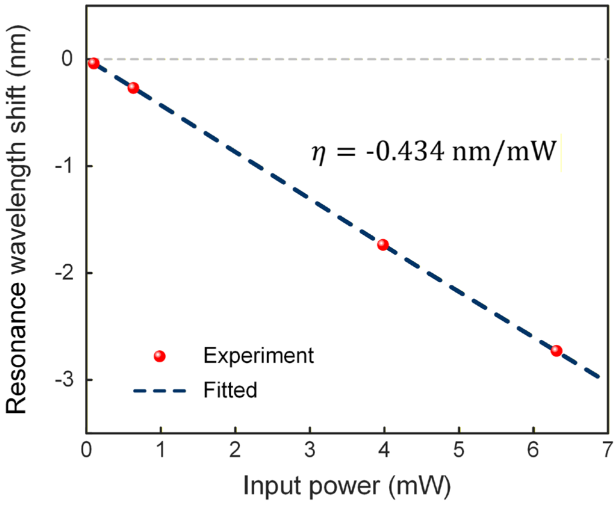

Fig. S17. Resonance wavelength shifts caused by the thermal effect at different input powers.

Additionally, to evaluate the thermal stability of the polymer material (MEH-PPV), we perform complementary thermal analysis using thermogravimetric analysis (TGA) and differential scanning calorimetry (DSC). TGA is conducted in a nitrogen atmosphere to measure the thermal decomposition point of MEH-PPV. As shown in Fig. **S18**, we test different samples: samples 1-3 are MEH-PPV films of varying weight, prepared by dissolving MEH-PPV powder in toluene, depositing it onto substrates, and drying them to obtain films of approximately 26 μm thickness; Sample 4 is the original solid MEH-PPV powder. The TGA results indicate that the material begins to decompose around 350 °C, which is significantly higher than the operating temperatures used in our experiments, suggesting that thermal degradation under typical experimental conditions is unlikely to occur.

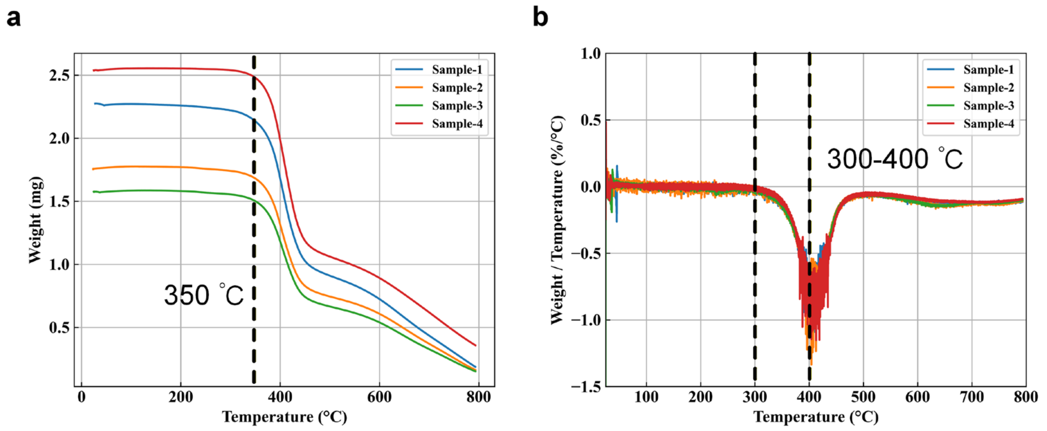

Fig. S18. Thermogravimetric analysis (TGA) of MEH-PPV samples in a nitrogen atmosphere.

DSC measurements are carried out under nitrogen atmosphere to evaluate the melting point and thermal response of the material. As shown in Fig. **S19**(a) and (b), no prominent peaks associated with melting are observed during both the first heating and second heating, indicating that the melting point of MEH-PPV is above 320 °C (positive heat flow corresponds to exothermic (heat-releasing) processes, while negative heat flow corresponds to endothermic (heat-absorbing) processes). Additionally, based on previous TGA data, it is likely that the polymer begins to decompose before it melts. The first peak observed in the DSC measurement corresponds to the thermal balance of the system and is independent of the material itself. These results further confirm the thermal stability of MEH-PPV, ensuring that thermal effects under operational conditions do not significantly contribute to performance degradation. For future scaling toward higher power handling or broader operating scenarios, the thermal robustness of the nonlinear polymer can be further enhanced through materials and process engineering. Building on recent advances in thermally stable and crosslinked organic polymer systems [14], the nonlinear polymer may be spin-coated in combination with an imprint resist or cross-linkable host matrix, followed by ultraviolet curing. Such approaches have been shown to increase the glass transition temperature and suppress thermally induced polymer reflow, thereby providing a viable pathway for improving the thermal tolerance of hybrid silicon-organic nanophotonic devices.

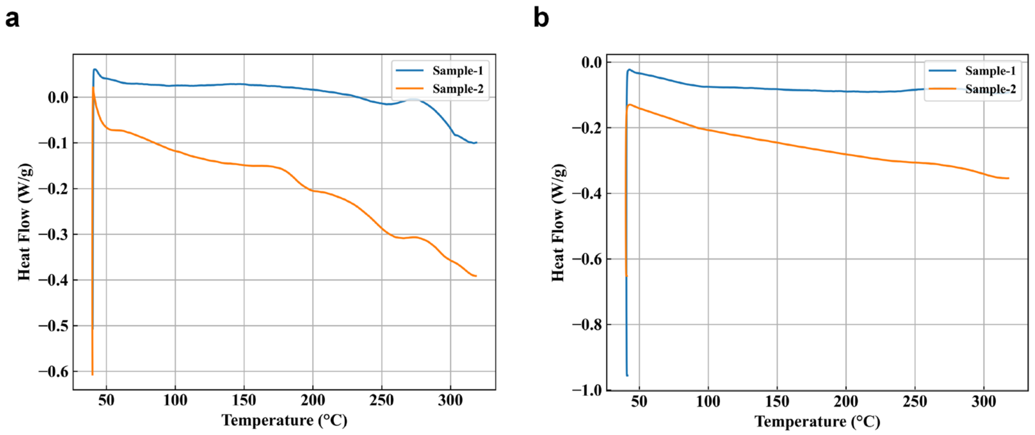

Fig. S19. Differential scanning calorimetry (DSC) of MEH-PPV samples under nitrogen atmosphere during the first heating (a) and second heating (b).

Notably, the extracted thermal tuning efficiency exhibits a negative sign, which is consistent with the reported negative thermo-optic coefficient of the polymer MEH-PPV ($dn/dT < 0$), and opposite to the positive thermo-optic coefficient of silicon ($dn/dT \approx 1.85 \times 10^{-4}\ \mathrm{K}^{-1}$). This observation indicates that the thermally induced resonance shift is dominated by the polymer-filled slot region, rather than by the surrounding silicon rails. Therefore, this thermally induced refractive index reduction partially offsets the Kerr-induced redshift, thereby degrading the overall signal quality. …Under high-speed PRBS excitation, where the bit period (25 ps at 40 GBaud) is several orders of magnitude shorter than the thermal response time of the nanocavity, this temperature rise is governed solely by the average optical power

of the pump ($\Delta n_{thermal} \propto P_{AVG}$) and remains quasi-static during bit-level modulation. To mitigate this effect, a pre-shift of the input wavelength can be applied, in which the input pump wavelength is slowly scanned from the red-detuned side toward the blue side until the cavity reaches a thermally stabilized operating point [15]. Once thermal equilibrium is established at the target average power, the high-speed modulation is applied, effectively compensating the negative thermo-optic drift and stabilizing the cavity response during ultrafast signal modulation.

…

[13] Zhang, Z. et al. Thermo-optic coefficients of polymers for optical waveguide applications. *Polymer (Guildf)*. **47**, 4893–6 (2006).

[14] Xu, H. et al. Ultrahigh Performance Cross-Linkable Organic Electro-Optic Material for Hybrid Modulators. *Chemistry of Materials* **37**, 12 (2025).

[15] Zhang, H. et al. Soliton Microcombs Multiplexing Using Intracavity-Stimulated Brillouin Lasers. *Phys. Rev. Lett*. **130**, 153802 (2023).

2. Long-term stability under sustained high-speed operation

To address concerns regarding practical stability under sustained operation, we further investigate the device performance over extended time durations under the reported switching conditions, without employing any feedback control or external temperature stabilization. Specifically, the device is continuously operated for 30 minutes at 40 GBaud, 63 fJ/bit, and 33% duty cycle, while monitoring the key performance metric of bit-error rate (BER). No measurable performance degradation or increase in error rate is observed throughout the measurement duration, confirming that the modest, quasi-static temperature rise does not compromise the operational stability of the device under realistic high-speed data-stream excitation. A detailed analysis is provided in Section **S11** of the revised Supplementary Information.

For future scaling toward longer-term deployment or higher power operation, additional stabilization strategies can be readily implemented. These include active feedback control of the pump wavelength and power or chip temperature, as well as enhanced thermal management enabled by optimized packaging and heat dissipation techniques. Furthermore, the thermal robustness of the nonlinear polymer can be substantially improved through materials engineering approaches, such as incorporating cross-linkable polymer or imprinted resist matrices followed by ultraviolet (UV) curing. Related studies have shown that such crosslinked organic polymer systems exhibit significantly enhanced thermal stability and elevated glass transition temperatures at the material level [*Chemistry of Materials* **37**, 12 (2025)], offering a viable pathway toward improving the thermal tolerance of hybrid silicon–organic nanophotonic devices.

(Line 577 in revised Supplementary Information)

S11. Long-term stability measurements

To further evaluate the operational stability under sustained high-speed switching, a long-term stability measurement is performed under fixed operating conditions corresponding to the saturation threshold reported in the main text, without employing any feedback control or external temperature stabilization. Specifically, the device is continuously operated at a switching energy of 63 fJ/bit, a signal rate of

40 GBaud, and a duty cycle of 33% for a duration of 30 minutes. During the measurement, the received optical power at the photodetector (PD) is fixed at -1.8 dBm, and the bit-error rate (BER) is continuously monitored using the experimental setup described in Fig. **3a**. The measured BER remains below 10^{-9} throughout the entire 30-minute interval, confirming sustained error-free operation, as shown in Fig. **S23**. During the first ~20 minutes, the BER is essentially constant, indicating stable switching behavior under steady-state thermal conditions. In the subsequent ~10 minutes, a slight increase in the measured BER is observed, which is attributed primarily to slow fluctuations in the grating-coupler coupling efficiency and minor cavity detuning. Importantly, the BER remains well below the error-free threshold, and no abrupt degradation or instability is observed. These results demonstrate that, under the reported operating conditions, the modest and quasi-static temperature rise does not compromise the long-term stability of the all-optical switching performance. For future implementations targeting extended deployment times or more demanding environments, additional stabilization strategies—such as active feedback control of the pump wavelength or optical power, chip-level temperature control, and optimized device packaging—can be readily incorporated to further enhance system robustness.

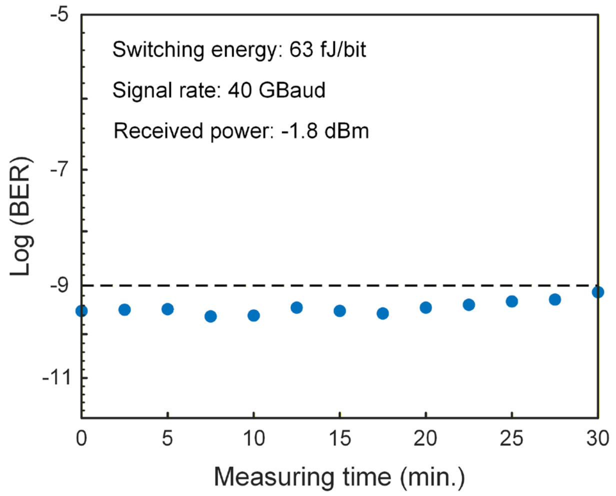

Fig. S23. BER stability measurement over 30 minutes under continuous 40-GBaud switching operation.

In summary, both quantitative thermal estimation and sustained-operation measurements demonstrate that, under the operating conditions employed in this work, the temperature rise induced by optical absorption in the hybrid nanocavity remains modest and does not impose a practical limitation on device performance. Thermal effects are governed by the average optical power and remain quasi-static during high-speed modulation, allowing effective compensation through wavelength pre-biasing. The demonstrated stable operation under high-duty-cycle, high-speed switching conditions confirms the robustness and practicality of the proposed silicon-organic nanocavity platform for ultrafast all-optical signal processing.

Reviewer's Q4:

4. The manuscript briefly mentions the potential for multi-channel operation via WDM in the Discussion (Page 10, Lines 276-278) as a route to Tbps processing. However, no experimental evidence or quantitative analysis supporting this claim is provided. To substantiate this claim and strengthen the impact, the authors may experimentally demonstrate simultaneous switching of at least two independent probe channels (different wavelengths) using a single pump channel on the same device structure, and/or discuss the practical limitations on channel count and spacing imposed by the cavity design (Q-factor, resonance spacing, mode overlap) and nonlinear material bandwidth.

Author's A4:

We sincerely thank the reviewer for this important comment regarding the claim about multi-channel operation via Wavelength Division Multiplexing (WDM) and the potential for Terabits per second (Tbps) processing. In the original manuscript, we intended to describe the potential for multi-channel switching through the design of a series of cascaded nanocavities, each with a distinct resonance wavelength, as shown in Fig. **R1**, rather than relying on multiple resonant peaks within a single device structure. This approach enables the simultaneous switching of multiple optical signals across different wavelengths using WDM techniques.

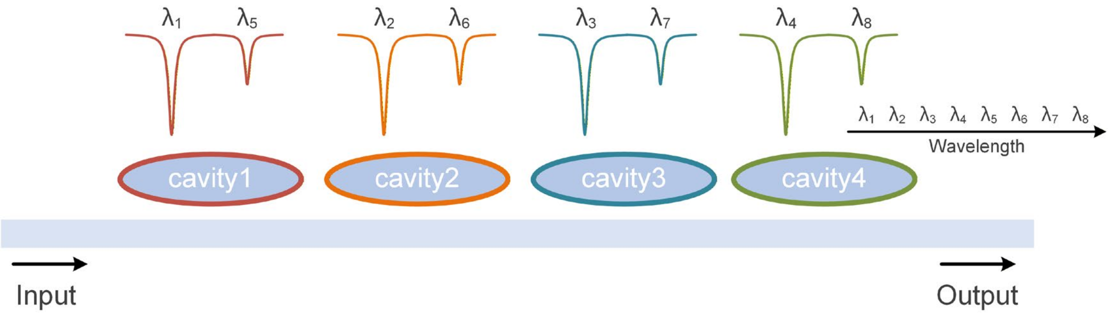

Fig. R1. Schematic diagram of the multi-channel SOH Kerr all-optical switch.

To further substantiate this argument, we have conducted additional experiments to demonstrate the multi-channel switching capability within the cascaded cavity structure. The transmission spectrum of the device is shown in Fig. **R2(a)**, where each cavity supports two spectrally separated modes, giving rise to paired resonances in the mode 1 and mode 2 groups with a wavelength separation of approximately 30 nm. Figure **R2(b)** presents the time-domain response of probe light from different channels, as measured using a CSA. The solid red and blue lines correspond to the original pump signal and the modulated probe output from channels 1 to 4, respectively. The overall quality of the output probe signals is exceptionally high, demonstrating the excellent performance of device in multi-channel switching.

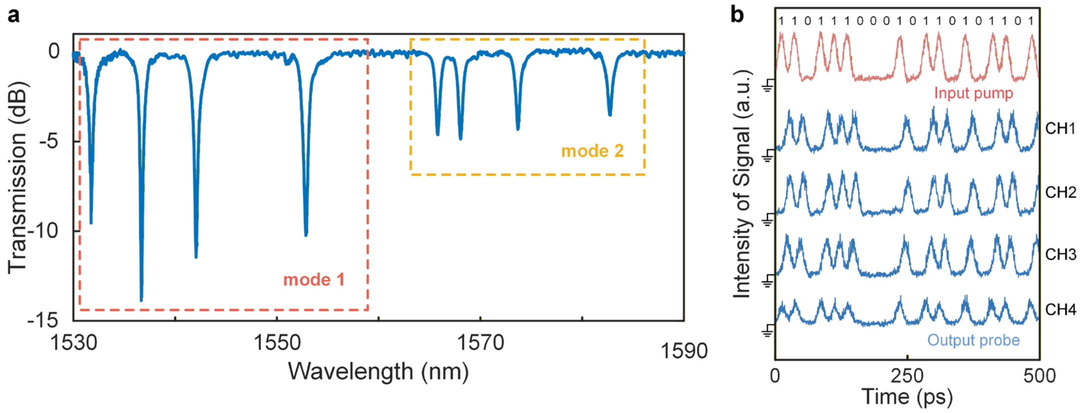

Fig. R2. 4×40 GBaud all-optical switching. a Experimental measured transmission spectrum. **b** Input pump pulse train (red) and output probe pulse train from different channels (blue).

From the spectrum in Fig. **R2(a)**, it is evident that the current 4-channel configuration is far from utilizing the entire spectral bandwidth. Therefore, by cascading additional nanobeam cavities with distinct resonant wavelengths, the WDM capability can be further leveraged to enable each cavity to independently achieve femtojoule-level energy switching at 40 GBaud rates. For such an integrated design, while maintaining the high Q-factors and strong cavity-waveguide coupling required for efficient single-cavity switching, the cavity length should be minimized to increase the spectral spacing between resonances, thereby enabling a larger number of parallel channels without spectral overlap. Assuming a single resonance linewidth of approximately 0.6 nm, a wavelength separation of about 30 nm would in principle accommodate about 25 distinct cavity resonances, potentially facilitating Tbps-level (25 × 40 GBaud) signal processing. To avoid ambiguity for readers, we have revised the main text and provided a more detailed explanation in the Discussion section.

(Line 297 in revised manuscript)

…By designing a series of cascaded nanocavities with distinct resonances, optical signal switching can be simultaneously achieved across multiple channels via wavelength-division multiplexing (WDM) techniques, rendering potentials for Terabits/s (Tbps) information processing [69]. For such an integrated design, while maintaining the high Q-factors and strong cavity-waveguide coupling required for efficient single-cavity switching, the cavity length should be minimized to increase the spectral spacing between resonances, thereby enabling a larger number of parallel channels without spectral overlap.

Reviewer's Q5:

5. The authors utilized MEH-PPV, a conjugated polymer known to be susceptible to photodegradation (photo-oxidation) under illumination, especially at telecom wavelengths involving higher photon energies than its bandgap. The high intracavity field intensity and sustained high-repetition-rate operation could accelerate this degradation, impacting device lifetime and reliability – a crucial factor for practical

applications. I suggest the authors can showcase accelerated lifetime testing under operational optical intensities (peak and average power) relevant to their switching conditions. And, they can include a statement on the current observed operational stability within the timeframe of their reported experiments. For instance, in previous studies, such as [Nature 558, 410 (2018)], the existence of intracavity polymer may decelerate the photon-electron interactions especially when changing the status of Kerr oscillation.

Author's A5:

We sincerely thank the reviewer for raising this important point regarding the photostability and long-term reliability of MEH-PPV under high intracavity field intensity and high-repetition-rate operation, which is indeed a critical consideration for practical deployment of hybrid silicon-organic nanophotonic devices.

We agree that accelerated lifetime testing under elevated optical conditions is a valuable approach for quantitatively assessing long-term photodegradation. Such tests typically rely on operating devices at optical intensities substantially exceeding their nominal operating conditions [*NPL Report DEPC MPR* **016** (2005)]. However, in the present device architecture, the switching operation critically depends on precise spectral alignment between the pump wavelength and the cavity resonance. Excessive increase in the pump power would induce strong self-phase modulation (SPM), which dynamically detunes the pump from the cavity resonance and suppresses the intended switching operation. As a result, performing conventional accelerated lifetime testing by simply increasing the optical power is experimentally nontrivial for this Kerr-resonant switching scheme.

Nevertheless, we conduct lifetime testing on a 10 mm×10 mm MEH-PPV film (quartz substrate) to further investigate the material photostability under sustained illumination. Firstly, we perform Z-scan measurements with a minimal input power, corresponding to a focal intensity of approximately 29 GW/cm², to determine the nonlinear refractive index (n_2) and nonlinear absorption of the polymer. The Z-scan setup utilize femtosecond pulses at 1550 nm, generated by an optical parametric amplifier (OPA) from a 1030 nm source. Closed-aperture scans are conducted to extract the nonlinear refractive index, while open-aperture scans are performed simultaneously to assess nonlinear absorption. In the closed-aperture configuration, we observe a clear valley-peak feature, confirming the presence of positive Kerr nonlinearity (self-focusing), as shown in Fig. **R4(a), R5(a)** and **R6(a)**. The measured n_2 values at the three points are $3.09 \times 10^{-16}\ \mathrm{m^2/W}$, $1.93 \times 10^{-16}\ \mathrm{m^2/W}$, and $9.22 \times 10^{-17}\ \mathrm{m^2/W}$, respectively. The differences in these values are primarily due to the non-uniform thickness of the film and potentially minor errors introduced by the low test-power. In the open-aperture scan, the normalized power remains flat, indicating negligible nonlinear absorption as shown in Fig. **R7**. Subsequently, we progressively increase the laser power, with focal intensities raised from 29 GW/cm² to 34 GW/cm², 39 GW/cm², 44 GW/cm², and 49 GW/cm², maintaining continuous illumination for 30 minutes followed by 10 minutes of shading treatment. After each irradiation, n_2 measurements are repeated under the initial conditions (29 GW/cm²), as shown in Fig. **R4, R5** and **R6**. The total duration of lifetime testing is 2 hours. The results, presented in Fig. **R3**, clearly

demonstrate a noticeable decrease in n_2 as the power increases, with n_2 remaining consistently above $6.81 \times 10^{-17}\ \mathrm{m^2/W}$. Notably, n_2 remained approximately 15 times that of silicon ($4.5 \times 10^{-18}\ \mathrm{m^2/W}$), indicating that the nonlinear refractive index of polymer maintains a high level even after prolonged exposure to high-power illumination.

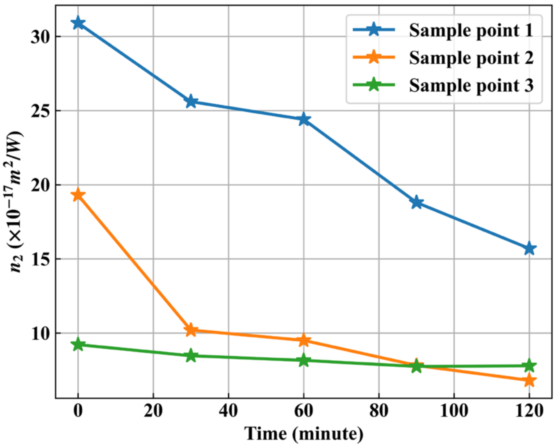

Fig. R3. Statistical analysis of the nonlinear refractive index n_2 of the polymer under different light intensities after 30 minutes of illumination.

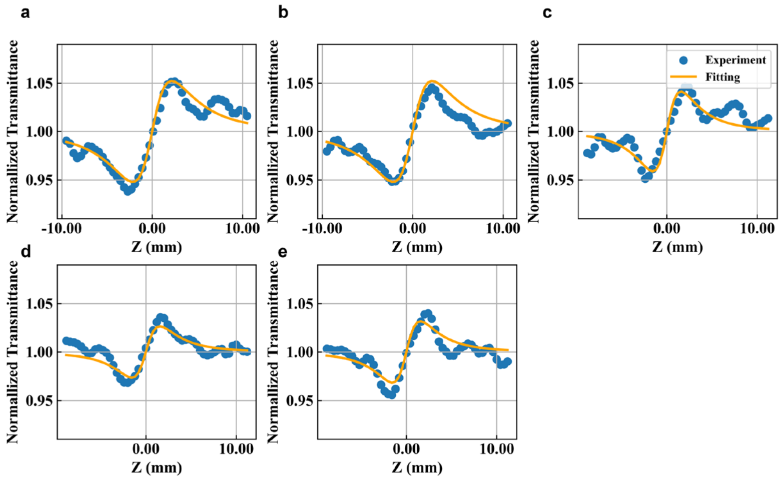

Fig. R4. Z-scan measurement of the nonlinear refractive index n_2 of Sample point 1 in the closed aperture configuration. The initial state measurement is performed under an illumination intensity of 29 GW/cm² **(a)**. Subsequently, the material is illuminated for 30 minutes with intensities of 34 GW/cm² **(b)**, 39 GW/cm² **(c)**, 44 GW/cm² **(d)**, and 49 GW/cm² **(e)**, and the results are tested using the initial conditions (29 GW/cm²).

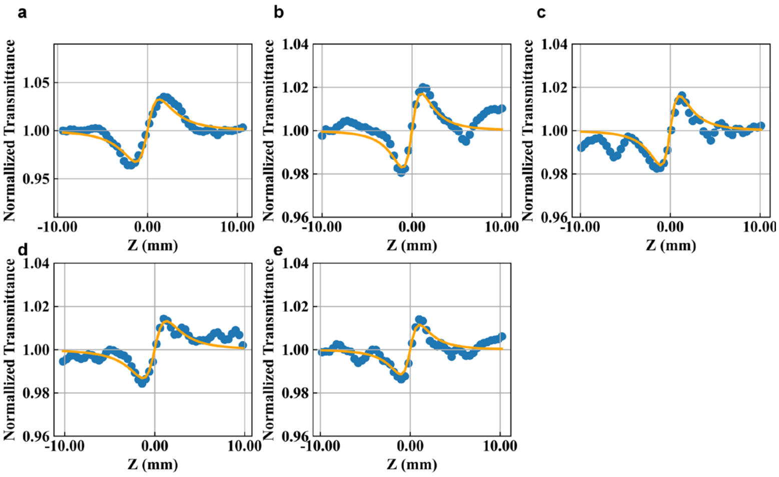

Fig. R5. Z-scan measurement of the nonlinear refractive index n_2 of Sample point 2 in the closed aperture configuration. The initial state measurement is performed under an illumination intensity of 29 GW/cm² **(a)**. Subsequently, the material is illuminated for 30 minutes with intensities of 34 GW/cm² **(b)**, 39 GW/cm² **(c)**, 44 GW/cm² **(d)**, and 49 GW/cm² **(e)**, and the results are tested using the initial conditions (29 GW/cm²).

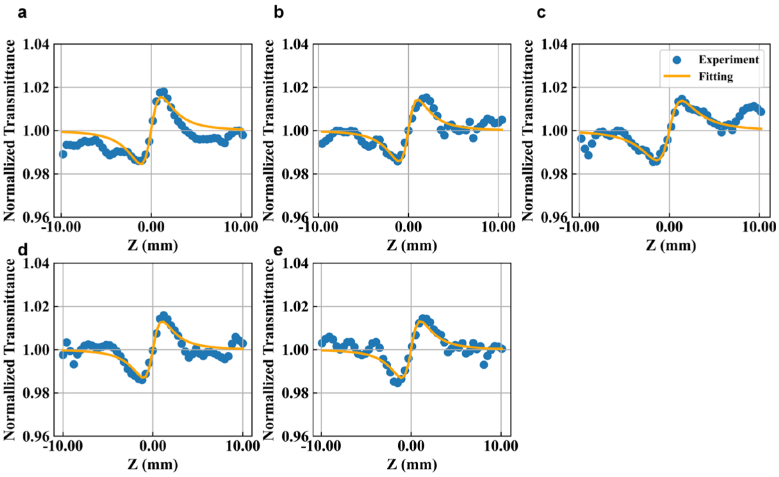

Fig. R6. Z-scan measurement of the nonlinear refractive index n_2 of Sample point 3 in the closed aperture configuration. The initial state measurement is performed under an illumination intensity of 29 GW/cm² **(a)**. Subsequently, the material is illuminated for 30 minutes with intensities of 34 GW/cm² **(b)**, 39 GW/cm² **(c)**, 44 GW/cm² **(d)**, and 49 GW/cm² **(e)**, and the results are tested using the initial conditions (29 GW/cm²).

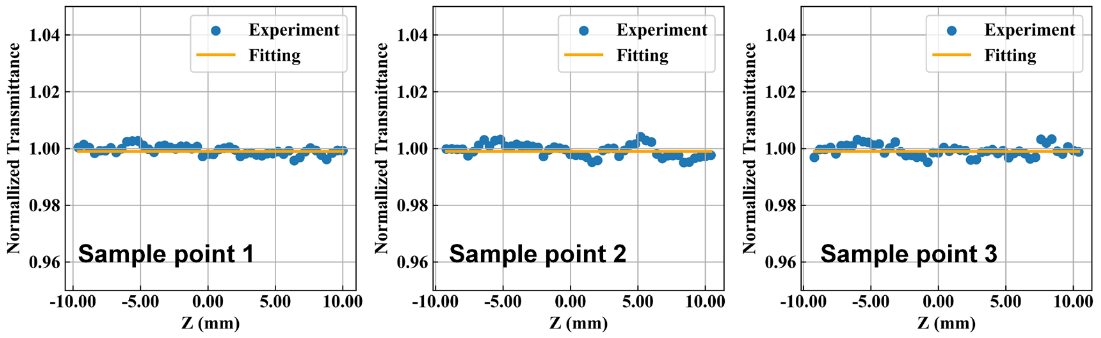

Fig. R7. Open aperture Z-scan measurement of the MEH-PPV film for absorption characterization.

Additionally, within the experimentally accessible and application-relevant operating regime, we have nevertheless performed continuous-operation stability measurements under the reported switching conditions (40 GBaud, 63 fJ/bit, 33% duty cycle). As presented in **Author's A3** and Supplementary Section **S11**, the device is operated continuously for 30 minutes while monitoring the bit-error rate (BER). Throughout the entire measurement duration, no sudden BER degradation, performance drift, and instability or irreversible phenomena are observed. These results indicate that neither the high intracavity field intensity nor the sustained high-repetition-rate operation induces observable photodegradation or performance deterioration on this timescale. Consequently, the device demonstrates stable and reliable operation within the experimentally relevant timeframe and operating conditions reported in this study.

Taken together, these thermal analyses, Z-scan measurements, in addition to the 30-minute stability testing, demonstrate that MEH-PPV exhibits excellent thermal and optical stability, making it a suitable material for high-speed, high-duty-cycle all-optical switching applications.

Reviewer's Q6:

6. In Fig. 3a, the output probe power appears to be relatively low. Are optical amplifiers used before detection? Given common practice in similar experiments, the authors likely employed a filter–amplify–filter scheme prior to detection. However, the figure omits any depiction of amplification components. The absence of this information may lead to confusion for readers attempting to interpret the results or replicate the setup. I suggest adding a brief explanatory note in the figure caption, possibly with relevant references to similar implementations. In addition, one of the merits of using XPM for switching, the output power is relatively higher since it employs interference effect, as comparing to FWM effect that generates new frequency component. I suggest the authors to clarify the reason for low output power of their results and discuss possible ways to further improve the results.

Author's A6:

We sincerely thank the reviewer for raising these important points.

1. Experimental amplification and filtering chain

As the reviewer correctly noted, the output probe power in Fig. **3a** appears to be relatively low. We did indeed employ an erbium-doped fiber amplifier (EDFA), and we used the filter-amplify-filter scheme before detection, as is common practice in similar experiments. In the experiment, the probe input power is approximately -5 dBm, which is much lower than the pump power, ensuring that the nonlinear Kerr phase shift is predominantly induced by the pump. At the output, the detected probe signal is filtered out by a bandpass filter, resulting in an output power of approximately -23 dBm, which is then amplified by an EDFA. A second bandpass filter is employed to suppress amplified spontaneous emission noise and further improve signal quality. We have updated the experimental setup as well as the figure caption to include this information.

(Line 506 in revised manuscript)

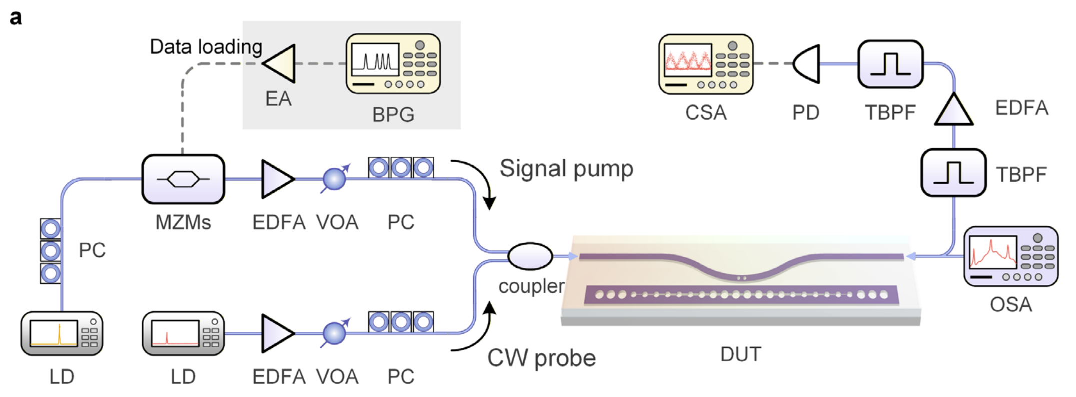

Fig. 3. Demonstration of two-mode all-optical Kerr switching. **a** Experimental setup for the all optical switching measurement. LD: laser device; PC: polarization controller; MZMs: Mach Zehnder modulators; BPG: bit pattern generator; EA: electrical amplifier; EDFA: erbium-doped fiber amplifier; VOA: variable optical attenuator; DUT: device under test; TBPF: tunable bandpass filter; PD: Photodiode; CSA: communication signal analyzer; OSA: optical spectrum analyzer. An EDFA is used to amplify the output probe signal [67]. …

…

[67] Gu, W. et al. All-optical complex-valued convolution based on four-wave mixing. *Optica* **11**, 64-72 (2024).

2. Clarification of the observed low probe output power

As the reviewer correctly pointed out, cross-phase modulation (XPM) based on the Kerr effect exhibits relatively high output power since it employs interference effect, whereas four-wave mixing (FWM) generates new frequency components. In our experiments, the on-chip probe power after switching remains comparable to the input continuous-wave (CW) probe power, indicating that the XPM process itself does not intrinsically result in significant power loss. The relatively low optical power measured at the detector can be attributed to practical experimental constraints and device-related losses rather than an inefficient Kerr-based switching mechanism. Specifically, the input probe power is intentionally kept at a modest level to avoid introducing additional Kerr-induced phase shifts from the probe itself, which could otherwise perturb the switching dynamics. This limitation is not fundamental and can be adjusted in future

experiments with appropriate compensation strategies or optimized operating schemes, potentially allowing higher probe powers. Further, the detected power is reduced by extrinsic coupling and insertion losses. In particular, the photonic crystal (PhC) grating couplers contribute approximately -6 dB/facet. The corresponding SEM images and measured transmission spectrum of the grating couplers in Section **S7** of the revised Supplementary Information. This relatively high coupling loss mainly arises from the difficulty of uniformly filling the PhC air holes with polymer. In addition, the device insertion loss (~2-4 dB, see Fig. **2**) is primarily attributed to mode mismatch between the bus waveguide and the slot PhC nanobeam cavity. These losses are not intrinsic to the switching principle and can be substantially reduced through improved mode matching, optimized grating designs, or by adopting end-fire coupling schemes instead of vertical grating couplers. With such improvements, significantly higher output probe power can be expected in future implementations.

(Line 421 in revised Supplementary Information)

…

In the experiment, light is coupled into and out of the chip using photonic crystal (PhC) grating couplers, with a measured coupling loss of approximately -6 dB/facet, as shown in Fig. **S15**. The intrinsic on-chip insertion loss (*IL*) of the device, excluding grating-coupler losses, is approximately -2 dB to -4 dB (as shown in Fig. **2**), which mainly originates from mode mismatch between the bus waveguide and the slot PhC nanobeam cavity. In future implementations, the overall optical throughput could be improved by optimizing the coupling efficiency and reducing the intrinsic insertion loss, for example through improved mode matching or by adopting edge-coupling schemes instead of vertical grating couplers.

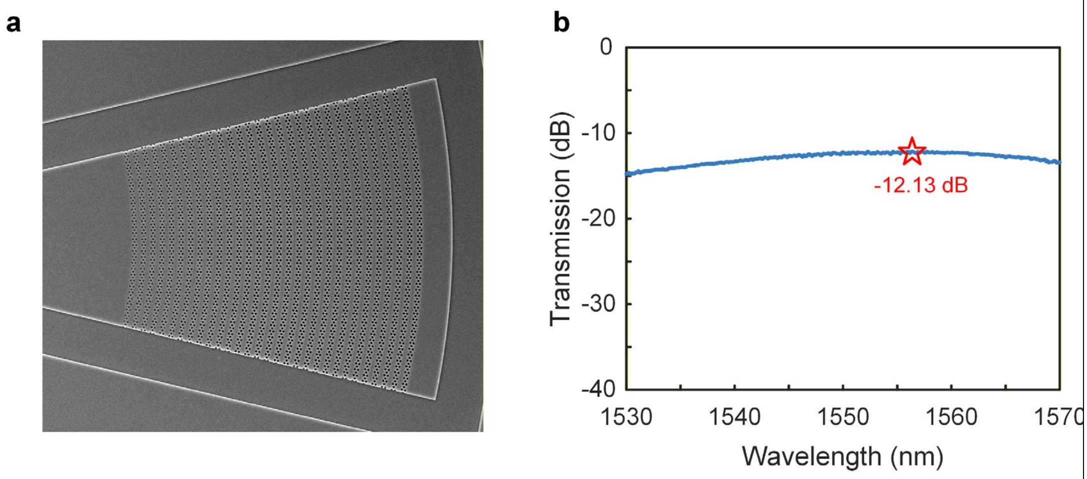

Fig. S15. SEM images and transmission spectrum of the grating coupler.

Reviewer's Q7:

7. While the eye diagrams in Fig. 4a demonstrate reasonably clear openings—with some thickening of the upper eyelid but otherwise good eye clarity and amplitude—it is regrettable that the manuscript provides only a subjective statement: "the eye diagram displays high signal quality." I suggest that the authors offer a more quantitative and

rigorous description of the eye diagram quality, potentially by including standard metrics such as eye height, eye width or Q-factor to support the visual assessment.

Author's A7:

We sincerely thank the reviewer for this constructive suggestion. We agree that a more quantitative and rigorous assessment of the eye diagram quality would improve the manuscript. In response to the reviewer's comment, we have supplemented the description of Fig. **4a** with a detailed quantitative analysis of the eye diagram, including the Q value of the eye diagram and Signal-to-Noise Ratio (SNR) [*International Conference on Transparent Optical Networks (ICTON)*, 1-4 (2012)]. Among these, the Q value of the eye diagram is defined as the ratio of the signal amplitude to the standard deviation of the noise. A higher the Q value indicates a better signal quality with less overlap between adjacent symbols. The SNR is defined as the ratio of the signal power to the noise power, providing an indication of the clarity of the received signal relative to the noise level. Higher SNR values correspond to better signal clarity and lower likelihood of errors. We believe these additions provide a more objective and comprehensive evaluation of the eye diagram quality, as well as a clearer understanding of the signal quality in our system.

(Line 243 in revised manuscript)

…The switching contrast achieves 5.8 dB at 40 fJ/bit and a peak power of 4.8 mW, with the eye diagram exhibiting a thick upper eyelid (inset i), which corresponds to a Q value of 2.3 and a Signal-to-Noise Ratio (SNR) of 7.2 dB. Here, the Q value of the eye diagram is defined as the ratio of the signal amplitude to the standard deviation of the noise, while the SNR is defined as the ratio of the signal power to the noise power [68]. As the switching energy increases, the switching contrast continues to improve, reaching 8.3 dB at 63 fJ/bit and a peak power of 7.5 mW. The eye diagram at this point (inset ii) demonstrates high signal quality, with a Q value of 3.4 and an SNR of 10.5 dB, reflecting a significant improvement in both signal clarity and noise margin. At this stage, the system reaches the saturation threshold, where further increases in energy no longer result in substantial improvements. As shown in inset iii, both the switching contrast and the eye diagram quality become saturated, where the Q value is approximately 3.6 and the SNR is 11.1 dB. This is because once the pump energy is strong enough to shift the probe light completely out of the resonance window, further increase in pump energy has little impact on the switching contrast. The overall trend of the experimental results aligns well with the theoretical predictions based on nonlinear time-domain coupled-mode equations (see SI-S2 for details).

…

[68] Freude, W. et al. Quality metrics for optical signals: Eye diagram, Q-factor, OSNR, EVM and BER. *International Conference on Transparent Optical Networks (ICTON)*, 1-4 (2012).

Reviewer's Q8:

8. A variety of chip-scale all-optical switches has been demonstrated across different material platforms and nonlinear mechanisms. In Fig. 5, the authors provide a

comprehensive and intuitive comparison between their device and several representative approaches, including those based on free-carrier effects, Kerr nonlinearity, chi(2) effects, and saturable absorption. While this type of comparison is valuable and broadly informative, it inevitably omits certain high-quality works that do not fall neatly into the selected categories. To complement this overview, I recommend the authors consider adding a supplementary table that systematically summarizes the performance metrics (e.g., switching energy, speed, contrast ratio) across a wider range of reported devices. This would help readers more thoroughly assess the relative advantages of the proposed device. For reference, the authors may consider the comparison approach adopted in [Laser Photonics Rev., 10, 409 (2016)], which not only evaluates device-level metrics but also introduces an additional criterion related to how closely the experimental results reflect all-optical signal processing capabilities.

Author's A8:

We sincerely thank the reviewer for this thoughtful suggestion. We agree that a more comprehensive comparison would provide additional value to the manuscript, especially considering that certain high-quality works may not neatly fit into the categories we initially selected. To address this, we include a supplementary table (Table **S1**) that systematically summarizes the performance metrics of a broader range of reported chip-scale all-optical switches. This table will include key parameters such as switching energy, switching speed, switching contrast, and other relevant metrics across different material platforms and nonlinear mechanisms. In addition to these basic performance metrics, we will also introduce an additional criterion, as suggested by the reviewer, which evaluates how closely the experimental results reflect all-optical signal processing capabilities. This criterion will help readers assess the practical application potential of the devices in comparison to their pure device-level performance. The supplementary table has been added to Section **S12** of the revised Supplementary Information.

(Line 599 in revised Supplementary Information)

S12. Performance Comparison of On-chip All-optical Switches

A variety of chip-scale all-optical switches has been demonstrated across different material platforms and nonlinear mechanisms. Here, we provide a detailed comparison of four key performance metrics: peak power, switching energy, switching time, and switching contrast, as well as criterions that evaluates how closely the experimental results align with all-optical signal processing, i.e., duty cycle and repetition rate, as shown in Table **S1**. The criterions roughly reflect the practical application potential of the devices. The basic demos involve measuring the dynamic characteristics of the switching device using pump-probe techniques (p-p), where the input signal has a very low repetition rate. As the repetition rate increases, the average dissipated power also increases, potentially introducing thermal effects that can impact device performance. Additionally, the incomplete recovery of the signal waveform could affect subsequent bit streams, a phenomenon known as patterning effects. The second level involves using fixed-pattern data streams with high duty cycles and high repetition rate, such as predefined 4-bit repeating data sequences, which are more relevant for practical applications. The demos mostly

close to practical applications build upon the second by applying PRBS data streams for bit-error rate measurements, ensuring the performance of device in practical, real-world scenarios.

Table S1 Comparison of the performance of various on-chip all-optical switches.

Principle	Peak Power	Switching Time	Switching Energy	Switching Contrast	Duty Cycle (%)	Repetition Rate
BFD+TPA/LA [16]	47 μW *	35 ps	0.66 fJ/bit	10 dB	1.4×10^{-2} *	10 MHz $^{p\text{-}p}$
	NA	NA	10.2 fJ/bit	NA	NA	40 Gbps 4-bit pulses
BFD+FCE [17]	10.33 mW *	NA	310 fJ/bit	3 dB	2.4×10^{-1} *	80 MHz $^{p\text{-}p}$
Saturable absorption [18]	167 mW *	260 fs	35 fJ/bit	3.5 dB	2.1×10^{-4} *	10 MHz $^{p\text{-}p}$
$\chi^{(2)}$ nonlinearity [19]	2 W *	46 fs	80 fJ/bit	3 dB	1×10^{-3} *	250 MHz $^{p\text{-}p}$
FCE [20]	5 mW *	30 ps	100 fJ/bit	3 dB	NA	5 Gbps 4-bit pulses
FCE [21]	60 mW *	70 ps	460 fJ/bit	NA	7.7×10^{-3} *	10 MHz $^{p\text{-}p}$
Kerr effect [22]	370 mW *	NA	1124 fJ/bit *	7.7 dB	12.81 *	42.7 Gbps 2^{31}-1 PRBS
Kerr effect [23]	264 mW *	18 ps	720 fJ/bit	NA	2.7×10^{-2} *	100 MHz $^{p\text{-}p}$
ISBT [24]	4.3 W *	1.2 ps	520 fJ/bit	7 dB	9.12×10^{-4} *	76 MHz $^{p\text{-}p}$
**This work**	**7.5 mW**	**9 ps**	**63 fJ/bit**	**8.3 dB**	**33**	**40 Gbps 2^7-1 PRBS (Error-free)**

Notes: NA—not available, BFD—band-filling dispersion, TPA—two-photon absorption, LA—linear absorption, FCE—free-carrier effect, ISBT—inter-sub-band transition, p-p—pump-probe measurement, PRBS—Pseudo-Random Binary Sequence, *—calculated based on available data: The switching energy is defined as the product of the on-chip peak pump power and the effective temporal width of the input pump "1"-bit, while the duty cycle is given by the product of this pulse width and the pulse repetition rate.

…

[16] Nozaki, K. et al. Sub-femtojoule all-optical switching using a photonic-crystal nanocavity. *Nat. Photon*. **4**, 477–483 (2010).

[17] Takiguchi, M. et al. All-Optical InAsP/InP Nanowire Switches Integrated in a Si Photonic Crystal. *ACS Photon*. **7**, 1016-1021 (2020).

[18] Ono, M. et al. Ultrafast and energy-efficient all-optical switching with graphene-loaded deep-subwavelength plasmonic waveguides. *Nat. Photon*. **14**, 37–43 (2020).

[19] Guo, Q. et al. Femtojoule femtosecond all-optical switching in lithium niobate nanophotonics. *Nat. Photon*. **16**, 625–631 (2022).

[20] Moille, G. et al. Integrated all-optical switch with 10 ps time resolution enabled by ALD. *Laser Photonics Rev*. **10**, 409 (2016).

[21] Tanabe,T. et al. Fast all-optical switching using ion-implanted silicon photonic crystal nanocavities. *Appl. Phys. Lett*. **90**, 031115 (2007).

[22] Koos, C. et al. All-optical high-speed signal processing with silicon-organic hybrid slot waveguides. *Nat. Photon*. **3**, 216–219 (2009).

[23] Jason S. et al. Picosecond all-optical switching in hydrogenated amorphous silicon microring resonators. *Opt. Express* **22**, 3797-3810 (2014).

[24] Hu X. et al. Picosecond and low-power all-optical switching based on an organic photonic-bandgap microcavity. *Nat. Photon.* **2**, 185–189 (2008).

Reviewer's Q9:

9. In addition, in Fig. 5, the axes (Peak Power vs. Switching Energy) make direct comparison challenging regarding the key claimed advantage of low peak power at high duty cycle. Duty cycle is plotted separately. Furthermore, the trade-off between speed (switching time) and energy is inherent but not explicitly visualized in a way that highlights their achievement of speed with low energy/peak power. The authors can

consider adding contour lines of constant average power (Peak Power * Duty Cycle) to Figure 5.

Author's A9:

We sincerely thank the reviewer for the thoughtful suggestion regarding the use of contour lines of constant average power in Fig. **5**. For all-optical switching, practical relevance is determined not by a single metric but by the combined performance across multiple, mutually coupled dimensions, including peak power, duty cycle, switching energy, switching speed, and achievable switching contrast. From an application-oriented perspective, it is this multi-parameter balance, rather than any individual figure of merit, that dictates the usability of a switching platform under realistic data-stream conditions. Therefore, the multidimensional radar-plot presentation is adopted to provide an intuitive visualization of the overall device performance across all key metrics simultaneously, while explicitly acknowledging that several of these metrics are intrinsically interdependent.

(1) The central advance of this work is the demonstration of Kerr-based all-optical switching operating at milliwatt-level peak power under a high duty cycle, while maintaining error-free data transmission—an operating regime that remains technically challenging. Therefore, Fig. **5** is intentionally designed to highlight this capability. In its current form, the multidimensional visualization clearly maps the performance landscape and shows that our device occupies a substantially more advantageous region than state-of-the-art reports, particularly in terms of peak power, duty cycle, and switching contrast.

(2) We further note that Fig. **5** compares five key performance metrics that are not independent. For example, the switching energy is jointly determined by the peak power, switching time, and duty cycle, while the switching contrast generally increases with increasing switching energy. Moreover, many prior works report switching energy at a 3-dB contrast point, whereas we define the 63-fJ/bit value at the saturation threshold (corresponding to ~8.3 dB contrast) to ensure optimal error-free performance. A direct comparison of switching energy versus switching time—or peak power versus duty cycle—would therefore require normalization of the switching contrast across all reports, which is not the intent of this figure and would obscure the broader performance landscape. In contrast, the radar-plot representation provides an application-relevant overview of the overall device performance across all key metrics simultaneously.

(3) We also emphasize that the peak power—rather than the average power (Peak Power × Duty Cycle)—is the primary parameter governing the nonlinear phase shift and the achievable switching performance in Kerr-based devices. The average power is primarily constrained by practical considerations such as device damage thresholds, thermal accumulation, and laser source limitations. Importantly, in many previously reported demonstrations, the average power can be kept low by operating at extremely small duty cycles, despite requiring watt-level peak powers. While this approach satisfies thermal constraints, it does not translate into practical switching performance under realistic data-stream conditions. In contrast, our device is specifically engineered—through both structural design and nonlinear-

material enhancement—to intrinsically reduce the required peak power, thereby enabling high-duty-cycle operation within the allowable average-power budget.

For this reason, incorporating contour lines of constant average power may not further strengthen the purpose of this comparison. Since our work does not target reducing the average-power limit itself, plotting contour lines of constant average power would tend to cluster many prior ultrashort-pulse demonstrations into a similar range—despite their reliance on watt-level peak powers and extremely low duty cycles, which inherently restrict their suitability for practical high-duty-cycle operation. In contrast, the focus of our comparison is to highlight devices capable of operating in realistic data-processing regimes. We therefore believe that the current presentation of Fig. **5** most clearly reflects the practical significance and unique advantages of our device. We sincerely hope the reviewer understands our rationale for maintaining the existing visualization.

In sum, in my view, this paper offers innovative results and would be interesting for readers in optical time-frequency manipulation, telecommunication, and signal processing. I can recommend its publication in nature communications once my concerns were well addressed.

Author's reply:

We sincerely thank the reviewer for the positive assessment and for recognizing the significance and potential impact of our work. We greatly appreciate the constructive feedback and have attempted to address all concerns to further strengthen the manuscript.

Reviewer #2

Reviewer's comment:

This manuscript reports a silicon–organic hybrid (SOH) photonic-crystal (PhC) slot nanobeam cavity implementing two-mode Kerr switching via cross-phase modulation between distinct Fano resonances. The authors claim error-free 40-Gbit/s RZ-OOK operation with 33% duty cycle at 7.5 mW peak power and 63 fJ/bit switching energy, with loaded Q factors ~1.8×10^3 and 2.5×10^3 (bandwidths 108 and 77 GHz), and an electric-field overlap integral of 0.69 between the two modes. They attribute the low energy to ultra-tight confinement (small mode volume) and a high-n2 polymer (MEH-PPV) with negligible nonlinear absorption, and they provide coupled-mode/transfer-matrix modeling and design/fabrication details in the Supplementary Information. Here, my major comments:

Author's reply:

We greatly appreciate the reviewer's constructive comments that help us further improve the manuscript. Below we provide detailed, point-by-point responses to each comment and describe the corresponding revisions implemented in the manuscript as well as the supplementary material.

Reviewer's Q1:

1. The reported switching energy (63 fJ/bit at 7.5 mW) is obtained assuming a 40-Gbit/s, 33% RZ duty cycle and an effective "1"-bit width of ~9 ps. Simple multiplication yields 67.5 fJ (7.5 mW × 9 ps), slightly above 63 fJ; at 33% of a 25-ps period the nominal bit-"on" time is 8.25 ps, which would give 61.9 fJ. Please reconcile the exact on-chip pulse width used in the energy calculation, specify whether the 7.5 mW is measured in-fiber, at the chip facet, or as intracavity peak, and propagate the uncertainty (coupling losses, calibration) into the stated 63 fJ/bit. The same issue appears at 30 Gbaud (7.2 mW, 11 ps → ~79 fJ/bit, reported as 80 fJ/bit).

Author's A1:

We sincerely thank the reviewer for the careful quantitative check and for pointing out the need to clarify the definition and calculation process of the reported switching energy. The key point underlying this issue is that the choice of numerical representation and rounding of the duty cycle (33%). Specifically, the calculation process uses the exact fraction $1/3$ rather than the decimal approximation 0.33, which is not due to any inconsistency in the underlying experimental parameters.

In this work, the switching energy is calculated as the product of the on-chip peak pump power and the effective "1"-bit duration of the input pump pulse, determined by the rate and duty cycle of the RZ-OOK data stream. The peak power values reported in the manuscript (e.g., 7.5 mW at 40 Gbit/s and 7.2 mW at 30 Gbit/s) correspond to the peak pump power in the on-chip input waveguide after accounting for the loss of the single-ended grating coupler (-6 dB), rather than the in-fiber power or the intracavity circulating power.

For the 40-Gbit/s experiment with a 33% duty cycle, the effective bit- "on" duration is given by:

$$T = (1/40\ \text{Gbit/s}) \times (1/3) \approx 8.33\ \text{ps}$$

The corresponding switching energy is therefore:

$$E = 7.5 \text{ mW} \times 8.33 \text{ ps} \approx 62.5 \text{ fJ/bit}$$

which is rounded to 63 fJ/bit in the manuscript.

Similarly, for the 30-Gbit/s case:

$$T = (1/30 \text{ Gbit/s}) \times (1/3) \approx 11.11 \text{ ps}$$

The corresponding switching energy is therefore:

$$E = 7.2 \text{ mW} \times 11.11 \text{ ps} \approx 80 \text{ fJ/bit}$$

which is reported as 80 fJ/bit.

We note that the pulse widths of ~9 ps (40 Gbit/s) and ~11 ps (30 Gbit/s) shown in Fig. **3c** and Fig. **S20b** correspond to the measured output probe pulses, which are influenced by the cavity response, detection bandwidth and the signal-to-noise characteristics of the switched output. As can be directly observed in the figures, the output probe pulses exhibit increased intensity noise and temporal jitter compared to the input pump pulses, resulting in visually thicker waveforms. This may introduce a small additional uncertainty in extracting the output pulse width, but does not affect the switching-energy calculation, which is based on the well-defined input pump pulse duration.

To avoid ambiguity, we have revised the manuscript to explicitly state the definition of the peak pump power used in the energy calculation.

(Line 223 in revised manuscript)

Figure **3b** presents the input (upper panel) and output (bottom panel) spectrum measured by an optical spectrum analyzer (OSA), corresponding to an on-chip input peak power of 7.5 mW for the pump light. …

(Line 554 in revised Supplementary Information)

…Figure **S22a** illustrates the output spectrum measured by an optical spectrum analyzer (OSA), corresponding to an on-chip input peak power of 7.2 mW for the pump light. …

Reviewer's Q2:

2. The Authors state the switching time is "ultimately limited by the photon lifetime" of the Fano resonances (1.5 ps and 2.1 ps). Yet the demonstrated switching window is ~9 ps at 40 Gbaud, substantially longer than those lifetimes. Please clarify what sets the observed 9-ps window (modulator waveform shaping, cavity loading, filter response, or thermal detuning compensation) and quantify the contributions with measured instrument responses and deconvolution, not only TCMT fits.

Author's A2:

We sincerely thank the reviewer for this insightful comment and for highlighting the importance of clearly distinguishing between the intrinsic device-level speed limit and the experimentally demonstrated switching window.

In the manuscript, the statement that the switching speed is "ultimately limited by the photon lifetime" specifically refers to the fundamental physical limit imposed by the cavity dynamics, which determines the intrinsic upper bound on the achievable switching speed of the device. However, the evaluation of switching speed from the

photon lifetime is much more relaxed than the applicable speed from a systematic point of view, where the final validation of the switching operation must meet the stringent bit-error-rate (BER) requirements. As the switching speed approaches the upper bound speed limit set by the photon life, the BER measurements are expected to degrade rapidly. Our experiment demonstrates that at a switching speed of 40 GBaud—resulting in a switching window of ~9 ps, as measured from the traces in Fig. **3c**—the device can successfully pass the most rigorous BER test with error rate below 10^{-9}, which confirms the capability of system to operate efficiently at this speed. Nevertheless, as shown by our experimental analysis of the impact of increasing data rates from 20 to 40 GBaud (Fig. **4c** in the main text), the switching contrast is observed to progressively decrease, while the power penalty increases correspondingly. This suggests that further increase of the data rate may degrade BER performance below the stringent 10^{-9} requirement and whether this is acceptable depends on concrete applications. In case of the system with forward-error coding (FEC) techniques, the required pre-FEC BER can be substantially more relaxed (e.g., on the order of $\sim 10^{-3}$) while still achieving very low post-FEC error rates after decoding [*IEEE Photonics Conference (IPC)*, 1-5 (2018)], which could allow further increase in the data rates applied with our device.

Regarding the contributions from the used optical and electronic instruments as well as cavity responses, the red trace in Fig. **3c** in the main text (40 GBaud) corresponds to the input pump pulse train measured in a back-to-back configuration, i.e., without the device under test, and already includes the temporal broadening imposed by the electrical modulation chain, optical modulators, RF drivers, and detection bandwidth. Therefore, the observed switching window reflects the overall system-level temporal response, which shows no significant degradation when compared to the waveform measured at a lower data rate, i.e., Fig. **S20b** in the supplementary material (30 GBaud). Further increase of the data rate would drive our equipment into its speed extremes, which may degrade the generated waveforms from origin; as already discussed, test at higher data rate would lead to further degraded BER performances due to cavity bandwidth limitations, the usability of which depends on concrete system requirements.

To avoid ambiguity, we have revised the manuscript to explicitly distinguish between the intrinsic photon-lifetime-limited switching speed of the device and the experimentally demonstrated system-level switching window. We believe this clarification resolves the discrepancy and more accurately reflects both the fundamental physical limits of the device and the practical constraints of the experimental implementation.

(Line 265 in revised manuscript)

…Figure **4c** further characterizes the switching contrast and power penalty at the saturation threshold under varying signal rates. As the signal rate decreases from 40 GBaud to 20 GBaud, the switching contrast monotonically improves by 3.5 dB (8.3 dB to 11.8 dB), while the power penalty is concomitantly reduced by 3.6 dB. This improvement arises from the relaxation of constraints imposed by the finite optical bandwidth of the nanocavity, consistent with the fundamental trade-off between bandwidth and operation speed. Notably, the evaluation of switching speed

based on the photon lifetime provides a much more relaxed limit compared to the actual achievable switching speed in practical systems, where the final validation of the switching operation must meet the stringent BER requirements. Whether this performance is acceptable for higher data rates depends on the specific requirements of the application, such as systems with forward-error coding (FEC) techniques. As already demonstrated, increasing the data rate results in a progressive degradation of BER performance, underscoring the importance of balancing speed and error tolerance. Nevertheless, error-free operation has been successfully demonstrated at 40 GBaud based on our design, emphasizing the viability of the proposed design for high-speed signal processing.

Reviewer's Q3:

3. The narrative emphasizes negligible TPA/FCA due to polymer nonlinearity, but a PhC slot on SOI will still localize a finite fraction of energy in silicon rails. Provide a quantitative breakdown of modal energy in polymer vs silicon at both resonances, estimate TPA-induced carrier densities at the stated peak powers, and bound FCA-related loss/phase shift during a bit slot. If reverse-biased junctions are unnecessary, justify with measurements (e.g., pump-probe recovery traces vs peak power showing no carrier tail).

Author's A3:

We sincerely thank the reviewer for this important comment regarding the possible contribution of TPA- and FCA-related effects in the silicon rails of the PhC slot cavity. While the nonlinear response in our device is dominated by the polymer-filled slot, a finite fraction of the optical field necessarily resides in the surrounding silicon, and its potential impact therefore merits quantitative assessment.

To address this point, we perform a detailed modal energy analysis for both resonances. According to the revised supplementary information, the fractional energy stored within the polymer-filled slot (≈ 70%) and silicon rails (≈ 30%) is obtained by integrating the transverse modal distribution shown in Fig. **1a** of the main text. Importantly, although a nonzero fraction of energy is localized in silicon, the effective modal area in the silicon region is substantially larger than that in the narrow slot. Since the nonlinear interaction strength scales inversely with the effective mode area, this larger spatial extent significantly reduces the optical intensity—and hence the strength of TPA and carrier generation—in the silicon rails. As a result, the optical intensity in the polymer-filled slot, evaluated at the central cross-section of the nanocavity, is approximately eight times higher than that in the surrounding silicon rails.

Based on these extracted modal distributions, we quantitatively estimate the contributions of two-photon absorption (TPA), free-carrier absorption (FCA), and free-carrier dispersion (FCD) associated with the silicon rails under the experimental operating conditions. A detailed analysis is provided in Section **S3** of the revised Supplementary Information. The results show that, within the on-chip pump power range employed in the experiments (corresponding to an average on-chip power below 3 dBm), the free-carrier–induced loss and phase shift in silicon remain negligibly small and do not produce any measurable impact on the switching dynamics. Consequently,

carrier-related effects in silicon can be safely neglected under the operating conditions of this work.

In addition to this quantitative analysis, our experimental observations provide further support for this conclusion. Figure **3c** shows the switched probe PRBS waveform at 63 fJ/bit under high-duty-cycle operation. While weak pattern-dependent fluctuations can be discerned at the waveform level, no pronounced carrier-induced tail or slow recovery behavior is observed following the pump "1" bits. More importantly, as demonstrated by the open eye diagrams and stable BER performance in Fig. **4**, these minor effects do not compromise system-level operation, and error-free transmission is consistently achieved under all reported operating conditions. While pump-probe recovery measurements are commonly employed to extract carrier lifetimes, the high-duty-cycle data-stream operation used here imposes a more stringent constraint on carrier accumulation and recovery. The absence of observable carrier signatures in both the time-domain waveforms and system-level metrics is fully consistent with the quantitative estimates, confirming that reverse-biased junctions are not required for the present device.

(Line 185 in revised Supplementary Information)

S3. The Impact of Silicon-Related Nonlinear Absorption in Nanocavity

Although the nonlinear response of the present device is dominated by the polymer-filled slot, the optical mode inevitably exhibits a finite overlap with the surrounding silicon rails of the PhC slot nanobeam cavity. As a result, silicon-related nonlinear processes, including two-photon absorption (TPA), free-carrier absorption (FCA), and free-carrier dispersion (FCD), may in principle contribute to the overall switching dynamics and therefore warrant careful evaluation. To examine the contribution of silicon-related nonlinearities, we analyze the modal energy distribution of both Fano resonances (as shown in the inset of Fig. **1a** in the main text) by integrating the simulated electric-field energy density over the polymer-filled slot and the silicon regions, respectively. This analysis shows that approximately 70% of the optical energy is confined within the polymer-filled slot, while the remaining ~30% resides in the silicon rails. Despite this non-negligible fraction of modal energy in silicon, the effective mode area associated with the silicon rails is substantially larger than that of the narrow slot. As a result, the optical intensity in the polymer-filled slot, evaluated at the central cross-section of the nanocavity, is approximately eight times higher than that in the surrounding silicon rails. Since TPA, FCA, and FCD scale with optical intensity rather than stored energy alone, the reduced intensity strongly suppresses silicon-related nonlinear effects under the operating conditions considered here.

To rigorously account for these residual effects, we extend the TCMT framework introduced in Section **S2** to explicitly include contributions from TPA, FCA, and FCD. First, we establish the relationship between the input pump power and the intracavity field, from which the corresponding nonlinear absorption and carrier-induced effects are derived. Specifically, the governing equations presented below are derived by extending Eq. (S8) to include additional contributions from TPA-, FCA-, and FCD-related processes. Because the pump power is substantially

higher than that of the probe, only the pump-induced carrier generation and associated nonlinear effects are considered in this analysis, while probe-induced carrier effects are neglected:

$$\frac{da^p}{dt} = \left(i\Delta\omega - \frac{\gamma_0}{2} - \frac{\gamma_c}{2} - i\delta_{FC} - \frac{\gamma_{TPA}}{2} - \frac{\gamma_{FCA}}{2}\right)a^p + \kappa S^p_{a1+}e^{-i\delta} + \kappa S^p_{a2+}e^{-i\delta} \quad (S24)$$

Here, δ_{FC} denotes the frequency shift induced by FCD, while γ_{TPA} and γ_{FCA} represent the additional cavity decay rates arising from TPA and FCA, respectively. These decay rates are related to the corresponding optical absorption coefficients α_{TPA} and α_{FCA} through $\gamma_{TPA,FCA} = (c/n_{\mathrm{eff}}^{\mathrm{cav.}}) \cdot \alpha_{TPA,FCA}$ (c is the speed of light in vacuum and $n_{\mathrm{eff}}^{cav.}$ is the effective refractive index in the nanocavity). In the nanocavity, the TPA-induced absorption depends on the intracavity pump intensity. Accordingly, the TPA-related absorption coefficient can be written as:

$$\alpha_{TPA} = \frac{\beta_{TPA} c p_{Si}}{n_{Si} V_{TPA}} |a^p|^2 \quad (S25)$$

Where β_{TPA} is the two-photon absorption coefficient of silicon ($\beta_{TPA} \approx 8 \times 10^{-12}\ \mathrm{m \cdot W^{-1}}$), n_{Si} is the refractive index of silicon, V_{TPA} is the effective mode volume associated with the TPA process, and p_{Si} denotes the fraction of the total intracavity optical energy residing in the silicon rails of the nanocavity ($p_{Si} \approx 0.3$).

The FCA-induced absorption depends on the carrier density inside the cavity. The free-carrier density $N(t)$ generated by TPA satisfies the carrier rate equation:

$$\frac{dN(t)}{dt} = \frac{\beta_{TPA} c^2 {p_{Si}}^2}{2\hbar\omega_0^p {n_{Si}}^2 {V_{FCA}}^2} |a^p|^4 - \frac{N(t)}{\tau_{recom}} \quad (S26)$$

where $\hbar$ is the reduced Planck constant ($\hbar = h/2\pi$), V_{FCA} is the effective mode volume associated with FCA, and τ_{recom} is the effective free-carrier lifetime in silicon. For a material containing free carriers, the optical response can be described by the Drude model. The complex permittivity ε' of silicon in the presence of free carriers is given by:

$$\varepsilon' = \varepsilon_1 + j\varepsilon_2 = \varepsilon_0 - \frac{\omega_{plasma}^2}{(\omega_0^p)^2} + j\frac{\omega_{plasma}^2}{(\omega_0^p)^3 \tau_{relax}} \quad (S27)$$

where ε_0 is the intrinsic permittivity of silicon, τ_{relax} is the carrier relaxation time (approximately 0.17 ps for electrons and 0.1 ps for holes), and ω_{plasma} is the plasma frequency. The plasma frequency is related to the free-carrier density $N(t)$ by:

$$\omega_{plasma}^2 = \frac{e^2 N(t)}{\varepsilon_0 m^*} \quad (S28)$$

where e is the elementary charge, and m^* is the effective carrier mass. As a result, the FCA-induced absorption coefficient can be expressed as:

$$\alpha_{FCA} = \frac{\omega_0^p \varepsilon_2}{c n_{Si}} \quad (S29)$$

where ε_2 denotes the imaginary part of the complex permittivity ε'.

By solving Eq. (S24) to (S29) under resonant conditions, we obtain the

relationship between the intracavity power and the input pump power, explicitly accounting for nonlinear absorption effects, as shown in Fig. **S4**. The red dashed line represents the intracavity power calculated without nonlinear absorption, whereas the blue solid curve includes the contributions from carrier-related losses. The yellow curve indicates the corresponding nonlinear loss. As the intracavity power increases, nonlinear losses rise progressively, leading to a reduction in the cavity power enhancement factor and ultimately slowing the growth rate of intracavity power. However, within the on-chip pump power range employed in the experiments—corresponding to an average on-chip power below 3 dBm, as indicated by the gray dashed line in Fig. **S4**—the free-carrier-induced loss and phase shift in silicon remain negligibly small and do not produce any measurable impact on the switching dynamics. Consequently, carrier-related effects in silicon can be safely neglected under the operating conditions of this work.

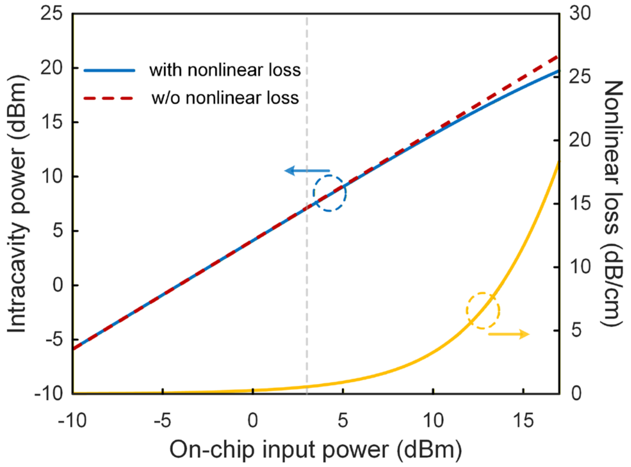

Fig. S4. The impact of silicon-related nonlinear absorption in nanocavity.

Reviewer's Q4:

4. The authors attribute the observed bistable transmission exclusively to the Kerr effect of the polymer, yet no quantitative evidence is provided that rules out thermal contributions. Given the relatively high average powers (milliwatt-level peak with 33% duty cycle) and the limited heat dissipation of the SOH nanobeam cavity, thermal refractive index changes could induce thermal bistability or hysteresis indistinguishable from Kerr bistability in static or slow scans. Please include time-resolved transmission traces at different modulation frequencies to demonstrate that the response follows the instantaneous pump envelope (picosecond-scale) rather than a microsecond-scale thermal process. Additionally, estimate the local temperature rise and compare it to the dn/dT of MEH-PPV and silicon to quantitatively exclude thermal effects. Without such evidence, it remains unclear whether the reported bistability is purely optical or partially thermally driven.

Author's A4:

We sincerely thank the reviewer for this insightful and technically important comment. Distinguishing ultrafast Kerr-induced nonlinear dynamics from thermally driven effects

is particularly critical in hybrid silicon-organic nanocavity platforms. To address this concern, we provide time-resolved measurements under different modulation conditions, together with a quantitative estimation of the possible thermal contribution, which allow us to unambiguously identify the dominant mechanism in the operating regime of our experiments.

1. Time-resolved response under different modulation rates

To directly distinguish ultrafast Kerr-induced switching from slow thermal processes, we perform time-resolved transmission measurements using data streams with different modulation rates, including the 40-GBaud PRBS signal used in the main experiments as well as lower rates of 20 GBaud and 30 GBaud for comparison, as shown in Fig. **R8**. For all measurements, the average on-chip pump power is maintained at approximately 0.8 dBm, ensuring an identical steady-state thermal condition across different modulation rates. Under these conditions, the temporal switching dynamics of the probe signal remain essentially unchanged as the modulation rate varies. Across the investigated data rates, the switched probe waveform consistently follows the pump envelope, exhibiting well-defined and symmetric rising and falling edges with no pattern-dependent distortion or hysteresis-related asymmetry of sufficient magnitude to measurably affect the bit-error-rate performance. Considering that the thermal response time of SOH nanobeam cavities is on the order of microseconds—more than five orders of magnitude longer than the bit period of 25 ps at 40 GBaud—these observations demonstrate that the measured switching dynamics are governed by the instantaneous optical intensity, rather than by thermally induced refractive index changes.

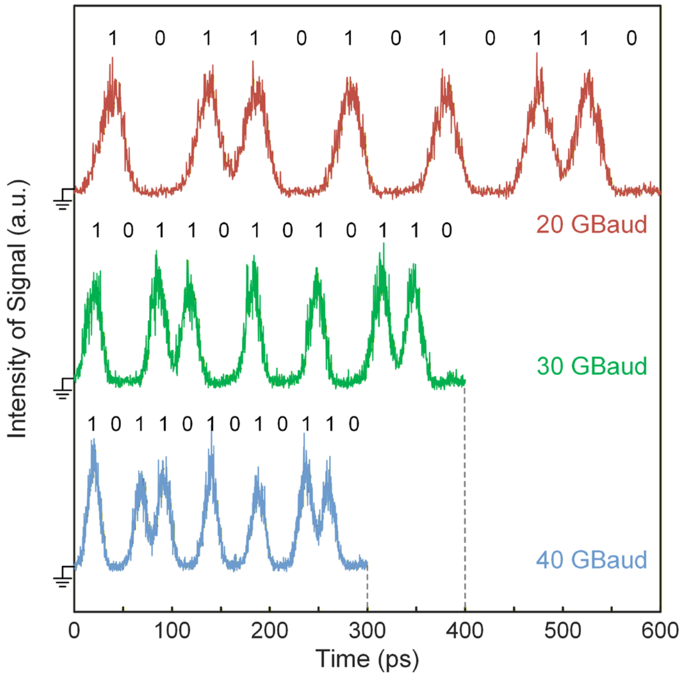

Fig. R8. Output probe pulse trains at different signal rates under identical average on-chip pump power.

2. Quantitative estimation of thermal contribution

In hybrid silicon-organic nanocavity devices, optical absorption can in principle give rise to local heating, which may induce refractive index changes through the thermo-optic effect. To quantitatively assess whether such thermal effects could contribute to the observed nonlinear transmission and switching behavior, we estimate the local temperature rise induced by optical absorption and evaluate the resulting thermo-optic refractive-index change using the dn/dT of both MEH-PPV and silicon to quantitatively exclude thermal effects. A detailed analysis is provided in Section **S8** of the revised Supplementary Information (provided in the box below).

Under continuous-wave excitation, the resonance wavelength exhibits a power-dependent shift resulting from the combined contributions of the Kerr nonlinearity and thermal effects. By quantitatively accounting for the Kerr-induced refractive index change using the independently calibrated nonlinear coefficient, we separate the residual thermal contribution and obtain an effective thermal tuning efficiency of approximately -0.434 nm/mW for the hybrid nanocavity. Notably, the negative sign of this thermal tuning coefficient is consistent with the reported negative thermo-optic coefficient of MEH-PPV and opposite to the positive thermo-optic coefficient of silicon, which would instead produce a red shift under heating. This observation indicates that the effective thermo-optic response of the hybrid nanocavity is dominated by the polymer-filled slot region rather than by the silicon region.

At the experimentally used operating point in the manuscript (average on-chip pump power 1.26 mW for 63 fJ/bit at 40 GBaud and 33% duty cycle), the corresponding steady-state temperature rise is estimated to be approximately 5.9 ℃, using reported thermo-optic coefficients of $dn/dT \approx -1 \times 10^{-4}\ \mathrm{K}^{-1}$ for MEH-PPV [*Polymer (Guildf)*. **47**, 4893–6 (2006)]. At the low-power operating conditions employed in this work, the estimated temperature rise is modest and will not induce material degradation or device instability (a detail as described by **Author's A5**). Importantly, under high-speed data-stream excitation, this thermally induced temperature rise is determined solely by the average pump power and therefore remains constant during bit-level modulation. As a result, thermal effects can at most introduce a static resonance detuning, which is compensated by pre-biasing in the experiment, and cannot account for the observed picosecond-scale, bit-resolved switching dynamics.

(Line 449 in revised Supplementary Information)

…Figure **S16a** shows the transmission spectrum of the new samples without pumping, featuring three distinct resonances corresponding to Fano mode 1, 2 and 3. Under continuous-wave excitation, the resonance wavelength exhibits a clear power-dependent shift arising from the combined action of the Kerr nonlinearity and thermally induced refractive index changes. As shown in Fig. **S16b**, when the pump wavelength is tuned to Fano mode 3, a clear blueshift of mode 1 is observed with increasing input pump power. To quantitatively separate the Kerr and thermal contributions, we first calculate the Kerr-induced refractive index change using the temporal coupled-mode theory (TCMT) model described in Section **S2**, together with the independently calibrated nonlinear coefficient of the polymer. Subsequently, the remaining contribution, obtained by subtracting the Kerr-induced shift from the experimentally measured total shift, can therefore be attributed to thermal effects, as

summarized by the red scatter points in Fig. **S17**. From this analysis, we extract an effective thermal tuning efficiency of approximately $d\lambda/dP \approx -0.434$ nm/mW. Using the experimentally extracted thermal tuning efficiency, we estimate the steady-state temperature rise at the operating point used in the main switching experiments. At the saturation threshold reported in the main text (switching energy of 63 fJ/bit), the corresponding average on-chip pump power is approximately 1.26 mW. This yields an estimated steady-state temperature increase of approximately $\Delta T \approx 5.9$ °C, using the reported thermo-optic coefficient of MEH-PPV ($dn/dT \approx -1 \times 10^{-4}$ K^{-1}) [12,13]. At the low-power operating conditions employed in this work, the estimated temperature rise is modest and will not induce material degradation or device instability.

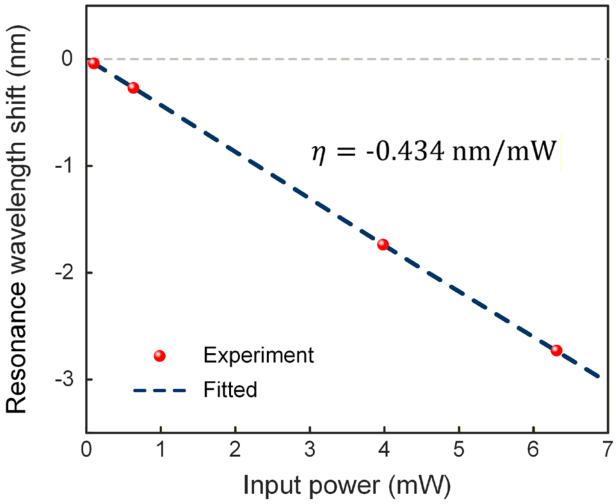

Fig. S17. Resonance wavelength shifts caused by the thermal effect at different input powers.

…

Notably, the extracted thermal tuning efficiency exhibits a negative sign, which is consistent with the reported negative thermo-optic coefficient of the polymer MEH-PPV ($dn/dT < 0$), and opposite to the positive thermo-optic coefficient of silicon ($dn/dT \approx 1.85 \times 10^{-4}$ K^{-1}). This observation indicates that the thermally induced resonance shift is dominated by the polymer-filled slot region, rather than by the surrounding silicon rails. Therefore, this thermally induced refractive index reduction partially offsets the Kerr-induced redshift, thereby degrading the overall signal quality. …Under high-speed PRBS excitation, where the bit period (25 ps at 40 GBaud) is several orders of magnitude shorter than the thermal response time of the nanocavity, this temperature rise is governed solely by the average optical power of the pump ($\Delta n_{thermal} \propto P_{AVG}$) and remains quasi-static during bit-level modulation. To mitigate this effect, a pre-shift of the input wavelength can be applied, in which the input pump wavelength is slowly scanned from the red-detuned side toward the blue side until the cavity reaches a thermally stabilized operating point [15]. Once thermal equilibrium is established at the target average power, the high-speed modulation is applied, effectively compensating the negative thermo-

optic drift and stabilizing the cavity response during ultrafast signal modulation.

…

[13] Zhang, Z. et al. Thermo-optic coefficients of polymers for optical waveguide applications. *Polymer (Guildf)*. **47**, 4893–6 (2006).

[15] Zhang, H. et al. Soliton Microcombs Multiplexing Using Intracavity-Stimulated Brillouin Lasers. *Phys. Rev. Lett*. **130**, 153802 (2023).

Taken together, the time-resolved measurements and the quantitative thermal analysis consistently indicate that the observed bistable transmission and high-speed switching behavior are dominated by the instantaneous Kerr nonlinearity of the polymer, while thermal effects contribute a secondary, quasi-static role under the experimental operating conditions.

Reviewer's Q5:

5. Operation requires pre-shifting the pump/probe wavelengths (e.g., 0.15 nm at 40 Gbaud; 0.13 nm at 30 Gbaud) to compensate the polymer's negative thermo-optic shift. Please provide quantitative thermal time constants, steady-state temperature rise vs average power, and eye/BER stability over ≥ 10-60 minutes at the saturation threshold. Add drift traces of resonance wavelength under PRBS drive, and demonstrate resilience to environmental fluctuations. The Supplement discloses cumulative thermal effects on microsecond scales; show that pattern-dependent baseline wandering is negligible at line-rate for realistic traffic statistics. Also, address polymer photostability and possible irreversible index changes over repeated high-duty-cycle stressing (lifetime test).

Author's A5:

We sincerely thank the reviewer for raising this important point regarding the role of thermo-optic effects and the practical implications of wavelength pre-shifting under high-duty-cycle operation. In hybrid silicon-organic nanocavity devices, thermal effects must be carefully quantified to ensure stable and reproducible operation under sustained excitation.

1. Thermal time constants

To quantitatively address the thermal response time of the device, we first performed a transient thermal simulation using COMSOL Multiphysics based on the full three-dimensional device geometry. The simulation results are detailed in Fig. **S20** of Section **S8** in the revised supplementary information. From this result, the 10%–90% rise time of the temperature response is extracted to be approximately 7.6 μs, indicating a microsecond-scale thermal time constant.

(Line 504 in revised Supplementary Information)

…Transient thermal simulations performed using COMSOL Multiphysics reveal that the thermal response time of the nanocavity is approximately 7.6 μs (see Fig. **S20**).…

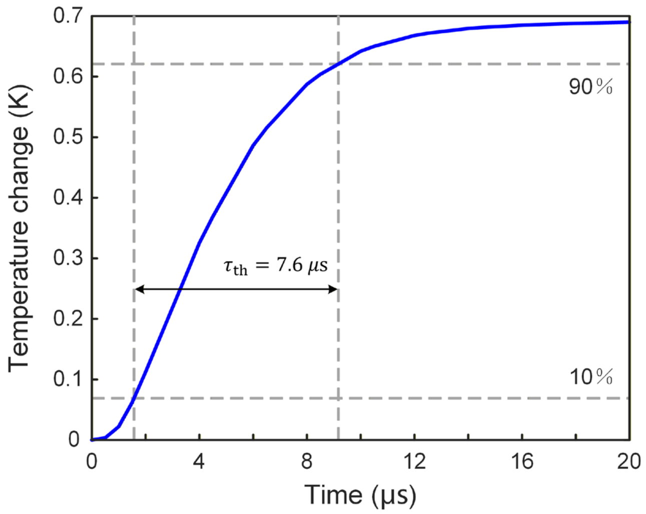

Fig. S20. Temperature transient response at the device center.

To further corroborate this result from an analytical perspective, we consider a lumped thermal model of the nanocavity, in which the thermal time constant is given by

$$\frac{d\Delta T}{dt}=\frac{P_{abs}}{C_{th}}-\frac{\Delta T}{\tau_{th}}$$

Where P_{abs} represents the absorbed light energy, C_{th} denotes the effective thermal capacitance of the cavity region, $C_{th}=\rho_{poly}c_{poly}V$ (ρ_{poly} is the polymer density, c_{poly} is the specific heat capacity of the polymer at atmospheric pressure, and V is the cavity volume), and τ_{th} denotes the temperature decay lifetime, which is determined by the effective thermal resistance R_{th} of the cavity region, $\tau_{th}=C_{th}R_{th}=C_{th}\Delta T/P_{abs}$.

Since detailed values of the mass density and specific heat capacity of MEH-PPV are not explicitly reported in the literature, we approximate these parameters using typical values of structurally similar aromatic polymer derivatives, such as polystyrene (PS) and PMMA. This approximation is reasonable because the thermal properties of polymers are primarily governed by vibrational degrees of freedom of the polymer backbone rather than by electronic conjugation. Accordingly, the effective thermal capacitance on the order of $3.5\times10^{-13}\ \mathrm{J\cdot K^{-1}}$ is adopted in the model. We note that this choice affects only the absolute value of the thermal time constant, while does not alter the qualitative conclusion regarding the separation of thermal and optical timescales. Using the experimentally calibrated thermal tuning efficiency of approximately −0.434 nm/mW, we further estimate that only a small fraction of the input optical power is converted into heat, corresponding to an absorption-to-heat conversion efficiency on the order of ~0.1%. Incorporating this value into the thermal model yields a thermal response time on the order of several microseconds, in good agreement with the COMSOL simulation.

Taken together, both numerical simulation and analytical estimation consistently indicate that the thermal response of the hybrid silicon-organic nanocavity occurs on a microsecond timescale. This is more than five orders of magnitude longer than the bit

period at 40 GBaud (i.e., 25 ps), confirming that thermal processes cannot follow the high-speed modulation and therefore do not limit or govern the observed picosecond-scale switching dynamics.

2. Steady-state temperature rise vs average power

Under continuous-wave excitation, we experimentally observe a linear resonance wavelength shift with increasing pump power. As detailed in our response to **Question 4**, by subtracting the independently calibrated Kerr-induced contribution from the measured wavelength shift, we extract an effective thermal tuning efficiency of approximately −0.434 nm/mW. This experimentally determined relationship directly provides the steady-state thermal response as a function of average power, without relying on additional fitting parameters. At the saturation-threshold operating point used in the switching experiments (63 fJ/bit, corresponding to an average on-chip pump power of approximately 1.26 mW), the resulting steady-state temperature rise is estimated to be modest (≈ 5.9 ℃), well below levels that would induce material degradation or compromise device stability as explained later.

(Line 452 in revised Supplementary Information)

…As shown in Fig. **S16b**, when the pump wavelength is tuned to Fano mode 3, a clear blueshift of mode 1 is observed with increasing input pump power. To quantitatively separate the Kerr and thermal contributions, we first calculate the Kerr-induced refractive index change using the temporal coupled-mode theory (TCMT) model described in Section **S2**, together with the independently calibrated nonlinear coefficient of the polymer. Subsequently, the remaining contribution, obtained by subtracting the Kerr-induced shift from the experimentally measured total shift, can therefore be attributed to thermal effects, as summarized by the red scatter points in Fig. **S17**. From this analysis, we extract an effective thermal tuning efficiency of approximately $d\lambda/dP \approx -0.434\ \mathrm{nm/mW}$….

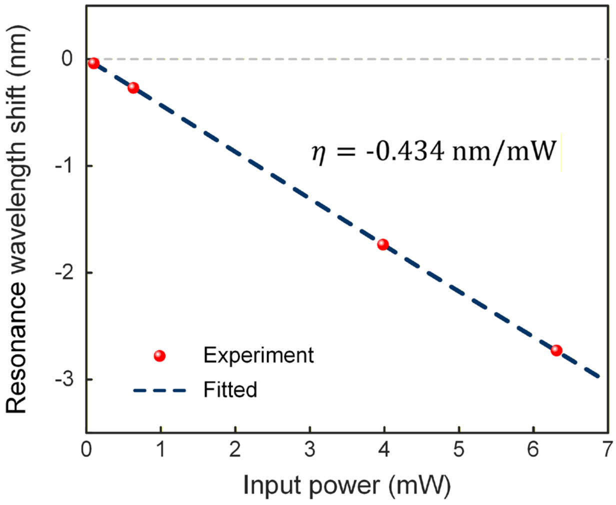

Fig. S17. Resonance wavelength shifts caused by the thermal effect at different input powers.

3. Long-term stability and polymer photostability under sustained high-speed operation

To address concerns regarding long-term operational stability, environmental robustness, pattern-dependent baseline wandering, and potential irreversible refractive-index changes in the polymer under repeated high-duty-cycle stressing, we further investigate the device performance over extended time durations under the reported switching conditions, without employing any feedback control or external temperature stabilization. Specifically, the device is continuously operated for 30 minutes at 40 GBaud, 63 fJ/bit, and 33% duty cycle, while monitoring the key performance metric of bit-error rate (BER). Throughout the entire measurement duration, the BER remains below 10^{-9}, corresponding to error-free operation, with no abrupt degradation or long-term trend indicative of accumulated drift or instability. A detailed analysis is provided in Section **S11** of the revised Supplementary Information.

BER represents the most stringent and system-relevant performance metric, as it inherently integrates the combined effects of resonance wavelength drift, thermal fluctuations, environmental perturbations, and any pattern-dependent baseline wandering. If significant irreversible refractive-index changes in the polymer, cumulative thermal drift, or pattern-dependent thermal accumulation are present under PRBS driving, they would manifest as a measurable BER degradation or sudden error bursts during continuous operation. None of these signatures are observed within the 30-minute measurement window.

(Line 577 in revised Supplementary Information)

S11. Long-term stability measurements

To further evaluate the operational stability under sustained high-speed switching, a long-term stability measurement is performed under fixed operating conditions corresponding to the saturation threshold reported in the main text, without employing any feedback control or external temperature stabilization. Specifically, the device is continuously operated at a switching energy of 63 fJ/bit, a signal rate of 40 GBaud, and a duty cycle of 33% for a duration of 30 minutes. During the measurement, the received optical power at the photodetector (PD) is fixed at -1.8 dBm, and the bit-error rate (BER) is continuously monitored using the experimental setup described in Fig. **3a**. The measured BER remains below 10^{-9} throughout the entire 30-minute interval, confirming sustained error-free operation, as shown in Fig. **S23**. During the first ~20 minutes, the BER is essentially constant, indicating stable switching behavior under steady-state thermal conditions. In the subsequent ~10 minutes, a slight increase in the measured BER is observed, which is attributed primarily to slow fluctuations in the grating-coupler coupling efficiency and minor cavity detuning. Importantly, the BER remains well below the error-free threshold, and no abrupt degradation or instability is observed. These results demonstrate that, under the reported operating conditions, the modest and quasi-static temperature rise does not compromise the long-term stability of the all-optical switching performance. For future implementations targeting extended deployment times or more demanding environments, additional stabilization strategies—such as active feedback control of the pump wavelength or optical power, chip-level

temperature control, and optimized device packaging—can be readily incorporated to further enhance system robustness.

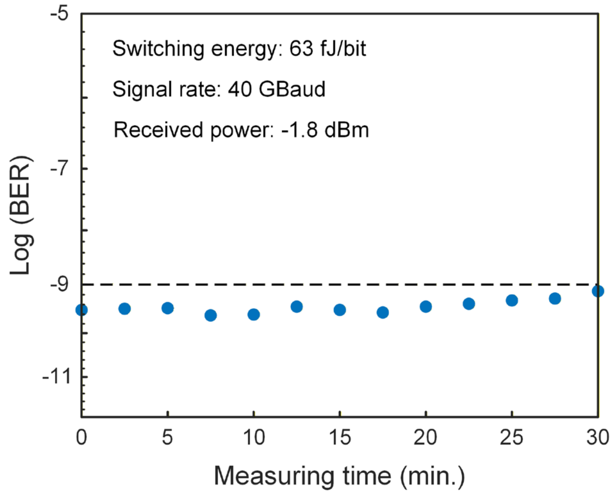

Fig. S23. BER stability measurement over 30 minutes under continuous 40-GBaud switching operation.

Meanwhile, to evaluate the thermal stability of the polymer material (MEH-PPV), we perform complementary thermal analysis using thermogravimetric analysis (TGA) and differential scanning calorimetry (DSC). TGA is conducted in a nitrogen atmosphere to measure the thermal decomposition point of MEH-PPV. The results indicate that the material begins to decompose around 350 °C, which is significantly higher than the operating temperatures used in our experiments, suggesting that thermal degradation under typical experimental conditions is unlikely to occur. DSC measurements are carried out under nitrogen atmosphere to evaluate the melting point and thermal response of the material. The results indicate that no prominent peaks associated with melting are observed during both the first heating and second heating, indicating that the melting point of MEH-PPV is above 320 °C. These results further confirm the thermal stability of MEH-PPV, ensuring that thermal effects under operational conditions do not significantly contribute to performance degradation. A detailed analysis is provided in Section **S8** of the revised Supplementary Information.

(Line 468 in revised Supplementary Information)

…

Additionally, to evaluate the thermal stability of the polymer material (MEH-PPV), we perform complementary thermal analysis using thermogravimetric analysis (TGA) and differential scanning calorimetry (DSC). TGA is conducted in a nitrogen atmosphere to measure the thermal decomposition point of MEH-PPV. As shown in Fig. **S18**, we test different samples: samples 1-3 are MEH-PPV films of varying weight, prepared by dissolving MEH-PPV powder in toluene, depositing it onto substrates, and drying them to obtain films of approximately 26 μm thickness; Sample 4 is the original solid MEH-PPV powder. The TGA results indicate that the material begins to decompose around 350 °C, which is significantly higher than the

operating temperatures used in our experiments, suggesting that thermal degradation under typical experimental conditions is unlikely to occur.

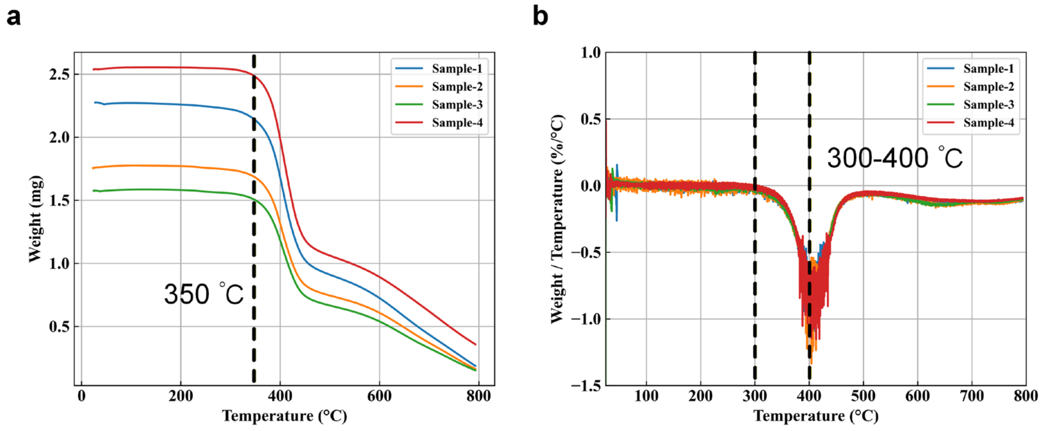

Fig. S18. Thermogravimetric analysis (TGA) of MEH-PPV samples in a nitrogen atmosphere.

DSC measurements are carried out under nitrogen atmosphere to evaluate the melting point and thermal response of the material. As shown in Fig. **S19**(a) and (b), no prominent peaks associated with melting are observed during both the first heating and second heating, indicating that the melting point of MEH-PPV is above 320 °C (positive heat flow corresponds to exothermic (heat-releasing) processes, while negative heat flow corresponds to endothermic (heat-absorbing) processes). Additionally, based on previous TGA data, it is likely that the polymer begins to decompose before it melts. The first peak observed in the DSC measurement corresponds to the thermal balance of the system and is independent of the material itself. These results further confirm the thermal stability of MEH-PPV, ensuring that thermal effects under operational conditions do not significantly contribute to performance degradation. For future scaling toward higher power handling or broader operating scenarios, the thermal robustness of the nonlinear polymer can be further enhanced through materials and process engineering. Building on recent advances in thermally stable and crosslinked organic polymer systems [14], the nonlinear polymer may be spin-coated in combination with an imprint resist or cross-linkable host matrix, followed by ultraviolet curing. Such approaches have been shown to increase the glass transition temperature and suppress thermally induced polymer reflow, thereby providing a viable pathway for improving the thermal tolerance of hybrid silicon-organic nanophotonic devices.

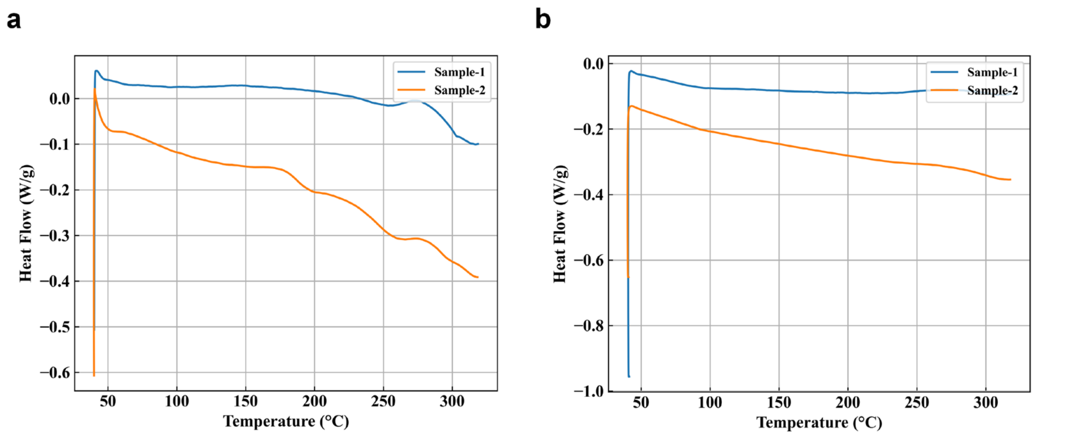

Fig. S19. Differential scanning calorimetry (DSC) of MEH-PPV samples under nitrogen atmosphere during the first heating (a) and second heating (b).

…

[14] Xu, H. et al. Ultrahigh Performance Cross-Linkable Organic Electro-Optic Material for Hybrid Modulators. *Chemistry of Materials* **37**, 12 (2025).

Additionally, we conduct lifetime testing on a 10 mm×10 mm MEH-PPV film (quartz substrate) to further investigate the material photostability under sustained illumination. Firstly, we perform Z-scan measurements with a minimal input power, corresponding to a focal intensity of approximately 29 GW/cm², to determine the nonlinear refractive index (n_2) and nonlinear absorption of the polymer. The Z-scan setup utilize femtosecond pulses at 1550 nm, generated by an optical parametric amplifier (OPA) from a 1030 nm source. Closed-aperture scans are conducted to extract the nonlinear refractive index, while open-aperture scans are performed simultaneously to assess nonlinear absorption. In the closed-aperture configuration, we observe a clear valley-peak feature, confirming the presence of positive Kerr nonlinearity (self-focusing), as shown in Fig. **R10(a), R11(a)** and **R12(a)**. The measured n_2 values at the three points are $3.09 \times 10^{-16}\ \mathrm{m^2/W}$, $1.93 \times 10^{-16}\ \mathrm{m^2/W}$, and $9.22 \times 10^{-17}\ \mathrm{m^2/W}$, respectively. The differences in these values are primarily due to the non-uniform thickness of the film and potentially minor errors introduced by the low test-power. In the open-aperture scan, the normalized power remains flat, indicating negligible nonlinear absorption as shown in Fig. **R13**. Subsequently, we progressively increase the laser power, with focal intensities raised from 29 GW/cm² to 34 GW/cm², 39 GW/cm², 44 GW/cm², and 49 GW/cm², maintaining continuous illumination for 30 minutes followed by 10 minutes of shading treatment. After each irradiation, n_2 measurements are repeated under the initial conditions (29 GW/cm²), as shown in Fig. **R10, R11** and **R12**. The total duration of lifetime testing is 2 hours. The results, presented in Fig. **R9**, clearly demonstrate a noticeable decrease in n_2 as the power increases, with n_2 remaining consistently above $6.81 \times 10^{-17}\ \mathrm{m^2/W}$. Notably, n_2 remained approximately 15 times that of silicon ($4.5 \times 10^{-18}\ \mathrm{m^2/W}$), indicating that the nonlinear refractive index of polymer maintains a high level even after prolonged exposure to high-power illumination.

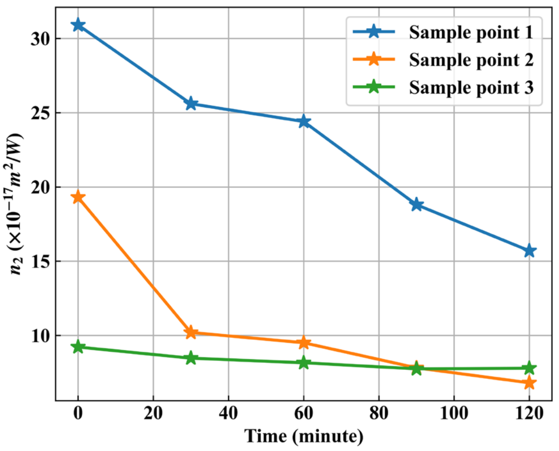

Fig. R9. Statistical analysis of the nonlinear refractive index n_2 of the polymer under different light intensities after 30 minutes of illumination.

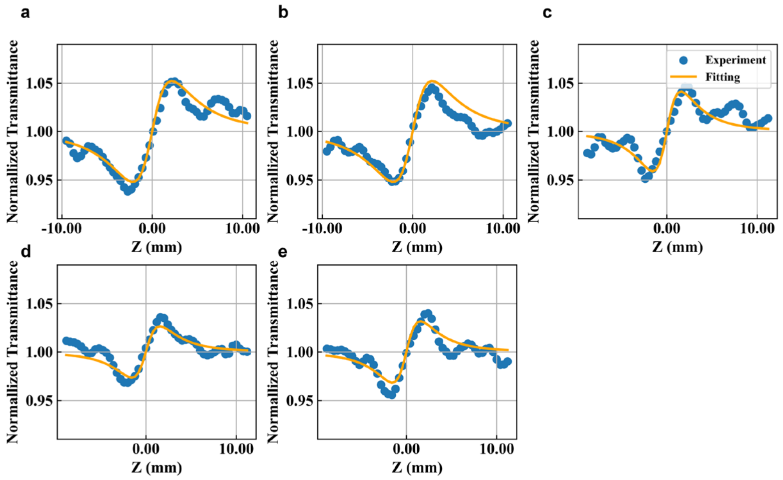

Fig. R10. Z-scan measurement of the nonlinear refractive index n_2 of Sample point 1 in the closed aperture configuration. The initial state measurement is performed under an illumination intensity of 29 GW/cm² **(a)**. Subsequently, the material is illuminated for 30 minutes with intensities of 34 GW/cm² **(b)**, 39 GW/cm² **(c)**, 44 GW/cm² **(d)**, and 49 GW/cm² **(e)**, and the results are tested using the initial conditions (29 GW/cm²).

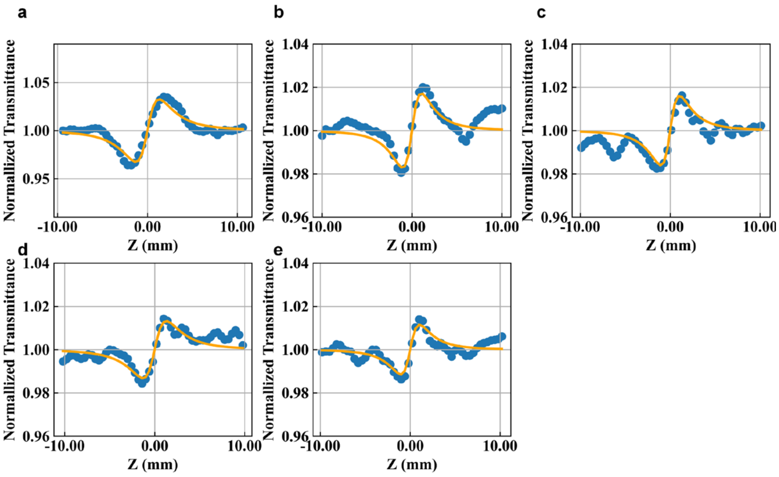

Fig. R11. Z-scan measurement of the nonlinear refractive index n_2 of Sample point 2 in the closed aperture configuration. The initial state measurement is performed under an illumination intensity of 29 GW/cm² **(a)**. Subsequently, the material is illuminated for 30 minutes with intensities of 34 GW/cm² **(b)**, 39 GW/cm² **(c)**, 44 GW/cm² **(d)**, and 49 GW/cm² **(e)**, and the results are tested using the initial conditions (29 GW/cm²).

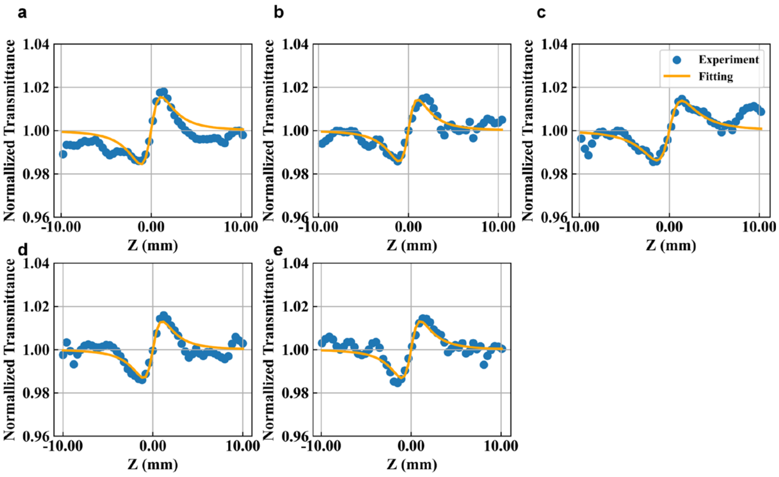

Fig. R12. Z-scan measurement of the nonlinear refractive index n_2 of Sample point 3 in the closed aperture configuration. The initial state measurement is performed under an illumination intensity of 29 GW/cm² **(a)**. Subsequently, the material is illuminated for 30 minutes with intensities of 34 GW/cm² **(b)**, 39 GW/cm² **(c)**, 44 GW/cm² **(d)**, and 49 GW/cm² **(e)**, and the results are tested using the initial conditions (29 GW/cm²).

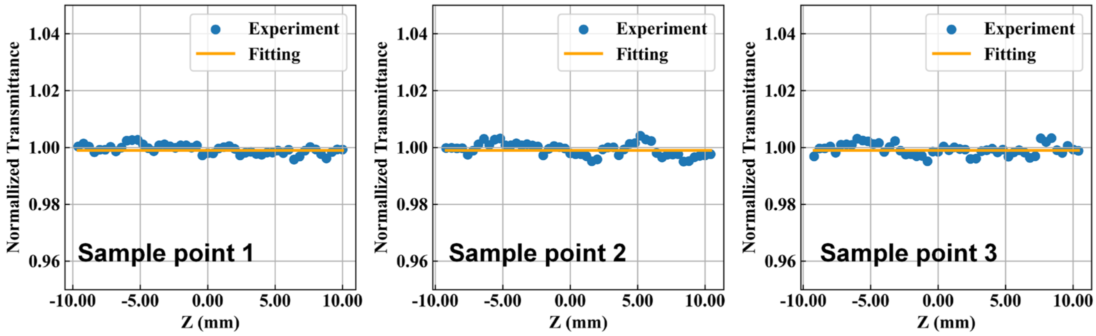

Fig. R13. Open aperture Z-scan measurement of the MEH-PPV film for absorption characterization.

Taken together, these observations indicate that the polymer exhibits stable optical properties without observable irreversible refractive-index changes or photodegradation affecting device performance within the experimentally relevant timeframe and operating conditions reported in this work. Furthermore, both thermal analyses and Z-scan measurements demonstrate that MEH-PPV exhibits excellent thermal and optical stability, making it a suitable material for high-speed, high-duty-cycle all-optical switching applications.

Reviewer's Q6:

6. The overlap integral between the two Fano modes is cited as 0.69 from simulation; provide experimental corroboration (e.g., cross-phase modulation efficiency vs detuning for both mode pairs, or heterodyne measurements of XPM-induced resonance pulls). Include sensitivity of overlap and Q to fabrication tolerances (slot width, hole radius/spacing, bus gap) and report device-to-device statistics (N≥10), not just a single exemplar. The FP-induced Fano shape is central; demonstrate that the 1.4-nm ripple from facet/air-hole reflections does not degrade BER across wavelength drift.

Author's A6:

We sincerely thank the reviewer for these insightful comments, which highlight important aspects related to mode overlap, fabrication robustness, and the influence of parasitic Fabry-Pérot interference. These considerations are indeed central to the interpretation and practical reliability of the reported Fano-resonance-based switching behavior. We address each point in detail below.

1. Experimental corroboration of the mode overlap factor

In the present device, the effective nonlinear interaction strength between the two Fano modes is jointly determined by several parameters, including the field overlap integral ($C_{overlap}$), the loaded Q-factors of the two resonances, the effective mode volume, and the actual nonlinear refractive index of the polymer. As shown in Supplementary Information Eq. (S10) and Eq. (S11) in Section **S2,** the Kerr- and cross-phase-modulation-induced resonance shift is governed by the combined effect of these parameters, rather than by the overlap factor alone.

Based on this framework, we quantitatively relate the experimentally measured switching behavior to the effective nonlinear coupling strength between the two modes. Specifically, by fitting the experimentally measured evolution of switching contrast versus pump energy (Fig. **4a** in the main text) using this TCMT model, we extract an effective mode overlap of approximately 0.42. In the fitting procedure, all other parameters are fixed to independently determined or experimentally measured values, including the loaded Q-factors extracted from the transmission spectra in Fig. **2b** and the nonlinear refractive index of the polymer reported in the literature [*Photon. Res.* **10**, 50–58 (2022)]. Using this approach, the experimentally observed evolution of switching contrast as a function of pump energy can be consistently reproduced with this single free parameter. The experimentally inferred overlap is moderately lower than the simulated value of 0.69. This discrepancy is expected and can be attributed to several practical factors, including non-ideal polymer infiltration into the slot, fabrication-induced variations in slot width and hole geometry, and deviations of the actual modal field distributions from idealized simulation conditions.

Taken together, these results provide an indirect yet quantitative experimental corroboration of the mode overlap, and the experimentally observed switching behavior is compatible with the mode-interaction strength inferred from simulations, within the uncertainties associated with practical device fabrication and material implementation.

2. Fabrication robustness

To evaluate the sensitivity of the cavity performance to fabrication tolerances, we perform full-wave simulations by independently varying key geometric parameters, including the bus-cavity gap, air-hole radius, and slot width, while keeping all other parameters fixed. The following analysis focuses on Fano mode 1, which is directly involved in the switching operation; we note that Fano mode 2 exhibits qualitatively similar trends with respect to geometric variations and therefore is not discussed separately for brevity.

Figure **R14** summarizes the dependence of the loaded Q-factor and resonance wavelength on these parameters. Specifically, as the bus-cavity gap decreases, the coupling strength between the bus waveguide and the cavity increases, leading to a reduction of the loaded Q-factor, while the resonance wavelength remains nearly unchanged. Conversely, increasing the gap weakens the external coupling and results in a higher loaded Q-factor, consistent with standard waveguide-cavity coupling behavior. Increasing the air-hole radius results in a clear blueshift of the resonance wavelength, reflecting the reduction of the effective refractive index of the nanocavity. Over the investigated range, the loaded Q-factor remains largely unchanged and only exhibits a moderate decrease when the hole-radius variation becomes sufficiently large, indicating that the cavity maintains good optical confinement under realistic fabrication deviations. Similarly, enlarging the slot width results in a monotonic blueshift of the resonance wavelength accompanied by a gradual increase in the loaded Q-factor.

Importantly, the effective mode overlap between the two Fano modes remains largely insensitive to variations in both air-hole radius and slot width. Over the full range of simulated fabrication deviations, the overlap integral varies only slightly,

remaining within the range of approximately 0.68–0.70. This robustness indicates that the strong nonlinear interaction between the two modes is preserved even in the presence of realistic geometric fluctuations.

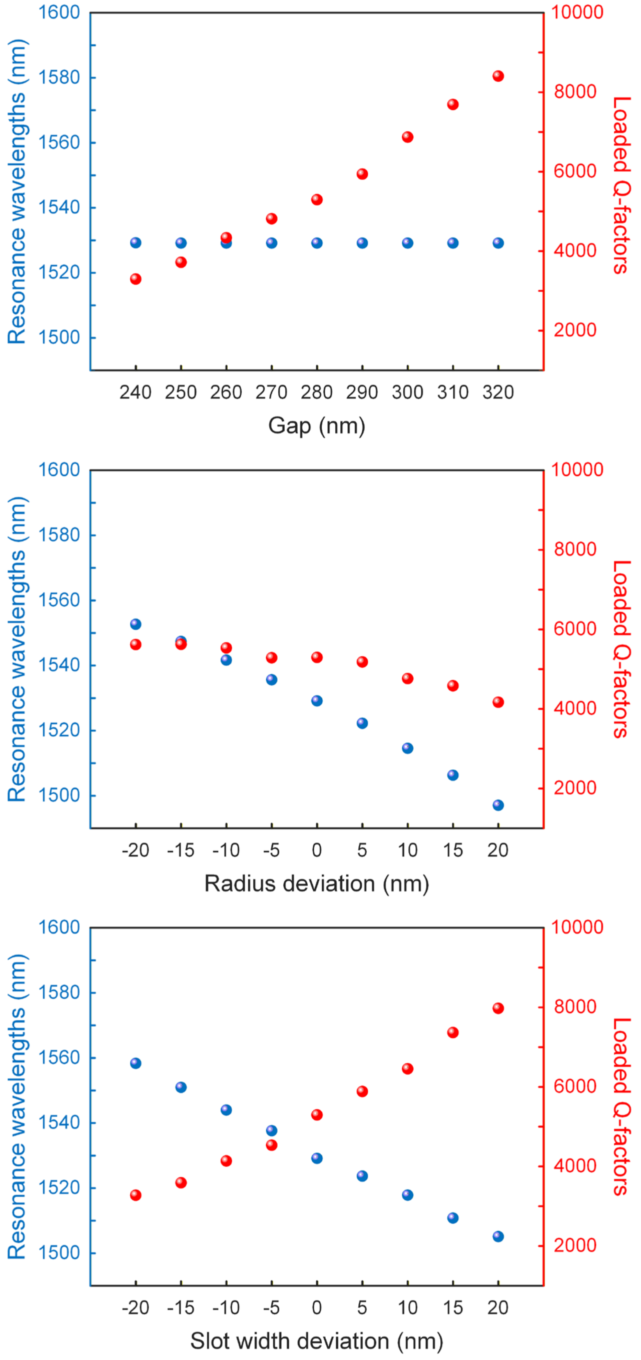

Fig. R14. Simulated impact of geometric variations on the Fano-resonant nanocavity.

In addition to simulation-based analysis, we experimentally assessed device-to-device variability by fabricating twelve nominally identical devices on the same chip using the same design parameters as those reported in the main text. Statistical analysis of the measured transmission spectra shows that the resonance wavelength of Fano mode 1 varies within approximately 1530 nm to 1540 nm, while the loaded Q-factor

spans a range of approximately 1500-2500. A more detailed analysis is provided in Section **S7** of the revised Supplementary Information. These variations are primarily attributed to non-uniformities in polymer spin coating, etching-induced dimensional fluctuations, and local thickness variations of the SOI top silicon layer (210-230 nm), all of which are common in hybrid silicon-organic nanophotonic platforms. Despite these variations, all measured devices consistently exhibit the characteristic Fano line shape and well-defined cavity resonances, indicating that the formation of the Fano response is tolerant to realistic fabrication variations.

(Line 405 in revised Supplementary Information)

…

To evaluate device-to-device variability arising from fabrication tolerances, we fabricate twelve nominally identical SOH nanocavity devices on the same chip using the same design parameters as those reported in the main text. Figure **S14** summarizes the measured resonance wavelength and loaded Q-factor of Fano mode 1 extracted from the transmission spectra of these devices. The resonance wavelength exhibits a spread from approximately 1530 nm to 1540 nm, while the corresponding loaded Q-factor varies between about 1500 and 2500. Such variations are primarily attributed to inevitable fabrication-induced nonuniformities, including fluctuations in polymer spin-coating thickness, etching-induced deviations in slot width and air-hole dimensions, as well as local thickness variations of the SOI top silicon layer (210-230 nm). Despite these variations, all measured devices consistently exhibit the characteristic Fano line shape and well-defined cavity resonances, indicating that the formation of the Fano response is tolerant to realistic fabrication variations. These statistics confirm that the reported device performance is representative rather than anecdotal, and that realistic process variations can be readily accommodated through parameter scanning.

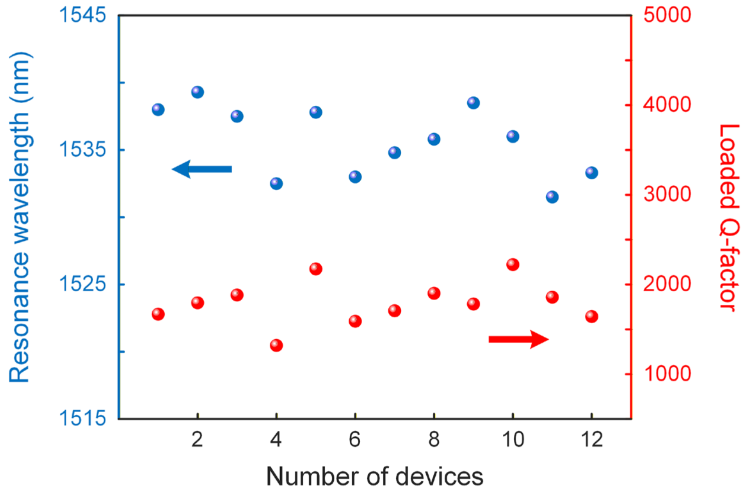

Fig. S14. Statistical distribution of the resonance wavelength (left axis) and loaded Q-factor (right axis) for identical devices fabricated on the same chip.

3. The influence of parasitic Fabry-Pérot interference

In the experimentally measured transmission spectra, small periodic ripples with a characteristic period of approximately 1.4 nm are observed. These ripples originate from weak FP interference caused by residual reflections between the air holes of the bus waveguide and the input/output facets. To account for this effect, we have revised the transmission model by explicitly incorporating the FP-induced periodic modulation, as described in the revised manuscript and Supplementary Information. With this modification, the theoretical spectra show improved agreement with the experimental measurements, capturing both the intrinsic Fano resonance and the superimposed weak FP oscillations.

(Line 491 in revised manuscript)

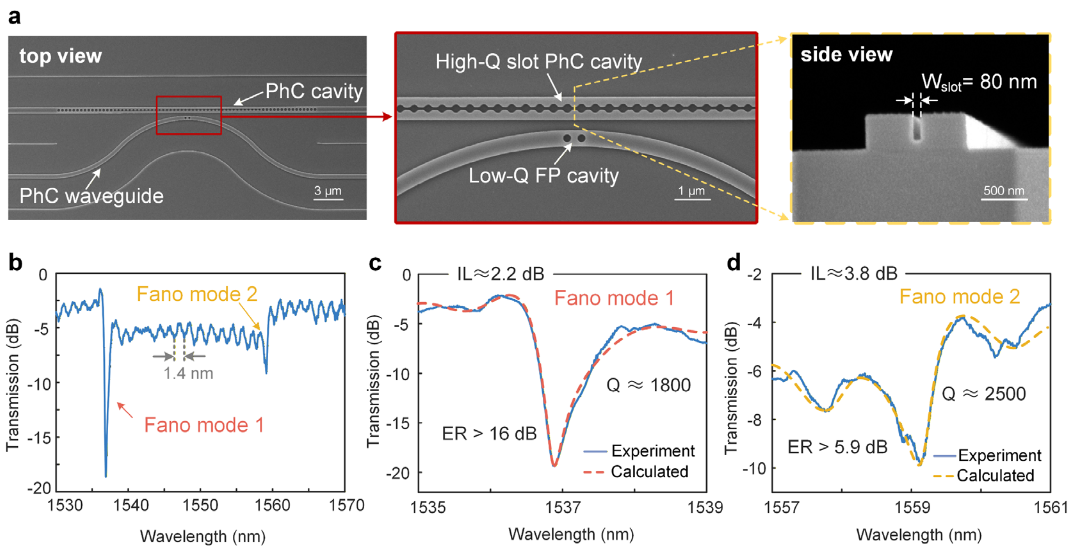

Fig. 2. SEM images and transmission spectra of the fabricated samples. …

(Line 98 in revised Supplementary Information)

…By substituting the Eq. (S2) to (S4) into Eq. (S1), the transmission spectrum of the structure can be expressed as follows:

$$T = \left|\frac{S_{2-}}{S_{1+}}\right|^2 = \left|\frac{(\Delta\omega + i\gamma_0/2)t^2e^{2i\delta}}{(\Delta\omega + i\gamma_0/2 - i\gamma_c/2)r^2e^{4i\delta} + i\gamma_c re^{2i\delta} - \Delta\omega - i\gamma_0/2 - i\gamma_c/2}\right|^2 \quad \text{(S5)}$$

According to the previous study [3], adjusting the cavity length (L_{FP}) of the F-P resonator allows for effective tuning of the Fano line shape. In addition, the experimentally measured transmission spectrum exhibits small periodic ripples with a characteristic period of approximately 1.4 nm originating from residual F-P interference due to weak reflections at the input/output facets and air-hole interfaces. This effect can be incorporated as a slowly varying multiplicative modulation term in the overall transmission function, $T_{exp} = T \times [1 + \alpha_{FP}T_{FP}]$ (where T refers to Eq. (S5), α_{FP} denotes the small modulation depth and T_{FP} represents a normalized periodic interference function). …

Based on the revised model, we further perform dynamic switching simulations that explicitly include the FP-induced spectral ripples. The simulations are carried out

using a 40-GBaud return-to-zero on–off keying (RZ-OOK) pump sequence with a 33% duty cycle, driven by a representative 20-bit PRBS sequence, consistent with the experimental conditions used in the main text. The resulting eye diagrams, shown in Fig. **R15**, indicate that the inclusion of the FP-induced spectral ripple leads to negligible changes in the temporal waveform and eye opening compared to the case without FP perturbations. This behavior arises because the FP ripple introduces a quasi-static spectral modulation on a wavelength scale (~1.4 nm) that is much broader than the effective resonance linewidth governing the switching dynamics. As a result, the FP modulation does not introduce additional dynamic degrees of freedom at the symbol timescale and does not give rise to pattern-dependent temporal distortions under high-speed modulation. Importantly, this conclusion is fully supported by experimental observations. High-speed data-stream measurements, together with 30-minute continuous BER stability measurements at the saturation threshold, show no observable BER degradation, eye closure, or pattern-dependent penalties attributable to the 1.4-nm FP ripple, even in the presence of small wavelength drifts. These results demonstrate that the FP-induced oscillations arising from facet and air-hole reflections have negligible impact on the high-speed switching performance or BER stability of the device under realistic operating conditions.

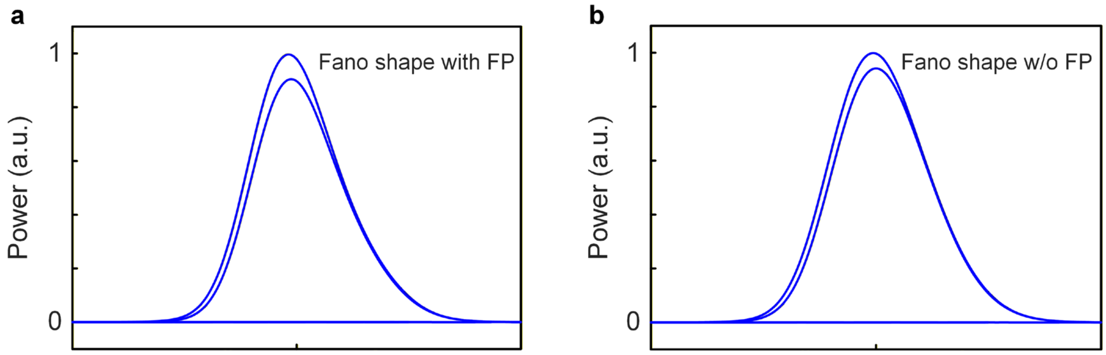

Fig. R15. Simulated RZ-OOK eye diagrams of the switched output signal at 40 GBaud, with (a) and without (b) FP-induced spectral ripple included in the transmission model.

Reviewer's Q7:

7. Figure 5 compares peak power, duty cycle, energy/bit, contrast, and switching time across mechanisms. Ensure apples-to-apples normalization: for Kerr devices, quote on-chip energies with explicit coupling losses; for saturable-absorber/$\chi(2)$ devices, indicate whether femtosecond-pulse, ultra-low duty-cycle operation is being compared to your high-duty-cycle case. Provide a table listing each data point's definitions (duty cycle, PRBS length, pattern dependence criteria, BER floor) and references.

Author's A7:

We sincerely thank the reviewer for this helpful suggestion regarding the normalization and definition of the metrics compared in Fig. **5**. In response, we add a supplementary table (Table **S1**) that systematically summarizes a broader set of reported devices, explicitly specifying key experimental definitions and conditions, including duty cycle, repetition rate, excitation format (e.g., ultrashort pump-probe versus high-speed data

streams), PRBS length where applicable, and the basis used for reporting peak power, switching energy, switching time, and switching contrast. In addition, we introduce an extra criterion that evaluates how closely each experiment reflects realistic all-optical signal processing conditions, thereby helping readers distinguish between device-level demonstrations and system-relevant switching performance.

For Kerr-based devices (including our own), we consistently quote on-chip peak power and on-chip switching energy, explicitly excluding coupling losses. Devices based on saturable absorption, $\chi^{(2)}$ nonlinearities, or other schemes that rely on femtosecond-pulse excitation with extremely low duty cycles are also included in Fig. **5** to provide a unified overview. While ultrashort-pulse excitation can indeed yield very low switching energies, such results do not directly represent operation under continuous data-stream conditions. The supplementary table has been added to Section **S12** of the revised Supplementary Information.

(Line 599 in revised Supplementary Information)

S12. Performance Comparison of On-chip All-optical Switches

A variety of chip-scale all-optical switches has been demonstrated across different material platforms and nonlinear mechanisms. Here, we provide a detailed comparison of four key performance metrics: peak power, switching energy, switching time, and switching contrast, as well as criterions that evaluates how closely the experimental results align with all-optical signal processing, i.e., duty cycle and repetition rate, as shown in Table **S1**. The criterions roughly reflect the practical application potential of the devices. The basic demos involve measuring the dynamic characteristics of the switching device using pump-probe techniques (p-p), where the input signal has a very low repetition rate. As the repetition rate increases, the average dissipated power also increases, potentially introducing thermal effects that can impact device performance. Additionally, the incomplete recovery of the signal waveform could affect subsequent bit streams, a phenomenon known as patterning effects. The second level involves using fixed-pattern data streams with high duty cycles and high repetition rate, such as predefined 4-bit repeating data sequences, which are more relevant for practical applications. The demos mostly close to practical applications build upon the second by applying PRBS data streams for bit-error rate measurements, ensuring the performance of device in practical, real-world scenarios.

Table S1 Comparison of the performance of various on-chip all-optical switches.

Principle	Peak Power	Switching Time	Switching Energy	Switching Contrast	Duty Cycle (%)	Repetition Rate
BFD+TPA/LA [16]	47 μW *	35 ps	0.66 fJ/bit	10 dB	1.4×10^{-2} *	10 MHz $^{p\text{-}p}$
	NA	NA	10.2 fJ/bit	NA	NA	40 Gbps 4-bit pulses
BFD+FCE [17]	10.33 mW *	NA	310 fJ/bit	3 dB	2.4×10^{-1} *	80 MHz $^{p\text{-}p}$
Saturable absorption [18]	167 mW *	260 fs	35 fJ/bit	3.5 dB	2.1×10^{-4} *	10 MHz $^{p\text{-}p}$
$\chi^{(2)}$ nonlinearity [19]	2 W *	46 fs	80 fJ/bit	3 dB	1×10^{-3} *	250 MHz $^{p\text{-}p}$
FCE [20]	5 mW *	30 ps	100 fJ/bit	3 dB	NA	5 Gbps 4-bit pulses
FCE [21]	60 mW *	70 ps	460 fJ/bit	NA	7.7×10^{-3} *	10 MHz $^{p\text{-}p}$
Kerr effect [22]	370 mW *	NA	1124 fJ/bit *	7.7 dB	12.81 *	42.7 Gbps 2^{31}-1 PRBS
Kerr effect [23]	264 mW *	18 ps	720 fJ/bit	NA	2.7×10^{-2} *	100 MHz $^{p\text{-}p}$
ISBT [24]	4.3 W *	1.2 ps	520 fJ/bit	7 dB	9.12×10^{-4} *	76 MHz $^{p\text{-}p}$
**This work**	**7.5 mW**	**9 ps**	**63 fJ/bit**	**8.3 dB**	**33**	**40 Gbps 2^7-1 PRBS (Error-free)**

Notes: NA—not available, BFD—band-filling dispersion, TPA—two-photon absorption, LA—linear absorption, FCE—free-carrier effect, ISBT—inter-sub-band transition, p-p—pump-probe measurement, PRBS—Pseudo-Random Binary Sequence, *—calculated based on available data: The switching energy is defined as the product of the on-chip peak pump power and the effective temporal width of the input pump "1"-bit, while the duty cycle is given by the product of this pulse width and the pulse repetition rate.

…

[16] Nozaki, K. et al. Sub-femtojoule all-optical switching using a photonic-crystal nanocavity. *Nat. Photon*. **4**, 477–483 (2010).

[17] Takiguchi, M. et al. All-Optical InAsP/InP Nanowire Switches Integrated in a Si Photonic Crystal. *ACS Photon*. **7**, 1016-1021 (2020).

[18] Ono, M. et al. Ultrafast and energy-efficient all-optical switching with graphene-loaded deep-subwavelength plasmonic waveguides. *Nat. Photon*. **14**, 37–43 (2020).

[19] Guo, Q. et al. Femtojoule femtosecond all-optical switching in lithium niobate nanophotonics. *Nat. Photon*. **16**, 625–631 (2022).

[20] Moille, G. et al. Integrated all-optical switch with 10 ps time resolution enabled by ALD. *Laser Photonics Rev*. **10**, 409 (2016).

[21] Tanabe,T. et al. Fast all-optical switching using ion-implanted silicon photonic crystal nanocavities. *Appl. Phys. Lett*. **90**, 031115 (2007).

[22] Koos, C. et al. All-optical high-speed signal processing with silicon-organic hybrid slot waveguides. *Nat. Photon*. **3**, 216–219 (2009).

[23] Jason S. et al. Picosecond all-optical switching in hydrogenated amorphous silicon microring resonators. *Opt. Express* **22**, 3797-3810 (2014).

[24] Hu X. et al. Picosecond and low-power all-optical switching based on an organic photonic-bandgap microcavity. *Nat. Photon.* **2**, 185–189 (2008).

Reviewer's Q8:

8. The Authors report error-free performance (BER ≤ 10^-9) beyond 50 fJ/bit with power penalties trending with contrast. Please provide full receiver configuration (detector type, bandwidth, reference clocking), number of bits tested to substantiate "error-free", confidence bounds, bathtub curves/jitter decomposition, and penalties at fixed BER (e.g., 10^-3 pre-FEC and 10^-9 post-FEC). Also clarify whether the probe channel is spectrally filtered tightly enough to suppress pump leakage for all thermal drifts.

Author's A8:

We sincerely thank the reviewer for this constructive comment regarding the experimental substantiation of the reported error-free performance and the associated receiver configuration. We agree that a clear description of the measurement setup and filtering conditions is essential for a rigorous assessment of high-speed BER results.

1. Receiver configuration

The high-speed data generation and BER measurements are carried out using an integrated SHF communication test platform, consisting of a synthesized clock generator (SHF 78210 D), a bit-pattern generator (SHF 12104 A), and an error analyzer (SHF 11104 A), as shown in Fig. **R16**. The synthesized clock generator provides a low-jitter reference clock that synchronizes both the transmitter and receiver, ensuring deterministic timing and accurate bit-by-bit error analysis. The bit pattern generator generates a PRBS sequence of length $2^7 - 1$ at the target symbol rate, which is operated in a cyclic mode to provide a continuous data stream for driving the modulator. Meanwhile, the error analyzer performs real-time BER counting by synchronizing the detected electrical signal with the internally generated reference PRBS pattern. BER counting is initiated only after successful pattern acquisition and stable lock are achieved, ensuring reliable synchronization prior to real-time error counting.

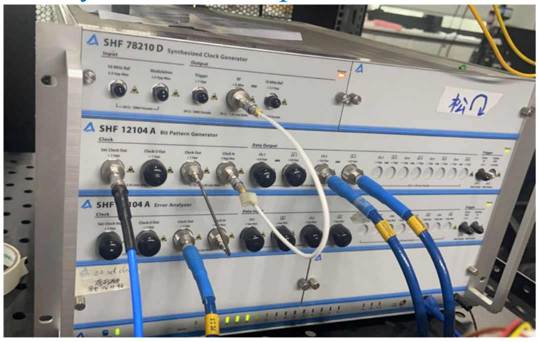

Fig. R16. Experimental setup for synthetic clock generator, bit pattern generator, and error analyzer.

On the receiver side, the switched probe signal is detected using a high-speed photodetector (XPDV2120R, 40-GHz electrical bandwidth), followed by broadband electrical amplification and direct connection to the SHF error analyzer, as shown in Fig. **R17**. By adjusting a variable optical attenuator (VOA) placed before the photodetector (PD), the BER is measured as a function of the received optical power at the PD, as shown in Fig. **4b** of the manuscript. The overall electrical bandwidth of the detection chain is sufficient to support the maximum tested symbol rate (40 GBaud), ensuring that the measured BER performance is not limited by receiver bandwidth.

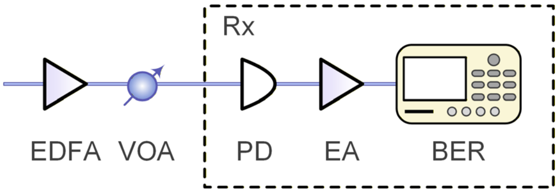

Fig. R17. Experimental setup for the BER measurements.

2. Number of bits tested and confidence bounds

The PRBS sequence (length 2^7-1) is generated in a continuous cyclic mode and measured over an extended duration. At a symbol rate of 40 GBaud and a measurement time of 2 s, a total of more than 8×10^{10} bits are analyzed. With zero errors observed, Poisson statistics yield an upper BER bound of 3.75×10^{-11} at a 95% confidence level, which is well below the commonly accepted error-free criterion of 10^{-9}, thereby substantiating the reported error-free operation.

3. Bounds bathtub curves/jitter decomposition

To provide a quantitative bound on timing jitter, we further analyze the experimentally acquired eye-diagram data using a statistical approach. As illustrated in Fig. **R18**, the measured eye diagram (left) is first processed by extracting all waveform samples within the central 20% of the unit interval, corresponding to the steady-state region of the eye opening. The probability density distribution of the signal amplitude is then obtained by forming a histogram over these samples (right). The most probable values at the "0" and "1" logic levels are identified from the peaks of the corresponding amplitude distributions. Based on these extracted logic levels, the 50% decision threshold is defined, and the temporal region over which the rising and falling edges overlap at this level is evaluated. The width of this overlap is taken as a quantitative measure of the total eye-diagram jitter under the given operating condition. This metric captures the combined influence of timing jitter, amplitude noise, inter-symbol interference, and pattern-dependent distortions present in the experimental system.

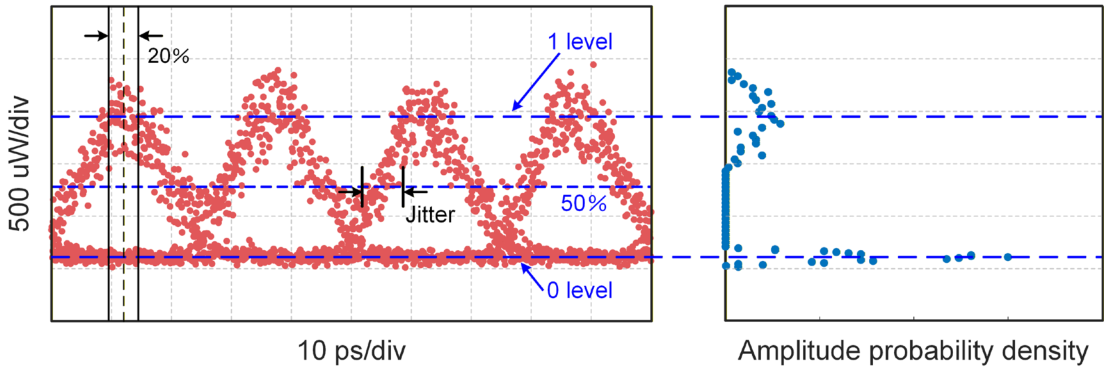

Fig. R18. Extraction of eye-diagram timing jitter from measured eye diagrams.

Using this method, the back-to-back input pump signal exhibits an eye-diagram jitter of 5.47 ps, which reflecting the intrinsic jitter of the experimental link and the quality of the original signal. Applying the same analysis to the output eye diagrams shown in inset (i)-(iii) of Fig. **4a**, the extracted effective eye-diagram jitters are 9.92 ps, 7.42 ps, and 7.37 ps, respectively. This evolution closely follows the trend of switching contrast with increasing pump power: from case (i) to (ii), both the switching contrast and signal quality improve markedly, while from (ii) to (iii), both quantities approach saturation. These results indicate that the Kerr-based switching process does not introduce excessive additional timing jitter that would significantly degrade system

performance relative to the baseline. Moreover, the output signal quality improves and eventually stabilizes as the switching contrast approaches saturation.

4. Power penalties

Regarding the BER thresholds and power-penalty definition, we clarify that no forward error correction (FEC) is employed in the present experiments. All reported BER values therefore correspond to raw (pre-FEC) bit-error rates measured directly by the error analyzer. The power penalty is defined at a fixed BER level of 10^{-9}, which is commonly adopted as the criterion for error-free operation in signal-processing demonstrations. Specifically, BER-versus-received-power curves are measured for both the switched probe signal and a back-to-back reference signal under otherwise identical receiver conditions. The power penalty is then obtained as the difference in received optical power at the photodetector required to achieve $\mathrm{BER} = 10^{-9}$.

5. Pump and probe spectral isolation

At the output, the detected probe signal is filtered out by a bandpass filter, resulting in an output power of approximately -23 dBm, which is then amplified by an EDFA. A second bandpass filter is employed to suppress amplified spontaneous emission noise and further improve signal quality, as shown in Fig. **R19**. Owing to the sufficiently large wavelength separation between the pump and probe signals ($\approx 23\ nm$), the employed filtering scheme fully accommodates the probe bandwidth while providing strong suppression of residual pump leakage.

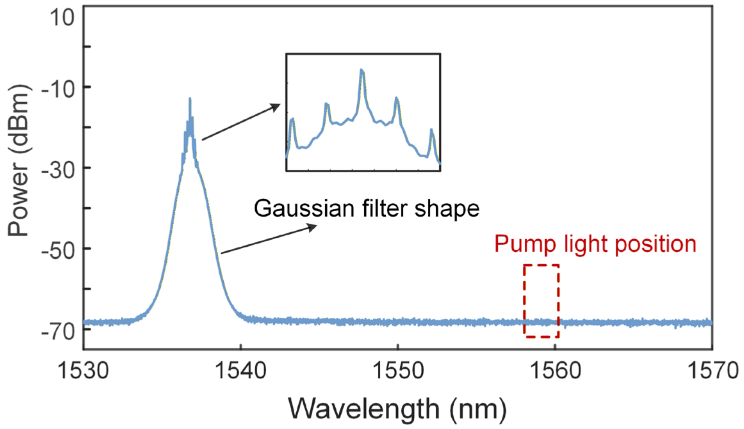

Fig. R19. Output spectrum after the filter-amplify-filter.

We believe that these additional clarifications and quantitative details fully address the reviewer's concerns and further strengthen the rigor of the reported error-free switching performance. A detailed analysis is provided in Section **S9** of the revised Supplementary Information.

(Line 517 in revised Supplementary Information)

S9. Experimental Setup for Optical Measurement

The high-speed data generation and BER measurements are carried out using an integrated SHF communication test platform, consisting of a synthesized clock generator (SHF 78210 D), a bit-pattern generator (SHF 12104 A), and an error

analyzer (SHF 11104 A). The synthesized clock generator provides a low-jitter reference clock that synchronizes both the transmitter and receiver, ensuring deterministic timing and accurate bit-by-bit error analysis. The bit pattern generator generates a PRBS sequence of length $2^7 - 1$ at the target symbol rate, which is operated in a cyclic mode to provide a continuous data stream for driving the modulator. Meanwhile, the error analyzer performs real-time BER counting by synchronizing the detected electrical signal with the internally generated reference PRBS pattern. BER counting is initiated only after successful pattern acquisition and stable lock are achieved, ensuring reliable synchronization prior to real-time error counting.

On the receiver side, the switched probe signal is detected using a high-speed photodetector (XPDV2120R, 40-GHz electrical bandwidth), followed by broadband electrical amplification and direct connection to the SHF error analyzer, as shown in Fig. **S21**. By adjusting a variable optical attenuator (VOA) placed before the photodetector (PD), the BER is measured as a function of the received optical power at the PD, as shown in Fig. **4b**. The overall electrical bandwidth of the detection chain is sufficient to support the maximum tested symbol rate, ensuring that the measured BER performance is not limited by receiver bandwidth.

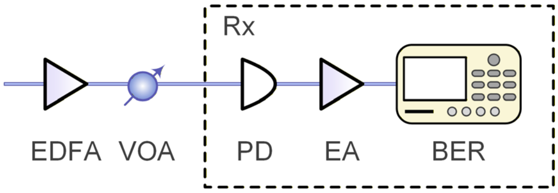

Fig. S21. Experimental setup for the BER measurements.

The PRBS sequence (length $2^7 - 1$) is generated in a continuous cyclic mode and measured over an extended duration. At a symbol rate of 40 GBaud and a measurement time of 2 s, a total of more than 8×10^{10} bits are analyzed. With zero errors observed, Poisson statistics yield an upper BER bound of 3.75×10^{-11} at a 95% confidence level, which is well below the commonly accepted error-free criterion of 10^{-9}, thereby substantiating the reported error-free operation.

Reviewer's Q9:

9. The effective nonlinear coefficient is estimated at 2952 $m^{-1}W^{-1}$ (31× a strip Si waveguide) and n2 for MEH-PPV is stated as "about 20× silicon." Provide the exact n2 value used at 1550 nm, its dispersion, and experimental validation on your structures (z-scan on films, self-/cross-phase modulation on straight SOH waveguides). Without a direct measurement on the fabricated devices, it is difficult to disentangle n2, mode volume, and Q-loading contributions. Include error bars from uncertainties in field overlap and effective area.

Author's A9:

We sincerely thank the reviewer for this insightful and technically important question regarding the effective nonlinear coefficient, the value and dispersion of the nonlinear refractive index n_2, and the disentanglement of material and cavity contributions. The

effective nonlinear coefficient reported in this work, $\gamma_{eff} = 2952\ \mathrm{m^{-1}W^{-1}}$, corresponds to an enhancement of approximately $31\times$ compared to a standard silicon strip waveguide. This enhancement should not be interpreted as arising solely from a larger material nonlinear refractive index, but rather from the combined effect of the polymer nonlinearity and strong optical confinement in the slot nanocavity. As explicitly shown in Eq. (S30) of the Supplementary Information, the nonlinear coefficient scales as

$$\gamma = \frac{2\pi n_2}{\lambda A_{eff}}$$

where λ is the wavelength of the incident light, n_2 is the nonlinear refractive index of the material, and A_{eff} represents the effective mode area. While the Kerr coefficient of MEH-PPV is approximately $20\times$ that of crystalline silicon at telecom wavelengths (see below), the additional enhancement in γ_{eff} primarily originates from the drastic reduction of the effective mode area enabled by the polymer-filled slot nanocavity. Specifically, as detailed in Section **S6** of the Supplementary Information, the effective mode area at the center of the 80-nm-wide slot nanocavity is estimated to be $0.125\ \mathrm{\mu m^2}$, which is approximately $1.5\times$ smaller than that of a standard silicon strip waveguide ($\sim 0.189\ \mathrm{\mu m^2}$).

The Kerr nonlinear refractive index n_2 of MEH-PPV used in this work is not a fitted parameter, but is taken from direct experimental measurements performed in our previously published work [*Photon. Res.* **10**, 50–58 (2022)], where detailed Z-scan characterization is carried out on spin-coated MEH-PPV films at telecom wavelengths. In those measurements, a standard Z-scan setup is employed using femtosecond pulses at 1550 nm (converted from a 1030-nm source via an optical parametric amplifier). Closed-aperture scans are used to extract the nonlinear refractive index, while open-aperture scans are simultaneously performed to assess nonlinear absorption. A clear valley–peak signature is observed in the closed-aperture configuration, confirming positive Kerr nonlinearity (self-focusing), whereas the open-aperture traces remain flat, indicating negligible nonlinear absorption. From these measurements, the Kerr coefficient of MEH-PPV at 1550 nm is determined to be $(8.0 \pm 0.4) \times 10^{-17}\ \mathrm{m^2/W}$, corresponding to approximately $20\times$ the Kerr coefficient of crystalline silicon at the same wavelength. Despite this, we have conducted additional Z-scan measurements on a new 10 mm×10 mm MEH-PPV film with a quartz substrate. For these tests, three different points on the film are selected. We initially use the lowest possible power, with the focused intensity approximately 29 GW/cm^2, to measure n_2 and nonlinear absorption. The closed-aperture scan clearly shows a valley-peak characteristic, confirming positive Kerr nonlinearity (self-focusing), as shown in Fig. **R20**. The measured n_2 values are $3.09\times 10^{-16}\ \mathrm{m^2/W}$, $1.93\times 10^{-16}\ \mathrm{m^2/W}$, and $9.22\times 10^{-17}\ \mathrm{m^2/W}$, respectively, with the variations mainly attributed to film thickness nonuniformities and potential additional testing errors due to the low light power used. These discrepancies, when compared to our previously reported value of approximately $(8.0 \pm 0.4) \times 10^{-17}\ \mathrm{m^2/W}$, are likely due to several factors, including differences in the material batch, the variation in film thickness, as well as potential variations in the experimental setup, such as the choice of lenses and objective lenses, which could result

in different beam spot sizes and intensities. Furthermore, the curve remains flat in the open-aperture scan, confirming that nonlinear absorption is negligible, as shown in Fig. **R21**. Since the switching mechanism in our experiments relies on cross-phase modulation (XPM), the phase-matching conditions are not as sensitive to dispersion, due to inherently satisfied phase matching condition. Furthermore, in both our prior four-wave mixing (FWM) experiments [*Photon. Res.* **10**, 50–58 (2022)] and the current Kerr-based switching experiments, the interacting wavelengths are closely spaced within the telecom C-band. Under these conditions, the impact of dispersion on the effective Kerr interaction is negligible.

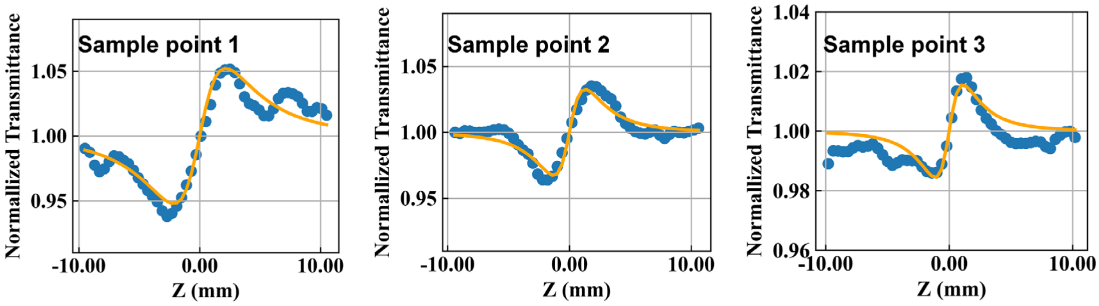

Fig. R20. Closed aperture Z-scan measurement of the MEH-PPV film for nonlinear refractive index n_2 characterization.

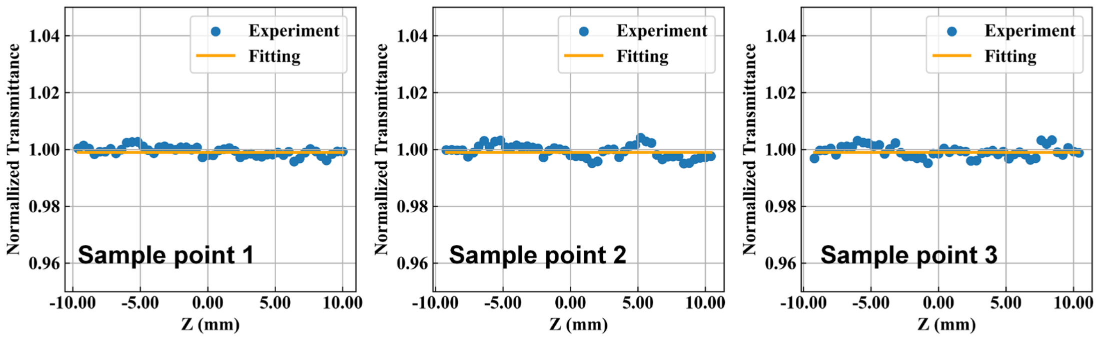

Fig. R21. Open aperture Z-scan measurement of the MEH-PPV film for absorption characterization.

With respect to separating material and cavity contributions, we emphasize that: (1) The nonlinear refractive index n_2 is independently determined from Z-scan measurements, with an experimental uncertainty of $\pm 0.4 \times 10^{-17}$ m^2/W. (2) The effective mode area A_{eff}, mode volume, and field overlap factors are calculated directly from full-wave FDTD simulations based on the measured device geometry. However, in practice, factors such as variations in the polymer spin-coating process, slight deviations in wavelength detuning, and other fabrication-related effects may introduce some discrepancies between the simulated and actual values. While it is challenging to precisely quantify these discrepancies, we address their potential impact through a simulated impact analysis of geometric variations (fabrication-robustness

analysis) and experimental validation via device-to-device statistics, as detailed in **Author’s A6**.

In summary, the effective nonlinear coefficient enhancement arises from the combined effect of an experimentally measured large Kerr nonlinearity of MEH-PPV and strong sub-wavelength optical confinement in the slot nanocavity, rather than from an anomalously large material nonlinearity alone. The values of n_2, their uncertainties, and the role of mode area and cavity loading are now explicitly clarified and supported by both prior experimental validation and the present electromagnetic simulations.

Reviewer's Q10:

10. The Supplement shows excellent SEMs down to 40-nm slots fabricated by NLD etching. Please include statistical distributions of as-fabricated slot width, sidewall roughness (Rq), and cavity resonance wavelengths across the die/wafer, and correlate these to the optical metrics (Q, ER, IL) and the achieved switching energy. Report yield of devices meeting the 40-Gbaud spec and how sensitive performance is to polymer thickness variation and infiltration completeness.

Author's A10:

We sincerely thank the reviewer for this important comment, which addresses the practical fabrication variability and robustness of the proposed hybrid silicon-organic (SOH) nanocavity platform. We would like to clarify that the devices reported in this work are fabricated on centimeter-scale SOI chips using an in-house nanofabrication process, rather than through a wafer-scale industrial process. Accordingly, device optimization is achieved through systematic on-chip parameter scanning in conjunction with process optimization, which together serve to mitigate residual fabrication-induced deviations that remain unavoidable in a non-wafer-scale fabrication environment. Specifically, on each fabricated chip, we intentionally introduce controlled design variations in key geometrical parameters, including the air-hole radius, slot width, cavity-bus waveguide gap, and waveguide width. This strategy enables compensation of fabrication-induced deviations and allows us to experimentally identify devices with optimized optical performance. Therefore, the devices characterized in the main text are selected from this parameter space as representative high-performance realizations, rather than random samples.

After polymer spin coating, direct SEM measurement of the slot width becomes challenging due to the significantly reduced surface conductivity of the hybrid structure. Instead, we estimate the actual slot width through a calibrated indirect approach. Specifically, the test waveguides with designed slot widths from 20 nm to 250 nm are fabricated on the same chip without polymer infiltration and characterized by SEM to extract the systematic fabrication deviation between the designed and realized slot widths, as summarized in Fig. **R22**. For the devices characterized in the main text, a slot-width compensation of approximately -20 nm is employed in the layout. By comparing this compensation value with the empirically extracted fabrication deviation shown in Fig. **R22**, we infer that the effective slot width of the devices used in this work deviates from the nominal design value (40 nm–80 nm) by approximately -10 to -5 nm.

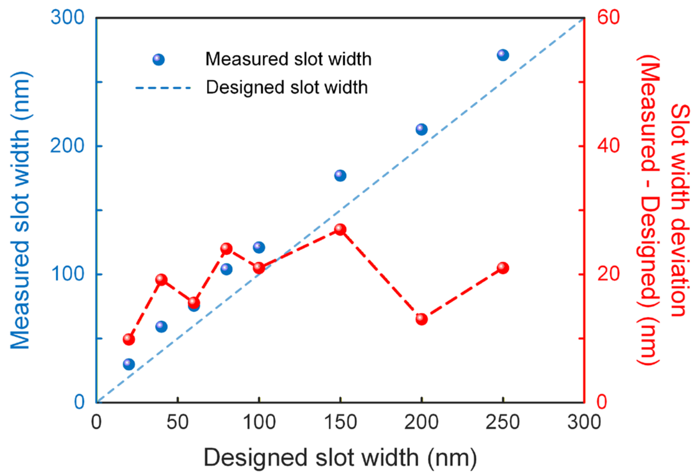

Fig. R22. Comparison between designed and measured slot widths (left axis) and width deviation (right axis).

Regarding sidewall roughness, direct atomic force microscopy (AFM) measurement of the slot sidewalls is technically challenging due to the extremely high aspect ratio of the 40-nm-wide slots and the limited probe diameter (~14 nm) available in our facility (Nanosurf Flex-Mount system). To quantitatively assess the etching quality, we therefore extract the sidewall roughness from high-resolution SEM images using a grayscale-gradient-based edge-detection method. Specifically, the root-mean-square (RMS) deviation of the extracted edge profile is used as an effective measure of Rq, defined as:

$$RMS = \sqrt{\frac{1}{N}\sum_{i=0}^{N}|x(i) - \bar{x}|^2}$$

where $x(i)$ denotes the edge position obtained at the $i - th$ pixel, $\bar{x}$ represents the corresponding best-fit linear trend that removes slow geometric variations, and N is the number of pixels within the evaluation window. Using this method, we extract effective roughness values for different feature sizes, as summarized in Fig. **R23**. The results indicate that the estimated roughness is approximately 2.42 nm for 80-nm slots, 2.92 nm for 60-nm slots, and 3.36 nm for 40-nm slots. The observed increase in roughness with decreasing slot width is attributed to the higher aspect ratio, which hampers the removal of etching by-products.

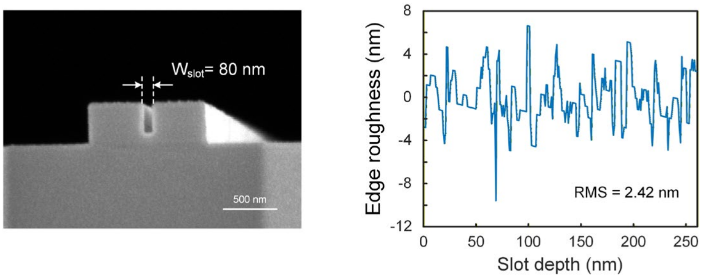

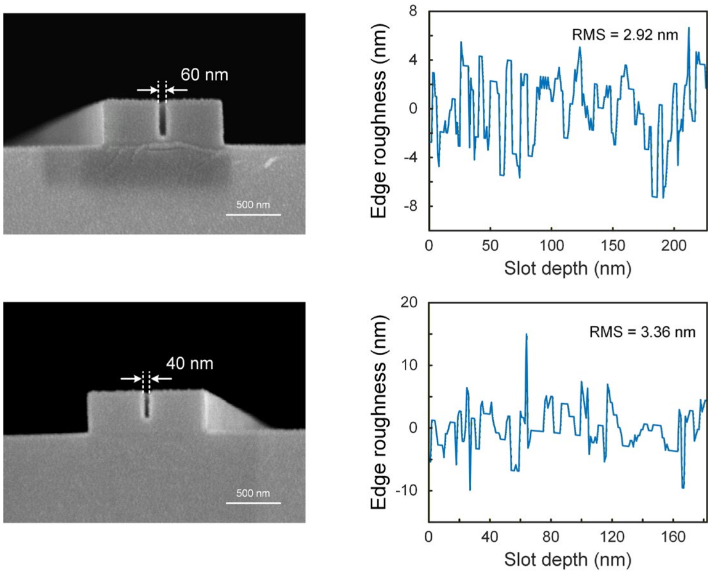

Fig. R23. The effective roughness values for different feature sizes.

The statistical distribution of cavity resonance wavelengths is evaluated as described in **Author's A6** by fabricating twelve nominally identical devices on the same chip using identical process conditions. Statistical analysis of the measured transmission spectra indicates that the resonance wavelength of Fano mode 1 varies from approximately 1530 nm to 1540 nm, while the loaded Q-factor spans a range of 1500-2500. These variations are primarily attributed to non-uniformities in polymer spin coating, etching-induced dimensional fluctuations, and local thickness variations of the SOI top silicon layer (210-230 nm), all of which are common in hybrid silicon-organic nanophotonic platforms. Despite these variations, all measured devices consistently exhibit a clear Fano line shape and well-defined cavity resonances, indicating that the formation of the Fano response is tolerant to realistic fabrication variability.

(Line 418 in revised Supplementary Information)

…

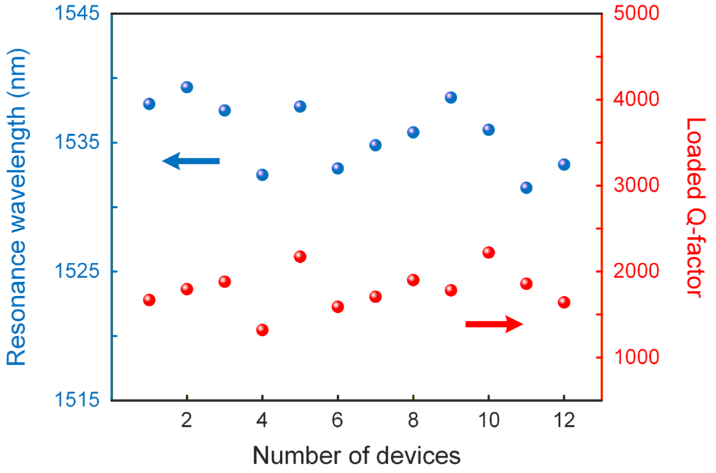

Fig. S14. Statistical distribution of the resonance wavelength (left axis) and loaded Q-factor (right axis) for identical devices fabricated on the same chip.

Rather than exhibiting a strict one-to-one correspondence, the influence of fabrication variations on Q-factor, extinction ratio (ER), insertion loss (IL), and switching energy manifests through well-understood physical trends. In particular, variations in slot width primarily affect the effective refractive index and optical mode distribution within the nanocavity, leading to systematic shifts of the resonance wavelength. At the same time, an increase in slot width is found to result in a moderate enhancement of the loaded Q-factor, as also confirmed by full-wave simulations in **Author's A6**. By contrast, sidewall roughness directly impacts the intrinsic cavity loss and therefore plays a dominant role in determining the loaded Q-factor, which in turn critically affect the achievable ER. As described by Eq. (S7) in the Supplementary Information, for a fixed coupling condition, an increase in the intrinsic Q-factor significantly enhances the Fano resonance contrast, which is critical for low-energy, high-contrast all-optical switching. Consequently, further optimization of the fabrication process—such as reducing sidewall scattering through annealing, thermal oxidation, or optimized etching processes—provides a clear and practical pathway to further enhance the intrinsic Q-factor and improve switching performance.

With respect to switching performance, our analysis indicates that polymer infiltration completeness is the most critical factor, whereas moderate variations in polymer thickness have a comparatively weak impact. FDTD simulations show that realistic thickness variations lead only to minor changes in mode confinement and nonlinear overlap, while incomplete infiltration of the slot region results in pronounced resonance wavelength shifts and severe degradation of the extinction ratio. These signatures are readily observable in the measured transmission spectra during the on-chip parameter scanning process and are therefore used as practical screening criteria for device selection. From a fabrication standpoint, the devices intentionally employ a slightly over-etched slot geometry, such that the slot is etched marginally into the underlying buried oxide (BOX) layer. As a result, during polymer spin coating, any residual unfilled or trapped air regions (Supplementary Information, Fig. **S12**, Step 7) preferentially occupy the over-etched region originally belonging to the silicon dioxide rather than the silicon core region. Since the optical mode is predominantly confined within the silicon and polymer-filled slot region, partial voids located within the over-etched BOX region have a comparatively weak influence on mode confinement, scattering loss, and nonlinear interaction strength. Consequently, incomplete filling in these regions has a limited impact on the overall optical performance of the device. Moreover, because the slots are connected to the air-hole lattice, a post-spin-coating vacuum-assisted infiltration step (Supplementary Information, Fig. **S12**, Step 8) effectively removes trapped air and promotes complete polymer filling of the slot region via pressure-driven flow. As a result, multiple devices on the same chip consistently meet the 40-GBaud switching requirement. In addition to the single-device demonstrations discussed in the main text, multi-channel switching operation is also achieved (Fig. **R24** and **R25**), further supporting the practical yield and robustness of the proposed platform.

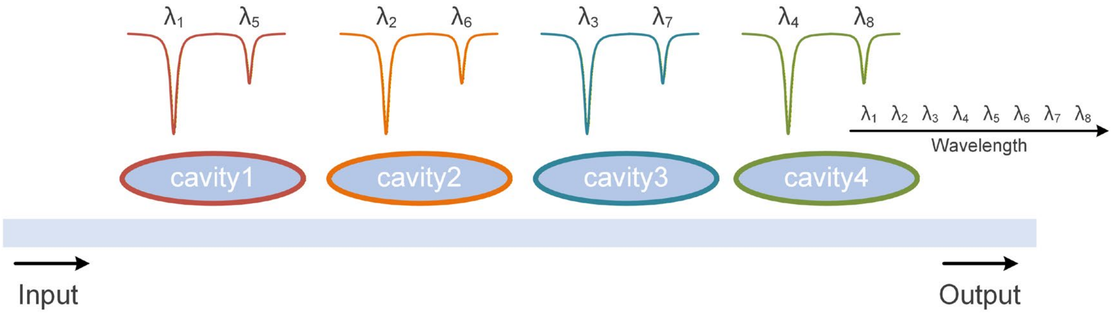

Fig. R24. Schematic diagram of the multi-channel SOH Kerr all-optical switch.

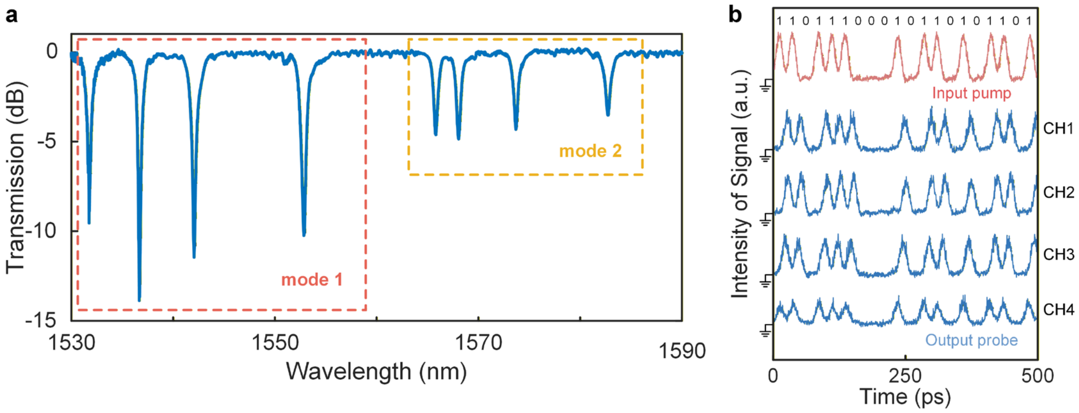

Fig. R25. 4×40 GBaud all-optical switching. a Experimental measured transmission spectrum. **b** Input pump pulse train (red) and output probe pulse train from different channels (blue).

Here, my minor comments:

Reviewer's MQ1:

1. Define all symbols at first use and maintain consistent notation between main text and SI (e.g., Q_i/Q_e/Q_L, FE, ER, V_eff, γ, κ, δ, τ_ph).

Author's MA1:

We thank the reviewer for this helpful suggestion. In the revised manuscript and Supplementary Information, we have carefully reviewed all symbols and now explicitly define each symbol at its first occurrence. In addition, we have unified the notation between the main text and the SI to ensure full consistency, including Q_i, Q_e, Q_L, field enhancement factor (FE), extinction ratio (ER), mode volume (V), effective nonlinear coefficient (γ_{eff}), coupling coefficient (κ), phase shift (δ) and photon lifetime (τ_{ph}).

Reviewer's MQ2:

2. Figures: in Fig. 2b, label the ripple period (1.4 nm) on the plot; in Fig. 3b inset, annotate the 40-GHz sidebands; ensure all axes have units and all spectra indicate resolution bandwidths. Eye diagrams in Fig. 4a would benefit from identical scaling for fair visual comparison.

Author's MA2:

We thank the reviewer for these helpful and detailed suggestions regarding figure clarity and consistency. We have carefully revised the figures accordingly in the revised manuscript. Specifically:

1. In Fig. **2b**, we have added an explicit label indicating the ripple period of 1.4 nm directly on the spectrum.

(Line 491 in revised manuscript)

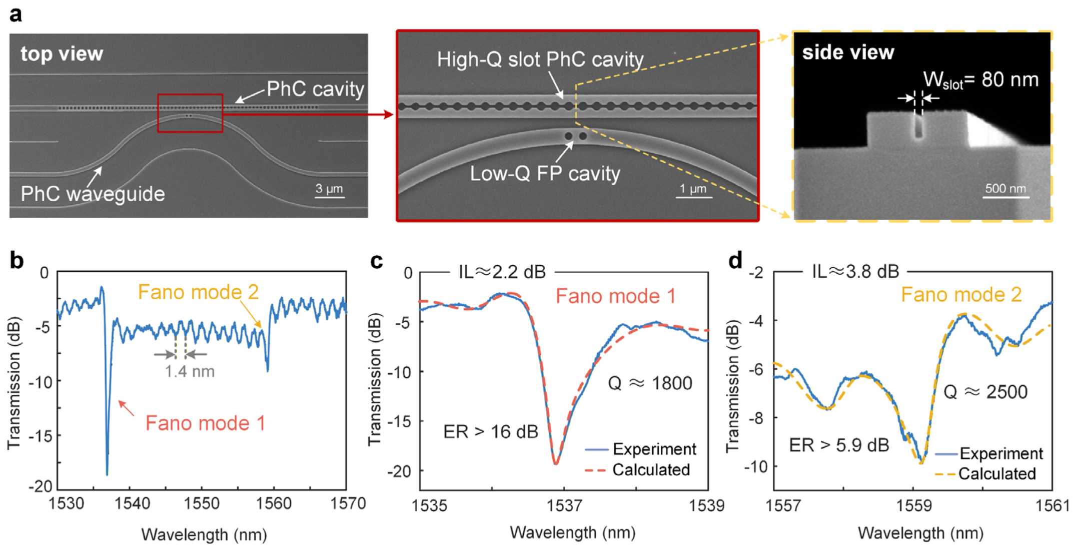

Fig. 2. SEM images and transmission spectra of the fabricated samples. …

2. In the inset of Fig. **3b**, the 40-GHz modulation sidebands are now clearly annotated to guide the reader. We have checked all figures to ensure that every axis includes appropriate units, and that all optical spectra explicitly indicate the corresponding resolution bandwidths.

(Line 506 in revised manuscript)

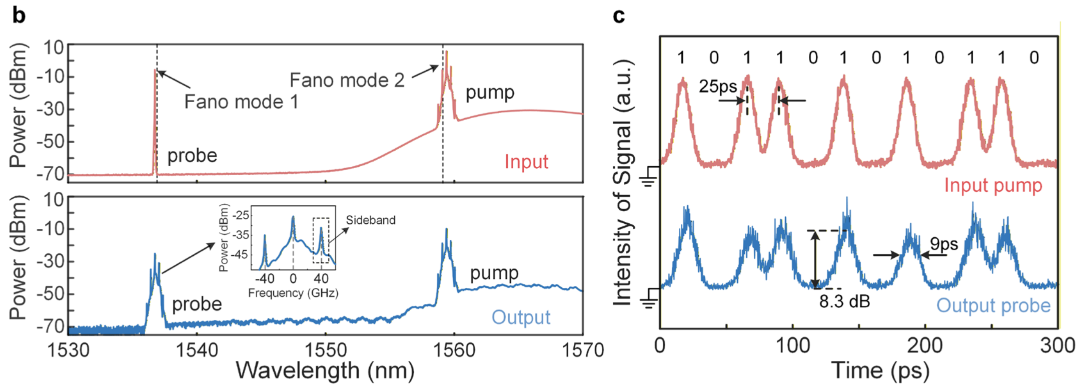

Fig. 3. Demonstration of two-mode all-optical Kerr switching. …**b** Input spectrum (red) and the output spectrum after the device (blue), measured with a resolution bandwidth of 0.02 nm. The black dashed lines in the upper panel indicate the resonant wavelengths of Fano mode 1 and Fano mode 2, respectively. The inset in the bottom panel shows the modulated probe light with distinct modulation sidebands. The input pump energy is 63 fJ/bit corresponding to a peak power of

7.5 mW. …

(Line 544 in revised Supplementary Information)

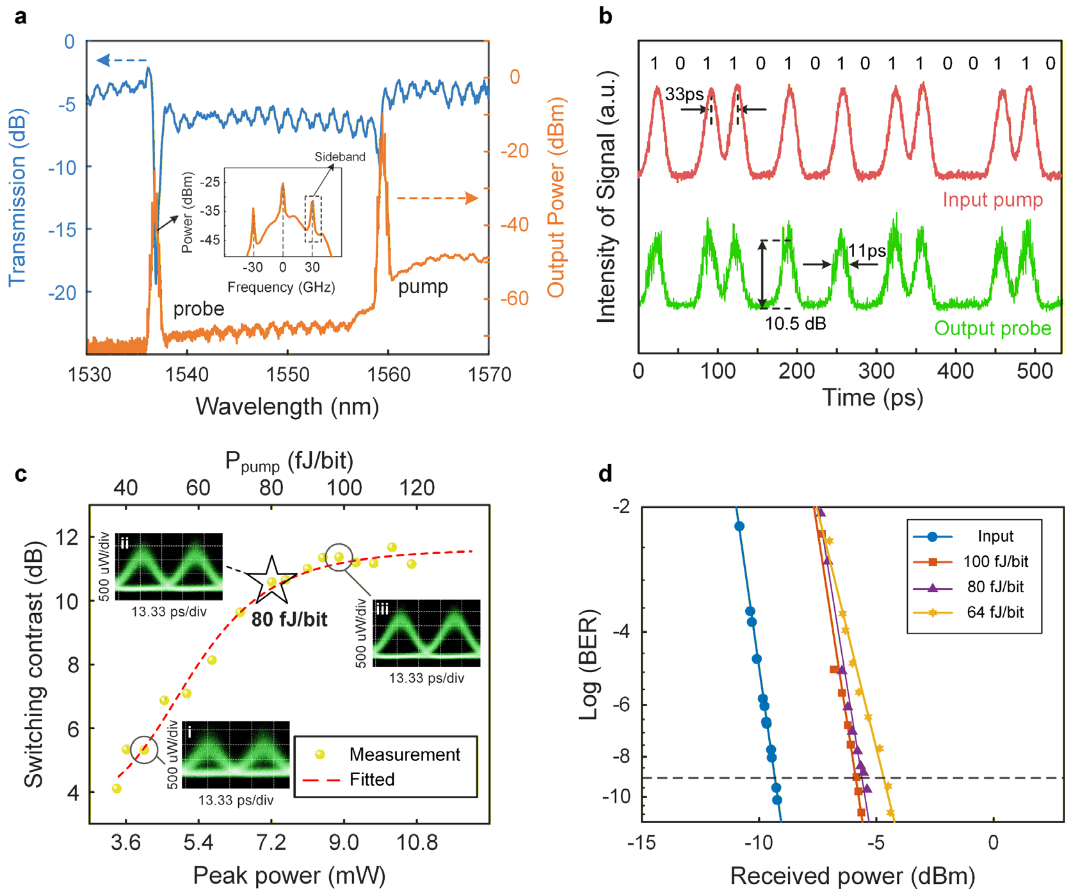

Fig. S22. Femtojoule 30 GBaud all-optical switching with error-free performance. a Transmission spectrum (blue) and the output spectrum obtained after the device (orange), measured with a resolution bandwidth of 0.02 nm. The inset shows the modulated probe light with distinct modulation sidebands. The input pump light energy is 80 fJ/bit corresponding to a peak power of 7.2 mW. …

3. In the revised manuscript, the eye diagrams in Fig. **4a** have been replotted using identical horizontal and vertical scales to enable fair visual comparison across different operating conditions. In addition, the grid lines have been made more clearly visible, and the corresponding signal amplitude levels are explicitly annotated in each panel. These revisions ensure consistent scaling and improve the readability and quantitative comparability of the eye diagrams.

(Line 521 in revised manuscript)

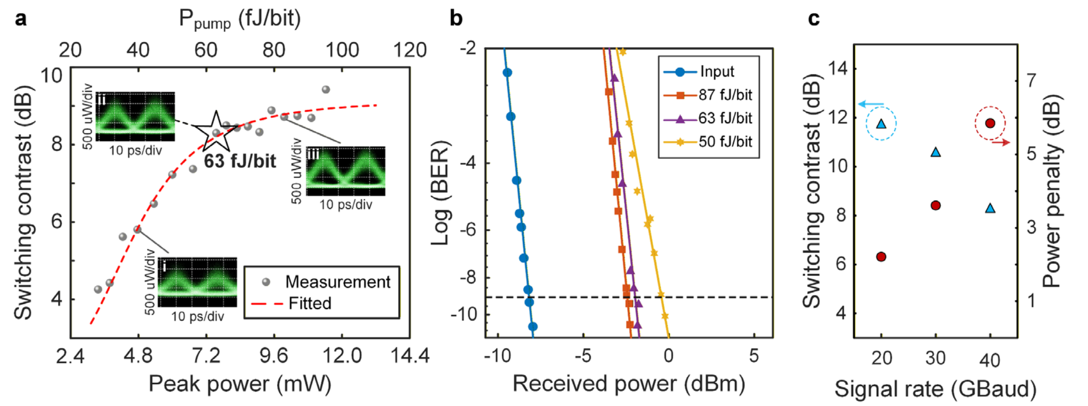

Fig. 4. Femtojoule 40 GBaud all-optical switching with error-free performance. …

Reviewer's MQ3:

3. Clarify whether insertion loss values are on-chip (excluding grating/edge coupling) or end-to-end. If on-chip, report coupling loss separately for completeness.

Author's MA3:

We thank the reviewer for pointing out the need for clarification. We confirm that the insertion loss (IL) values reported in the manuscript refer to the on-chip loss of the device and exclude the coupling loss associated with the input/output interfaces. To avoid any ambiguity, we have now explicitly clarified this definition in the revised manuscript. In addition, we have provided the SEM images and transmission spectrum of the grating coupler in Section **S7** of the revised Supplementary Information. Specifically, the device employs vertical grating couplers at both the input and output, with a coupling loss of approximately -6 dB/facet. These additions allow readers to clearly distinguish between the intrinsic on-chip performance of the device and the external coupling loss.

(Line 492 in revised manuscript)

…

Fig. 2. SEM images and transmission spectra of the fabricated samples. … **c** Experimental (blue solid line) and calculated (red dashed line) transmission spectrum of Fano mode 1. The calculated loaded Q-factor, extinction ratio (ER), and insertion loss (IL) for Fano mode 1 are 1.8×10^3, 16 dB, and 2.2 dB, respectively. **d** Experimental (blue solid line) and calculated (yellow dashed line) transmission spectrum of Fano mode 2. The calculated loaded Q-factor, ER, and IL for Fano mode 2 are 2.5×10^3, 5.9 dB, and 3.8 dB, respectively. All insertion loss values refer to on-chip loss, excluding grating-coupler losses.

(Line 421 in revised Supplementary Information)

…

In the experiment, light is coupled into and out of the chip using photonic crystal (PhC) grating couplers, with a measured coupling loss of approximately -6 dB/facet, as shown in Fig. **S15**. The intrinsic on-chip insertion loss (IL) of the device, excluding

grating-coupler losses, is approximately -2 dB to -4 dB (as shown in Fig. **2**), which mainly originates from mode mismatch between the bus waveguide and the slot PhC nanobeam cavity. In future implementations, the overall optical throughput could be improved by optimizing the coupling efficiency and reducing the intrinsic insertion loss, for example through improved mode matching or by adopting edge-coupling schemes instead of vertical grating couplers.

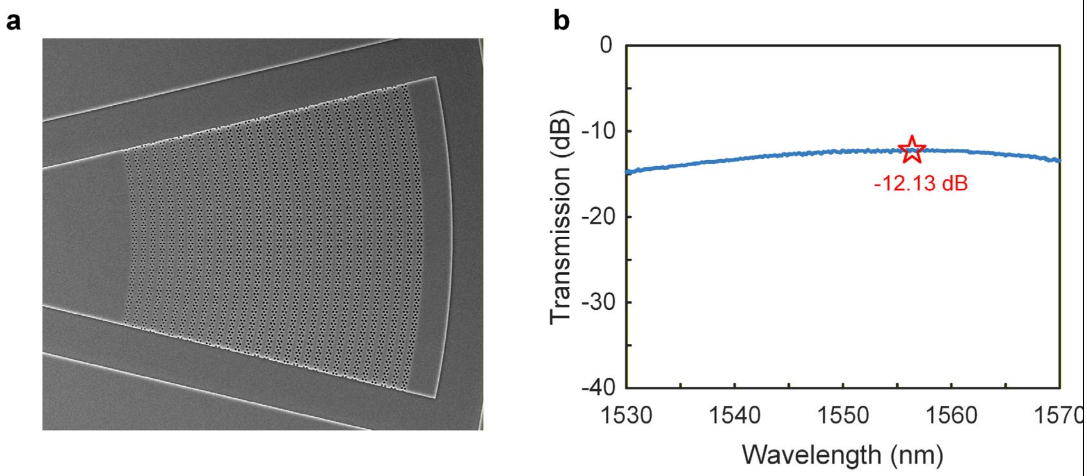

Fig. S15. SEM images and transmission spectrum of the grating coupler.

Reviewer #3

Reviewer's comment:

In the presented paper, the authors experimentally demonstrated nonlinear optical switching with both low peak pump power of 7.5 mW and high duty cycle of 33% for 40 Gbps signals. The required peak power of 7.5 mW is two orders of magnitude lower than previously reported Kerr switches, showing the technological advancement of the proposed device. The manuscript is well constructed, and the results are clearly shown. I would like to recommend publication of the presented paper if the following comments are addressed.

Author's reply:

We are grateful to the reviewer for the positive evaluation of our work and for acknowledging the technological advances demonstrated in this study. We appreciate the constructive comments and have responded to each point thoroughly to further improve the manuscript.

Reviewer's Q1:

1. Throughout the experiment section, the transmission quality of the probe light is measured. However, the motivation for measuring the transmission quality of the probe light is not shown clearly. This experiment might be connected to the optical wavelength conversion, but it should be clearly stated. Moreover, the required performance metrics (such as power penalty and so on) for this application would be required to be clearly shown together with the experimental results.

Author's A1:

We sincerely thank the reviewer for raising this important point and for requesting clarification of the motivation behind measuring the transmission quality of the probe signal. We believe the confusion may stem from a misunderstanding of the role of the probe light in our device operation. In our Kerr-based all-optical switching scheme, the pump light serves as the control signal, while the transmission state of probe light is directly modulated by the pump through cross-phase modulation (XPM), as shown in Fig. **R26**. From a system-level perspective, the probe is therefore the information-bearing channel, and its transmission quality directly reflects the effectiveness of the all-optical switching operation. Therefore, evaluating the transmission quality of the probe is essential for quantifying the practical switching performance of the device, rather than being associated with conventional wavelength conversion based on four-wave-mixing (FWM). It should be further noted that the XPM effect induces a phase shift that directly modulates the input signal without generating new frequency components, which leads to relatively higher output power compared to FWM. In our experiments, the on-chip probe power after switching remains comparable to the input continuous-wave (CW) probe power, indicating that the XPM process itself does not intrinsically result in significant power loss.

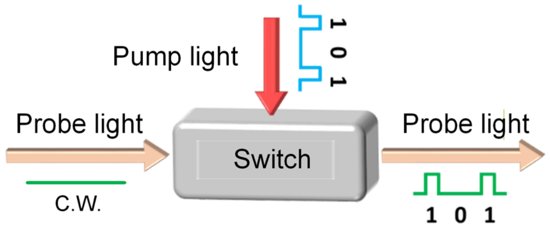

Fig. R26. Schematic diagram of all-optical switching.

Regarding the performance metrics, we fully agree that parameters such as power penalty are important for assessing practical applicability. We would like to clarify that these metrics have indeed been reported in the manuscript—for example, the measured power penalty is provided in Fig. **4c**, together with the trends in switching contrast and power penalty at different signal rates. As the signal rate decreases from 40 GBaud to 20 GBaud, the switching contrast monotonically improves by 3.5 dB (from 8.3 dB to 11.8 dB), while the power penalty is concomitantly reduced by 3.6 dB (from 2.2 dB to 5.84 dB). This improvement arises from the relaxation of constraints imposed by the finite optical bandwidth of the nanocavity, consistent with the fundamental trade-off between bandwidth and operation speed.

Reviewer's Q2:

2. In the results of Fig. 4b, the signal received power reaches 0 dBm. As compared to the peak pump power, this signal power might be large enough to cause other nonlinear effects such as SPM. Could the authors comment on this? Also, it would be helpful for the readers if any nonlinear degradation of signals were observed during the experiments.

Author's A2:

We sincerely thank the reviewer for raising this important point regarding the apparent signal power level in Fig. **4b**. We first clarify that the 0 dBm probe power shown in Fig. **4b** corresponds to the optical power received at the photodetector after external optical amplification, rather than the optical power inside the nanocavity. In the experiment, the probe signal emerging from the chip is first spectrally isolated by a bandpass filter to suppress pump leakage, resulting in an optical power of approximately -23 dBm at the output. This weak probe signal is then amplified by an erbium-doped fiber amplifier (EDFA) to reach the power level required for reliable BER measurement. A second bandpass filter is subsequently employed to suppress amplified spontaneous emission (ASE) noise and further improve signal quality, as shown in Fig. **R27**. The reported received power therefore reflects the photodetector input after amplification, rather than the on-chip probe power. Importantly, the probe power launched into the device is approximately -5 dBm, which is significantly lower than the pump power. Under these conditions, the nonlinear Kerr phase shift experienced by the probe is dominantly induced by cross-phase modulation (XPM) from the pump, while self-phase modulation (SPM) and other parasitic nonlinear effects associated with the probe itself are negligible. We have updated the figure caption to include this information and

clarify the experimental setup, showing explicitly that the high received power results from external amplification rather than from on-chip nonlinear processes.

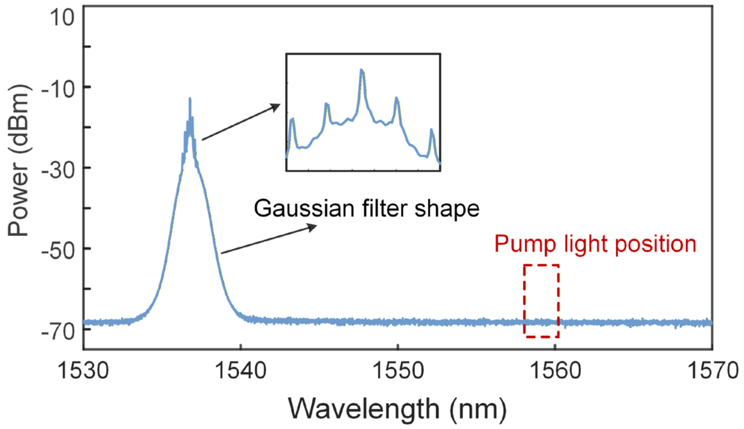

Fig. R27. Output spectrum after the filter-amplify-filter.

(Line 506 in revised manuscript)

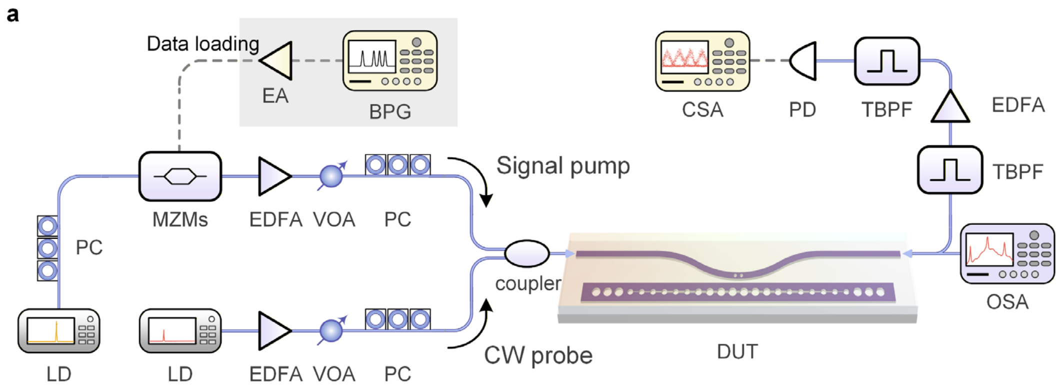

Fig. 3. Demonstration of two-mode all-optical Kerr switching. **a** Experimental setup for the all optical switching measurement. LD: laser device; PC: polarization controller; MZMs: Mach Zehnder modulators; BPG: bit pattern generator; EA: electrical amplifier; EDFA: erbium-doped fiber amplifier; VOA: variable optical attenuator; DUT: device under test; TBPF: tunable bandpass filter; PD: Photodiode; CSA: communication signal analyzer; OSA: optical spectrum analyzer. An EDFA is used to amplify the output probe signal [67]. …

…

[67] Gu, W. et al. All-optical complex-valued convolution based on four-wave mixing. *Optica* **11**, 64-72 (2024).

In addition, we will provide a detailed schematic of the BER measurement setup. In general, there are two commonly used methods for measuring BER performance. (i) The first method records the BER as a function of the optical power received by the photodetector (PD), as illustrated in Fig. **R28(a)**. This is the approach adopted in our measurements, and it naturally results in received powers reaching the 0 dBm level after optical amplification. (ii) The second method keeps the optical power at the PD constant while sweeping the input power to the EDFA to record the corresponding BER, as shown in Fig. **R28(b)**. Because we follow method (i), the plotted "received power"

corresponds to the PD input after EDFA, rather than the power within the nanocavity. We will include a detailed schematic of the BER measurement setup in the Supplementary Information to avoid ambiguity.

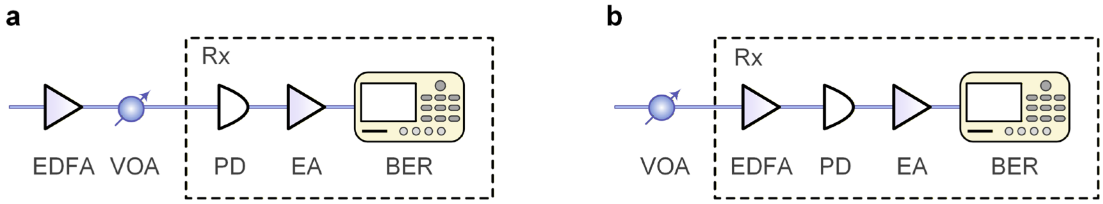

Fig. R28. Experimental setup for the BER measurements.

(Line 517 in revised Supplementary Information)

S9. Experimental Setup for Optical Measurement

The high-speed data generation and BER measurements are carried out using an integrated SHF communication test platform, consisting of a synthesized clock generator (SHF 78210 D), a bit-pattern generator (SHF 12104 A), and an error analyzer (SHF 11104 A). The synthesized clock generator provides a low-jitter reference clock that synchronizes both the transmitter and receiver, ensuring deterministic timing and accurate bit-by-bit error analysis. The bit pattern generator generates a PRBS sequence of length $2^7 - 1$ at the target symbol rate, which is operated in a cyclic mode to provide a continuous data stream for driving the modulator. Meanwhile, the error analyzer performs real-time BER counting by synchronizing the detected electrical signal with the internally generated reference PRBS pattern. BER counting is initiated only after successful pattern acquisition and stable lock are achieved, ensuring reliable synchronization prior to real-time error counting.

On the receiver side, the switched probe signal is detected using a high-speed photodetector (XPDV2120R, 40-GHz electrical bandwidth), followed by broadband electrical amplification and direct connection to the SHF error analyzer, as shown in Fig. **S21**. By adjusting a variable optical attenuator (VOA) placed before the photodetector (PD), the BER is measured as a function of the received optical power at the PD, as shown in Fig. **4b**. The overall electrical bandwidth of the detection chain is sufficient to support the maximum tested symbol rate, ensuring that the measured BER performance is not limited by receiver bandwidth.

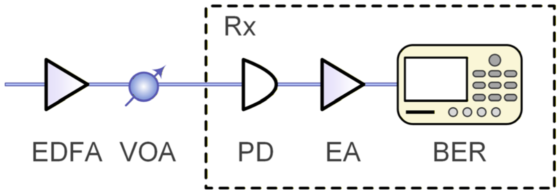

Fig. S21. Experimental setup for the BER measurements.

The PRBS sequence (length $2^7 - 1$) is generated in a continuous cyclic mode and measured over an extended duration. At a symbol rate of 40 GBaud and a measurement time of 2 s, a total of more than 8×10^{10} bits are analyzed. With zero

errors observed, Poisson statistics yield an upper BER bound of 3.75×10^{-11} at a 95% confidence level, which is well below the commonly accepted error-free criterion of 10^{-9}, thereby substantiating the reported error-free operation.

Reviewer's Q3:

3. At line 216, the words "switching time" would be better written as "switching window" or "switching time width" because the switching time typically correspond to the rise/fall time.

Author's A3:

We sincerely thank the reviewer for the helpful clarification regarding the terminology. We agree that "switching time" when used without qualification, typically refers to the rise/fall time of the switching transient. To avoid ambiguity, we have revised the term to "switching window" throughout the manuscript and Supplementary Information. We believe this wording more accurately reflects the meaning in our context.

(Line 223 in revised manuscript)

Figure **3b** presents the input (upper panel) and output (bottom panel) spectrum measured by an optical spectrum analyzer (OSA), corresponding to an on-chip input peak power of 7.5 mW for the pump light. With an estimated switching window from the duty cycle as well as the signal rate, i.e., $33\%/(40\ \mathrm{Gb/s}) \approx 8.3\ \mathrm{ps}$, the corresponding switching energy (defined as the energy of each "1" bit of pump light on-chip) is 63 fJ/bit, calculated as $7.5\ \mathrm{mW} \times 8.3\ \mathrm{ps} \approx 63\ \mathrm{fJ/bit}$. …

(Line 556 in revised Supplementary Information)

…The estimated switching window is approximately 11 ps, calculated from the signal rate and duty cycle (i.e., $33\%/(30\ \mathrm{Gb/s}) \approx 11\ \mathrm{ps}$), corresponding to a switching energy of 80 fJ/bit (i.e., $7.2\ \mathrm{mW} \times 11\ \mathrm{ps} \approx 80\ \mathrm{fJ/bit}$). …

Reviewer's Q4:

4. In Fig. 5, the results from [1] were not shown, although [1] shows a very low switching energy (sub-fJ). Could the authors comment on this?

Author's A4:

We sincerely thank the reviewer for pointing out reference [1], which indeed reports an impressive sub-femtojoule switching energy. Specifically, Ref. [1] employs an engineered InGaAsP composition with a bandgap slightly below the operating wavelength, enabling strong carrier-induced nonlinearity (band-filling dispersion and two-photon absorption or linear absorption). Using this mechanism in combination with an ultrasmall H0 photonic-crystal nanocavity, the authors successfully demonstrate low-energy switching within a few tens of picoseconds, reporting switching energies of 0.42 fJ and 0.66 fJ for switching contrasts of 3 dB and 10 dB, respectively. In addition, a representative 4-bit pulse-extraction demonstration at 40 Gbps is presented, highlighting the strong potential of this platform for ultrafast optical signal manipulation.

The reason that Ref. [1] is not included in the radar plot of Fig. **5**, is not related to its performance quality, but rather to the difficulty of making a fair and quantitative

comparison within a unified multi-parameter framework. While the work demonstrates an impressive 4-bit pulse-extraction experiment at 40 Gbps, it does not provide a complete set of quantitative metrics—such as switching contrast, switching time, and peak pump power—under identical operating conditions (e.g., at a specified energy such as 10.2 fJ/bit). As a result, directly mapping the reported results onto all axes of the radar plot would require assumptions that are not explicitly supported by the published data. Moreover, if the pump-probe switching results reported in Ref. [1] are used instead, the corresponding duty-cycle metric would appear extremely low due to the ultrashort-pulse excitation scheme. Including such a value in the radar plot would bias the comparison in a way that does not accurately reflect either the strengths of this excellent work or its intended operating regime. For these reasons, we believe that forcing Ref. [1] into the same radar-plot framework would risk an unfair or misleading comparison.

That said, we fully agree with the reviewer that such landmark demonstrations should be explicitly acknowledged and placed in a broader comparative context. To address this point, we have added a new supplementary table (Table **S1**) that systematically summarizes the performance metrics of a wider range of reported chip-scale all-optical switches across different material platforms and nonlinear mechanisms. This table includes key parameters such as switching energy, switching speed, and switching contrast, as well as criterions that evaluates how closely the experimental results align with all-optical signal processing, i.e., duty cycle and repetition rate. These parameters and criterions roughly reflect the practical application potential of the devices, allowing readers to appreciate the strengths and trade-offs of different approaches without forcing them into a single normalized radar representation. The supplementary table has been added to Section **S12** of the revised Supplementary Information. We believe this approach provides a more balanced and informative comparison.

(Line 599 in revised Supplementary Information)

S12. Performance Comparison of On-chip All-optical Switches

A variety of chip-scale all-optical switches has been demonstrated across different material platforms and nonlinear mechanisms. Here, we provide a detailed comparison of four key performance metrics: peak power, switching energy, switching time, and switching contrast, as well as criterions that evaluates how closely the experimental results align with all-optical signal processing, i.e., duty cycle and repetition rate, as shown in Table **S1**. The criterions roughly reflect the practical application potential of the devices. The basic demos involve measuring the dynamic characteristics of the switching device using pump-probe techniques (p-p), where the input signal has a very low repetition rate. As the repetition rate increases, the average dissipated power also increases, potentially introducing thermal effects that can impact device performance. Additionally, the incomplete recovery of the signal waveform could affect subsequent bit streams, a phenomenon known as patterning effects. The second level involves using fixed-pattern data streams with high duty cycles and high repetition rate, such as predefined 4-bit repeating data sequences, which are more relevant for practical applications. The demos mostly

close to practical applications build upon the second by applying PRBS data streams for bit-error rate measurements, ensuring the performance of device in practical, real-world scenarios.

Table S1 Comparison of the performance of various on-chip all-optical switches.

Principle	Peak Power	Switching Time	Switching Energy	Switching Contrast	Duty Cycle (%)	Repetition Rate
BFD+TPA/LA [16]	47 µW *	35 ps	0.66 fJ/bit	10 dB	1.4×10^{-2} *	10 MHz p-p
	NA	NA	10.2 fJ/bit	NA	NA	40 Gbps 4-bit pulses
BFD+FCE [17]	10.33 mW *	NA	310 fJ/bit	3 dB	2.4×10^{-1} *	80 MHz p-p
Saturable absorption [18]	167 mW *	260 fs	35 fJ/bit	3.5 dB	2.1×10^{-4} *	10 MHz p-p
$\chi^{(2)}$ nonlinearity [19]	2 W *	46 fs	80 fJ/bit	3 dB	1×10^{-3} *	250 MHz p-p
FCE [20]	5 mW *	30 ps	100 fJ/bit	3 dB	NA	5 Gbps 4-bit pulses
FCE [21]	60 mW *	70 ps	460 fJ/bit	NA	7.7×10^{-3} *	10 MHz p-p
Kerr effect [22]	370 mW *	NA	1124 fJ/bit *	7.7 dB	12.81 *	42.7 Gbps 2^{31}-1 PRBS
Kerr effect [23]	264 mW *	18 ps	720 fJ/bit	NA	2.7×10^{-2} *	100 MHz p-p
ISBT [24]	4.3 W *	1.2 ps	520 fJ/bit	7 dB	9.12×10^{-4} *	76 MHz p-p
**This work**	**7.5 mW**	**9 ps**	**63 fJ/bit**	**8.3 dB**	**33**	**40 Gbps 2^7-1 PRBS (Error-free)**

Notes: NA—not available, BFD—band-filling dispersion, TPA—two-photon absorption, LA—linear absorption, FCE—free-carrier effect, ISBT—inter-sub-band transition, p-p—pump-probe measurement, PRBS—Pseudo-Random Binary Sequence, *—calculated based on available data: The switching energy is defined as the product of the on-chip peak pump power and the effective temporal width of the input pump "1"-bit, while the duty cycle is given by the product of this pulse width and the pulse repetition rate.

…

[16] Nozaki, K. et al. Sub-femtojoule all-optical switching using a photonic-crystal nanocavity. *Nat. Photon*. **4**, 477–483 (2010).

[17] Takiguchi, M. et al. All-Optical InAsP/InP Nanowire Switches Integrated in a Si Photonic Crystal. *ACS Photon*. **7**, 1016-1021 (2020).

[18] Ono, M. et al. Ultrafast and energy-efficient all-optical switching with graphene-loaded deep-subwavelength plasmonic waveguides. *Nat. Photon*. **14**, 37–43 (2020).

[19] Guo, Q. et al. Femtojoule femtosecond all-optical switching in lithium niobate nanophotonics. *Nat. Photon*. **16**, 625–631 (2022).

[20] Moille, G. et al. Integrated all-optical switch with 10 ps time resolution enabled by ALD. *Laser Photonics Rev*. **10**, 409 (2016).

[21] Tanabe,T. et al. Fast all-optical switching using ion-implanted silicon photonic crystal nanocavities. *Appl. Phys. Lett*. **90**, 031115 (2007).

[22] Koos, C. et al. All-optical high-speed signal processing with silicon-organic hybrid slot waveguides. *Nat. Photon*. **3**, 216–219 (2009).

[23] Jason S. et al. Picosecond all-optical switching in hydrogenated amorphous silicon microring resonators. *Opt. Express* **22**, 3797-3810 (2014).

[24] Hu X. et al. Picosecond and low-power all-optical switching based on an organic photonic-bandgap microcavity. *Nat. Photon*. **2**, 185–189 (2008).

Reviewer's Q5:

5. It would be helpful for readers if the authors could discuss the reason of selecting the duty cycle of 33%. Also, the discussion of future improvements (lowering the peak power, increasing the duty cycle and so on) would be helpful for the readers.

Author's A5:

We sincerely thank the reviewer for raising this important question regarding the choice of duty cycle and the prospects for future improvements. The choice of a 33% duty cycle in our experiment is primarily determined by the signal-generation scheme used to produce high-quality 40-Gbit/s RZ-OOK data streams, rather than by any intrinsic limitation of the device itself. In our setup, the RZ-OOK signal is generated using two cascaded Mach-Zehnder modulators (MZMs). The first MZM is driven by a 40-GHz sinusoidal clock from the bit-pattern generator (BPG), generating a periodic sinusoidal intensity modulation that serves as the optical envelope for RZ pulse carving. The second MZM, driven by the synchronized OOK data pattern from the BPG, modulates this envelope to generate the final RZ-OOK signal. Under this widely used scheme—without employing additional pulse-compression or time-multiplexing techniques—the resulting RZ-OOK signal inherently exhibits a duty cycle of approximately 33%, which is a standard value in practical high-speed communication systems. Therefore, the duty cycle adopted in this work reflects a conventional and representative operating condition, rather than an arbitrary design choice.

Importantly, the demonstrated device operation is not fundamentally restricted to a 33% duty cycle. As discussed in Section **S8** of the Supplementary Information, the thermo-optic response of the polymer-filled nanocavity is governed by the average optical power and evolves on timescales much slower than the bit period at 40 GBaud (i.e., 25 ps), whereas the Kerr nonlinearity responds instantaneously at the bit level. This clear separation of timescales implies that increasing the duty cycle—while maintaining the same average power—does not introduce additional thermal penalties and can, in principle, further reduce the required peak power for switching.

Several practical routes exist to enable higher duty-cycle operation and lower peak power in future implementations. Building upon the discussion in the original Discussions and Conclusions section, we have further expanded and discussed these improvements in the revised Supplementary Information.

(1) On the system side, alternative modulation formats and pulse-shaping techniques, such as carrier-suppressed RZ, multi-level modulation, or external pulse compression schemes, can readily generate higher-duty-cycle data streams.

(2) On the device side, further enhancement of the intrinsic cavity Q-factor can be achieved by minimizing scattering losses at the slot sidewalls through optimized fabrication processes, including annealing, thermal oxidation or optimized etching processes. A higher intrinsic Q-factor directly strengthens the Fano resonance contrast and lowers the switching energy. In parallel, further reduction of the slot width provides an additional route to enhance the effective Kerr nonlinearity by strengthening optical field confinement and reducing the effective mode volume. The combined improvement of intrinsic Q-factor and modal confinement would directly increase the effective Kerr interaction strength, enabling the same switching contrast at lower peak pump power.

(3) For future scaling toward higher power handling or broader operating scenarios, the thermal robustness of the nonlinear polymer can be further enhanced through materials and process engineering. Building on recent advances in thermally stable and crosslinked organic polymer systems [*Chemistry of Materials* **37**, 12 (2025)], the

nonlinear polymer may be spin-coated in combination with an imprint resist or cross-linkable host matrix, followed by ultraviolet curing. Such approaches have been shown to increase the glass transition temperature and suppress thermally induced polymer reflow, thereby providing a viable pathway for improving the thermal tolerance of hybrid silicon-organic nanophotonic devices.

(4) In addition, for long-term deployment or operation under higher duty-cycle excitation, system-level stabilization strategies can be readily implemented. These include active feedback control of the pump wavelength or optical power, chip-level temperature control, and improved packaging and heat dissipation designs, which together can further enhance operational stability under realistic environmental fluctuations.

Taken together, these considerations indicate a clear and realistic pathway toward higher duty-cycle, lower-peak-power, and more energy-efficient operation in future generations of the proposed SOH nanophotonic switch.

(Line 592 in revised Supplementary Information)

…For future implementations targeting extended deployment times or more demanding environments, additional stabilization strategies—such as active feedback control of the pump wavelength or optical power, chip-level temperature control, and optimized device packaging—can be readily incorporated to further enhance system robustness. …

(Line 488 in revised Supplementary Information)

…For future scaling toward higher power handling or broader operating scenarios, the thermal robustness of the nonlinear polymer can be further enhanced through materials and process engineering. Building on recent advances in thermally stable and crosslinked organic polymer systems [14], the nonlinear polymer may be spin-coated in combination with an imprint resist or cross-linkable host matrix, followed by ultraviolet curing. Such approaches have been shown to increase the glass transition temperature and suppress thermally induced polymer reflow, thereby providing a viable pathway for improving the thermal tolerance of hybrid silicon-organic nanophotonic devices. …

…

[14] Xu, H. et al. Ultrahigh Performance Cross-Linkable Organic Electro-Optic Material for Hybrid Modulators. *Chemistry of Materials* **37**, 12 (2025).
